# Strengthening the Reporting of Observational Studies in Epidemiology for respondent-driven sampling studies: “STROBE-RDS” statement

**DOI:** 10.1016/j.jclinepi.2015.04.002

**Published:** 2015-12

**Authors:** Richard G. White, Avi J. Hakim, Matthew J. Salganik, Michael W. Spiller, Lisa G. Johnston, Ligia Kerr, Carl Kendall, Amy Drake, David Wilson, Kate Orroth, Matthias Egger, Wolfgang Hladik

**Affiliations:** a Department of Infectious Disease Epidemiology, London School of Hygiene and Tropical Medicine, UK; b US Centers for Disease Control and Prevention, Atlanta, GA, USA; c Department of Sociology and Office of Population Research, Princeton University; d Department of Global Community Health and Behavioral Sciences, Tulane University School of Public Health and Tropical Medicine, USA; e Global Health Science, University of California San Francisco, USA; f Departamento de Saude Comunitaria, Universidade Federal do Ceara, Fortaleza, Ceara, Brazil; g World Bank, USA; h Institute of Social & Preventive Medicine (ISPM), University of Bern, Switzerland

**Keywords:** Practice guidelines as topic, Research design, Epidemiologic research design, Humans, Cross-sectional studies, Guidelines as topic/standards, Epidemiologic studies, Observation/methods, Biomedical research/methods, Guidelines as topic, Observation/methods, Publishing/standards

## Abstract

**Objectives:**

Respondent-driven sampling (RDS) is a new data collection methodology used to estimate characteristics of hard-to-reach groups, such as the HIV prevalence in drug users. Many national public health systems and international organizations rely on RDS data. However, RDS reporting quality and available reporting guidelines are inadequate. We carried out a systematic review of RDS studies and present Strengthening the Reporting of Observational Studies in Epidemiology for RDS Studies (STROBE-RDS), a checklist of essential items to present in RDS publications, justified by an explanation and elaboration document.

**Study Design and Setting:**

We searched the MEDLINE (1970–2013), EMBASE (1974–2013), and Global Health (1910–2013) databases to assess the number and geographical distribution of published RDS studies. STROBE-RDS was developed based on STROBE guidelines, following Guidance for Developers of Health Research Reporting Guidelines.

**Results:**

RDS has been used in over 460 studies from 69 countries, including the USA (151 studies), China (70), and India (32). STROBE-RDS includes modifications to 12 of the 22 items on the STROBE checklist. The two key areas that required modification concerned the selection of participants and statistical analysis of the sample.

**Conclusion:**

STROBE-RDS seeks to enhance the transparency and utility of research using RDS. If widely adopted, STROBE-RDS should improve global infectious diseases public health decision making.

**What is new?**

**Key findings**
• Strengthening the Reporting of Observational Studies in Epidemiology for respondent-driven sampling studies (STROBE-RDS) was developed based on STROBE statement following published Guidance for Developers of Health Research Reporting Guidelines. The STROBE-RDS checklist includes modifications to 12 of the 22 items on the STROBE checklist. The two key areas that required modification concerned the selection of participants and the statistical analysis of the sample.

**What this adds to what was known?**
• Respondent-driven sampling (RDS) is a new data collection methodology used to estimate characteristics of hard-to-reach groups, such as the HIV prevalence in drug users. Many national public health systems and international organizations rely on RDS data. RDS reporting quality and available reporting guidelines are inadequate. STROBE-RDS is a checklist of essential items to present in RDS publications.

**What is the implication and what should change now?**
• The STROBE-RDS statement seeks to enhance the transparency and utility of research using RDS. If widely adopted, the STROBE-RDS checklist should improve public health decision making in infectious diseases.

## 1 Introduction

Hidden or hard-to-reach population subgroups are often key to the maintenance of infectious diseases in human populations [1]. However, it is often difficult to investigate the factors that drive transmission in these groups by using commonly used epidemiologic methods of data collection because of the lack of an adequate sampling frame [2]. Researchers have therefore typically resorted to various types of convenience sampling to gather data on hidden populations [3]. Although convenience sampling has its advantages, this approach is unable to generate unbiased population-based estimates of infection prevalence and risk factors. In an attempt to address these limitations, respondent-driven sampling (RDS), a variant of a link-tracing design, was proposed in 1997 [4].

RDS studies are characterized by both a specific data collection method and specific statistical analysis methods. Key features of the data collection include: (1) a small proportion of the sample is recruited by the researcher (i.e., the “seeds”) and a large proportion of the sample is recruited (in recruitment “waves”) by other members of the target population to whom they have a social relationship; (2) recording of recruitment connections between respondents (e.g., who recruited whom); (3) the maximum number of people that each participant can recruit is determined by the researcher by giving out a limited number of recruitment “coupons”; (4) respondents are compensated for participating in the study and recruiting others into the study. Collectively, these features often make RDS an efficient data collection method [5]. Although often efficient, the RDS data collection method produces multiple challenges for the analysis. First, because most of the sampling is conducted by respondents, assumptions about the sampling process are needed. Second, under a variety of assumptions, not all members of the target population will have the same probability of selection, so this probability is typically estimated using a combination of modeling assumptions and study data. Finally, because sampling happens through pre-existing relationships, the observations are not independent. These challenges make point and variance estimation from RDS data more complex than from other forms of sampling. Numerous approaches have been developed, and more are currently under development [6], [7], [8], [9], [10], [11], [12].

Since its introduction in 1997 [4], there has been a rapid increase in the number of surveys of hidden or hard-to-reach populations using the RDS methodology, primarily of individuals at risk of sexually or parenterally transmitted infections including HIV [5], [13], but also on topics as diverse interpersonal violence [13] and strategies for improving cancer screening recruitment [14]. Many countries including the United States, Ukraine, Vietnam, Mauritius, Morocco, and Brazil use RDS as part of their national public health systems, and data from RDS studies are used by major public health organizations including the US Centers for Disease Control and Prevention, the Joint United Nations Programme on HIV/AIDSs, and the Global Fund to Fight AIDS, Tuberculosis and Malaria. The National Institutes of Health alone has awarded around $100 million in funding to projects using RDS and its variants [15].

Making sense of the rapidly increasing amount of data collected using RDS [5], [16] is crucial to the integration of this information into the practice of medicine and public health. However, the assessment of the strengths and weaknesses of RDS data and methods has been limited by the inadequate reporting of RDS studies. An assessment of 22 randomly selected RDS studies has recently been carried out [17]. The assessment found that overall only around one-third of items sought were reported. Key details of the sampling and statistical methods were particularly poorly reported, including the methods of seed selection (reported in 45% of studies), the number of recruits from each seed and number of recruits in each recruitment wave (33%), the details of the recruitment venues (33%), eligibility criteria for seeds (<20%), wording of network size questions (<20%), if seeds were included in the analysis (<20%), how participants were trained to recruit others (0%), and an explanation for differences between unadjusted and adjusted estimates (0%) [17].

To improve the quality of reporting of observational epidemiologic studies, the Strengthening the Reporting of Observational studies in Epidemiology (STROBE) statement [18], [19], [20], [21] and extensions [22], [23] were developed. However, the STROBE statement is inadequate for reporting RDS studies because of the major differences in the RDS sampling and estimation procedures. Our aims are to present a systematic review of the number of RDS studies and present the “STROBE-RDS” statement, a checklist of essential items to present in RDS publications, justified and supported by a stand-alone explanation and elaboration document.

## 2 Methods

### 2.1 Systematic literature review of RDS studies

A systematic literature review was carried out purely to assess the number of published RDS studies and summarize their geographical distribution. Briefly, we searched the MEDLINE (1970–2013), EMBASE (1974–2013), and Global Health (1910–2013) databases and asked experts for their collections of relevant articles. Studies conducted in any country, in any language, among any study population were included; reviews, editorials, commentaries, and methodological articles were excluded. A previous assessment had identified the inadequacy of previous RDS reporting [17], and therefore, further details on RDS reporting were not collected. Full details are shown in the Supplementary Material (Document 1 at www.jclinepi.com).

### 2.2 Statement development

The STROBE-RDS statement was developed after the Guidance on Health Research Guidelines of Moher et al [24]. The initial need for the RDS reporting guidelines was identified in RDS expert and stakeholder discussions at an RDS symposium in 2011 [25]. This was followed by a systematic evaluation of the reporting of RDS studies that concluded that the reporting of RDS studies was inadequate [17]. Existing guidelines were then reviewed, and the most suitable was the STROBE statement [18], [19], but this was assessed to be inadequate for reporting RDS studies because of the major differences in the RDS sampling and estimation procedures. The vast majority of existing RDS studies use a cross-sectional study design. Therefore, this statement is an extension of the STROBE guidelines [18], the STROBE explanation and elaboration document [19], and the STROBE checklist for cross-sectional studies [20]. Version 1 of the STROBE-RDS checklist was distributed for consultation by posting on the Equator Network Web site [26] and the RDS listserv [27] and by sending to known experts. Themes emerging from the feedback included: strong support for the initiative and a request to restrict the scope of the guidelines to cross-sectional epidemiologic studies that seek to generate representative estimates for the target population. The checklist was revised based on this feedback, and version 2 was published in early October 2012 [28]. Version 2 of the checklist was piloted during October 2012 by using it to guide manuscript drafting and was sent to the STROBE group for feedback [18]. Researchers (*n* = 5) piloting the checklist provided much useful feedback and requested a stand-alone checklist and supporting document. This and other feedback was used to develop version 3 of the checklist, which was discussed at a 2-day face-to-face meeting in New Orleans in October 2012 [29]. A list of potential meeting invitees was drawn up by a subset of coauthors (R.G.W., A.J.H., and W.H.) after consultation with STROBE initiative members, statisticians, epidemiologists, and empirical RDS researchers. Potential invitees were categorized into three groups: statisticians/survey methodologists, epidemiologists/empirical RDS researchers, and journal editors. Fifty percent (2 of 4), 46% (6 of 13), and 0% (0 of 2), respectively, of meeting invitees in these three groups participated (11 meeting attendees in total). Participants were sent the draft checklist, a summary of the previous RDS reporting [17] and the guidelines by Moher et al. [24]. At the meeting, each draft checklist item was presented and discussed in turn and edited on screen in real time until agreement on the final version of the checklist was reached by consensus and is presented in this manuscript. Consensus was defined as asking verbally all participants if they agreed to the written text for each item. There were no items on which consensus was not reached during the meeting. After the face-to-face meeting, this summary manuscript was drafted by the authors, and to accompany the checklist, an “Explanation and Elaboration” document was developed and revised based on feedback from experts and the STROBE group and is presented in the Supplementary Material (Document 2 at www.jclinepi.com).

### 2.3 Statement scope

The scope of the “STROBE-RDS” statement is limited to (1) epidemiologic studies (the scope of the original STROBE guidelines), (2) cross-sectional studies (the most common RDS study design to date), and (3) RDS studies that seek to generate representative estimates for the target population (currently the most contentious and potentially most policy-relevant use of RDS). Furthermore, as RDS is both a sampling and a data analysis method, guidelines for reporting on both aspects of RDS are provided. Finally, in response to feedback from researchers piloting the STROBE-RDS checklist, we aim to provide a self-contained statement that minimizes the need to refer to other documents when reporting on an RDS study.

## 3 Results

**Fig. 1:**
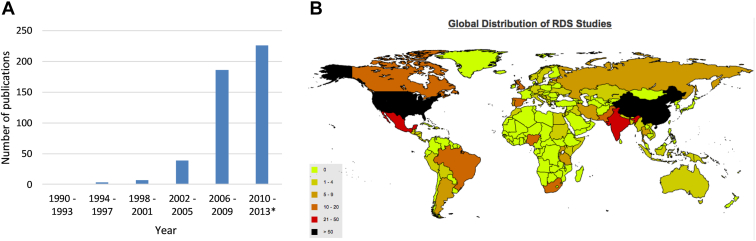
(A) Number of published peer-reviewed studies using respondent-driven sampling 1990–July 2013. * = part year. (B) World map showing number of published peer-reviewed studies using respondent-driven sampling 1990–July 2013, by country.

The systematic literature review and input from experts identified that globally over 460 peer-reviewed publications have reported using RDS since the mid-1990s, with most published since 2006 (Fig. 1A). Fig. 1B shows the global distribution of study locations. RDS studies have been conducted in 69 countries, including the United States of America (151 studies), China (70), India (32), Mexico (22), and South Africa (16). The articles came from 141 different journals. Most journals (91) had published either one or two articles included in the review. Nine journals had published 10 or more articles (or conference abstracts). Supplementary Material (Document 1 at www.jclinepi.com) provides more details of review methods, results, and included and excluded articles.

In Table 1, we present the proposed STROBE-RDS statement checklist, a modification of the STROBE statement checklist for cross-sectional studies [20]. The left column lists the original STROBE checklist for cross-sectional studies. The right column summarizes the reporting recommendation. A three-column version of the checklist, highlighting the changes from the original STROBE checklist, is shown in the supplementary information, along with an explanation and elaboration document [Supplementary Material (Document 2 at www.jclinepi.com)]. As there is considerable variation in the use of terms and definitions across the disciplines in which RDS is used, we also present a list of suggested RDS terms and definitions to be used when reporting RDS studies (Box 1).

The STROBE-RDS checklist provides modifications to 12 of the 22 items on the STROBE checklist. The two key areas requiring modification concerned the selection of participants and the statistical analysis of the sample. These modifications are summarized below.

### 3.1 Selection of participants

As members of the target population, not researchers, recruit most study participants in RDS studies, details of the formative research conducted before the study (T5b) and of how participants were trained to recruit others (6a) should be reported. Key details of the recruitment process should also be reported, including the number of coupons issued per person, any time limits for referral (6a), procedures of seed selection (6b), the exact wording of personal network size question(s) (6d), the incentives for participation and recruitment (6e), and how the recruiter–recruit relationship was tracked (7b). Variation in study procedures during data collection should also be reported (6c). Methods to assess eligibility and reduce repeat enrollment should be described (8b) so that other researchers can understand the data collection process and any biases it might introduce. Authors should report reasons for nonparticipation at each stage (e.g., not eligible, does not consent, decline to recruit others) (13b), the number of coupons issued and returned (13d), the number of recruits by seed and number of recruitment waves for each seed (13e), any recruitment challenges (e.g., commercial exchange of coupons, imposters, duplicate recruits), and how they were addressed (13f) (Table 1, items 5b, 6a,b,c,d,e, 7b, 8b, 13b,d,e,f).

### 3.2 Statistical analysis

Several different estimators exist for estimating the prevalence of a specific trait (e.g., HIV prevalence) from RDS data [6], [7], [8], [9], [10], [11], [12]. There are also a number of different methods for producing confidence intervals around these estimates [8], [30], [31], [32]. Evaluations of these methods have been equivocal [33], [34], [35], and the best estimator may depend on specific features of a study [36]. At this time, there is no consensus that one estimator should be universally used. As such, we recommend authors clearly describe the statistical methods used, including those to adjust for sample design, both when making estimates (Table 1, 12a,b) and when quantifying the uncertainty in those estimates (16a,c). As the utility of the various RDS estimators is unknown, we recommend reporting unadjusted and study design–adjusted estimates and, if applicable, confounder-adjusted estimates and their precision (16a). If adjustment of the primary outcome leads to marked changes, information on factors causing the changes (e.g., personal network sizes, recruitment patterns by group, key confounders) should be reported (16c) (Table 1, items 12a,b, 16a,c).

## 4 Discussion

The STROBE-RDS statement is a checklist of essential items that should be reported in RDS studies. The statement has several strengths. It is based on existing guidance for reporting observational studies, [18], [19], [20] was developed by an interdisciplinary group that included epidemiologists, statisticians, and empirical RDS researchers, and explicitly justifies changes from the original STROBE statement.

The decisions made for the conduct and data analysis of RDS studies will influence the representativeness of a study's results. The empirical evidence on how representative the study results are is limited, and improving the methodology is an active research area. Transparent reporting is essential for developing a better evidence base and improving RDS methods.

Based on feedback we received from researchers writing up RDS studies during the piloting of earlier versions of this checklist [28], we decided to provide a stand-alone STROBE-RDS checklist with full supporting documentation, rather than only providing a modified STROBE checklist. This should mean that researchers writing up RDS studies will be more likely to use these reporting guidelines. We encourage readers to read the accompanying explanation and elaboration document in full (supporting material) before embarking on writing up an RDS study.

The statement can be used by authors, peer reviewers, and editors to improve the reporting of RDS studies. We invite journals to endorse STROBE-RDS, and although STROBE-RDS will be published in English only once, we invite others to translate STROBE-RDS and to submit commentaries, editorials, or use other means to raise the awareness of the STROBE-RDS publication. The ability to provide information in Web supplements should alleviate concerns about the increased length of manuscripts resulting from following the guidelines. We welcome comments directed to the corresponding author or via the journal or Equator Network Web sites where the guidelines are also deposited [26]. These will be used to update this STROBE-RDS statement as RDS methods develop.

The STROBE-RDS statement does not prescribe or dictate how an RDS study should be designed or analyzed. Rather, it seeks to enhance the transparency of research using RDS to increase the understanding of individual studies and enable comparisons between studies. If widely adopted, the STROBE-RDS checklist should improve global public health decision making for infectious diseases. Further studies could assess the impact of STROBE-RDS on the transparency of RDS research and on global public health decision making.

### 4.1 Search strategy and selection criteria

We searched published (physically published or online), peer-reviewed literature accessible through July 2013 that reported using RDS. Studies from all countries were included. We conducted searches using MEDLINE (1970–2013), EMBASE (1974–2013), and Global Health (1910–2013). Search terms used included “respondent driven” or “respondent-driven” or “RDS.”

## Figures and Tables

**Table 1:** STROBE-RDS Statement Checklist

Item	#	Original STROBE checklist for cross-sectional studies	STROBE-RDS checklist
Title and abstract	1	(a) Indicate the study's design with a commonly used term in the title or the abstract	(a) Indicate *“respondent-driven sampling”* in the title or abstract
		(b) Provide in the abstract an informative and balanced summary of what was done and what was found	(b) Provide in the abstract an informative and balanced summary of what was done and what was found
Introduction			
Background/rationale	2	Explain the scientific background and rationale for the investigation being reported	Explain the scientific background and rationale for the investigation being reported
Objectives	3	State specific objectives, including any prespecified hypotheses	State-specific objectives, including any prespecified hypotheses
Methods			
Study design	4	(a) Present key elements of study design early in the article	(a) Present key elements of study design early in the article
			*(b) State why RDS was chosen as the sampling method*
Setting	5	(a) Describe the setting, locations, and relevant dates, including periods of recruitment, exposure, follow-up, and data collection	*(a) Describe the setting, locations, and relevant dates, including periods of recruitment and data collection*
			*(b) Describe formative research findings used to inform RDS study*
Participants	6	(a) Give the eligibility criteria and the sources and methods of selection of participants	(a) Give the eligibility criteria and the sources and methods of selection of participants. *Describe how participants were trained/instructed to recruit others, number of coupons issued per person, any time limits for referral*
			*(b) Describe methods of seed selection and state number at start of study and number added later*
			*(c) State if there was any variation in study procedures during data collection (e.g., changing numbers of coupons per recruiter, interruptions in sampling, or stopping recruitment chains)*
			*(d) Report wording of personal network size question(s)*
			*(e) Describe incentives for participation and recruitment*
Variables	7	(a) Clearly define all outcomes, exposures, predictors, potential confounders, and effect modifiers. Give diagnostic criteria, if applicable	*(a) If applicable,* clearly define all outcomes, *correlates*, predictors, potential confounders, effect modifiers, and diagnostic criteria
			*(b) State how recruiter–recruit relationship was tracked*
Data sources/measurement	8	(a) For each variable of interest, give sources of data and details of methods of assessment (measurement). Describe comparability of assessment methods if there is more than one group	(a) For each variable of interest, give sources of data and details of methods of *measurement.* Describe comparability of *measurement* methods if there is more than one group
			*(b) Describe methods to assess eligibility and reduce repeat enrollment (e.g., coupon manager software, biometrics)*
Bias	9	Describe any efforts to address potential sources of bias	Describe any efforts to address potential sources of bias
Study size	10	Explain how the study size was arrived at	Explain how the study size was arrived at
Quantitative variables	11	Explain how quantitative variables were handled in the analyses. If applicable, describe which groupings were chosen and why	Explain how quantitative variables were handled in the analyses. If applicable, describe which groupings were chosen and why
Statistical methods	12	(a) Describe all statistical methods, including those used to control for confounding	*(a) Describe all statistical methods, including those to account for sampling strategy (e.g., the estimator used) and, if applicable, those used to control for confounding*
			*(b) State data analysis software, version number, and specific analysis settings used*
		(b) Describe any methods used to examine subgroups and interactions	(c) Describe any methods used to examine subgroups and interactions
		(c) Explain how missing data were addressed	(d) Explain how missing data were addressed
		(d) If applicable, describe analytical methods taking account of sampling strategy	
		(e) Describe any sensitivity analyses	(e) Describe any sensitivity analyses
			*(f) Report any criteria used to support statements on whether estimator conditions or assumptions were appropriate*
			*(g) Explain how seeds were handled in analysis*
Results			
Participants	13	(a) Report the numbers of individuals at each stage of the study—for example, numbers potentially eligible, examined for eligibility, confirmed eligible, included in the study, completing follow-up, and analyzed	*(a) Report the numbers of individuals at each stage of the study—for example, numbers potentially eligible, examined for eligibility, confirmed eligible, included in the study, and analyzed*
		(b) Give reasons for nonparticipation at each stage	(b) Give reasons for nonparticipation at each stage *(e.g., not eligible, does not consent, decline to recruit others)*
		(c) Consider use of a flow diagram	(c) Consider use of a flow diagram
			*(d) Report number of coupons issued and returned*
			*(e) Report number of recruits by seed and number of RDS recruitment waves for each seed. Consider showing graph of entire recruitment network*
			*(f) Report recruitment challenges (e.g., commercial exchange of coupons, imposters, duplicate recruits) and how addressed*
			*(g) Consider reporting estimated design effect for outcomes of interest*
Descriptive data	14	(a) Give characteristics of study participants (e.g., demographic, clinical, social) and information on exposures and potential confounders	(a) Give characteristics of study participants (e.g., demographic, clinical, social) and, *if applicable,* information on *correlates* and potential confounders. *Report unweighted sample size and percentages, estimated population proportions or means with estimated precision (e.g., 95%* confidence interval*)*
		(b) Indicate the number of participants with missing data for each variable of interest	(b) Indicate the number of participants with missing data for each variable of interest
Outcome data	15	Report numbers of outcome events or summary measures	*If applicable,* report number of outcome events or summary measures
Main results	16	(a) Give unadjusted estimates and, if applicable, confounder-adjusted estimates and their precision (e.g., 95% confidence intervals). Make clear which confounders were adjusted for and why they were included	(a) Give unadjusted *and study design–adjusted* estimates and, if applicable, confounder-adjusted estimates and their precision (e.g., 95% confidence intervals). Make clear which confounders were adjusted for and why they were included
		(b) Report category boundaries when continuous variables were categorized	(b) Report category boundaries when continuous variables were categorized
		(c) If relevant, consider translating estimates of relative risk into absolute risk for a meaningful period	*(c) If adjustment of primary outcome leads to marked changes, report information on factors influencing the adjustments (e.g., personal network sizes, recruitment patterns by group, key confounders)*
Other analyses	17	Report other analyses done—for example, analyses of subgroups and interactions and sensitivity analyses	Report other analyses done—for example, analyses of subgroups and interactions, sensitivity analyses, *different RDS estimators and definitions of personal network size*
Discussion			
Key results	18	Summarize key results with reference to study objectives	Summarize key results with reference to study objectives
Limitations	19	Discuss limitations of the study, taking into account sources of potential bias or imprecision. Discuss both direction and magnitude of any potential bias	Discuss limitations of the study, taking into account sources of potential bias or imprecision. Discuss both direction and magnitude of any potential bias
Interpretation	20	Give a cautious overall interpretation of results considering objectives, limitations, multiplicity of analyses, results from similar studies, and other relevant evidence	Give a cautious overall interpretation of results considering objectives, limitations, multiplicity of analyses, results from similar studies, and other relevant evidence
Generalizability	21	Discuss the generalizability (external validity) of the study results	Discuss the generalizability (external validity) of the study results
Other information			
Funding	22	Give the source of funding and the role of the funders for the present study and, if applicable, for the original study on which the present article is based	Give the source of funding and the role of the funders for the present study and, if applicable, for the original study on which the present article is based

*Abbreviations*: STROBE-RDS, Strengthening the Reporting of Observational Studies in Epidemiology for respondent-driven sampling; #, number.

Italics highlight changes from STROBE statement checklist for cross-sectional studies [20]. Full details of modifications from [20] are shown in Table S1 in Supplementary Material at www.jclinepi.com.

## Box 1 Respondent-driven sampling key terms and definitions

**Candidate participant**—a coupon recipient who attempts to enroll in the study.

**Coupon**—an invitation to enroll in the RDS study that a participant can give to other people.

**Coupon recipient**—a person who receives a coupon.

**Equilibrium**—this term has inconsistent usage in the RDS community. The most common usage is that the observed sample composition matches the expected long-run sample composition assuming a specific model of the sampling process.

**Follow-up interview**—an interview where additional information is collected from the subset of participants who return to the study site a second time to collect recruitment incentives and/or biological test results.

**Homophily**—this term has inconsistent usage in the RDS community. Sometimes it is used to refer to the tendency for sample recruitments to occur between participants in the same social category and sometimes to refer to the tendency for relationships in the target population to occur between participants in the same social category.

**Main interview**—interview that is conducted of all participants where the main study information is collected.

**Participants**—members of the target population who have provided consent and completed the main interview.

**Participation incentive**—the money, goods, and/or services provided to participants for completing the main interview.

**Peer-recruited participant**—a participant recruited by a member of the target population.

**Personal network size (also called “degree”)**—the number of relationships a person has to members of the target population.

**Population estimate**—an estimate of a characteristic of the study population that takes into account the RDS sampling design.

**Recruitment incentive**—the money, goods, and/or services provided to participants for each new participant they are able to recruit.

**Recruitment tree (also chain)**—the set of all participants linked to a specific seed.

**Sample description**—a summary statistic of participants that does not take into account the RDS sampling design.

**Screening interview**—a short initial interview with people hoping to enroll in the study that seeks to verify membership in the target population and request consent.

**Seed**—a participant who is recruited by a researcher.

**Target population**—the set of people about whom the researchers wish to make estimates.

**Wave**—the set of participants a given number of recruitments from a seed.

*Abbreviation*: RDS, respondent-driven sampling.
