# Supplementary material for: Strengthening the Reporting of Observational Studies in Epidemiology for respondent-driven sampling studies: “STROBE-RDS” statement
Source: J Clin Epidemiol. 2015 Dec;68(12):1463–71. doi: 10.1016/j.jclinepi.2015.04.002 (PMC4669303; doi:10.1016/j.jclinepi.2015.04.002)
Supplement: Document 1 [file mmc1.doc]

**SUPPLEMENTARY MATERIAL #1**

**STRENGTHENING THE REPORTING OF OBSERVATIONAL STUDIES IN EPIDEMIOLOGY FOR RESPONDENT-DRIVEN SAMPLING STUDIES: *‘STROBE-RDS’* STATEMENT**

**SYSTEMATIC LITERATURE REVIEW OF RDS STUDIES**

**2015_06_13**

Richard G White[[1]](#footnote-2)*×, Avi J Hakim[[2]](#footnote-3)*, Matthew J Salganik[[3]](#footnote-4), Michael W SpillerError: Reference source not found, Lisa G Johnston[[4]](#footnote-5), Ligia Kerr[[5]](#footnote-6), Carl Kendall[[6]](#footnote-7), Amy DrakeError: Reference source not found, David Wilson[[7]](#footnote-8), Kate OrrothError: Reference source not found, Matthias Egger[[8]](#footnote-9), Wolfgang Hladik^Error: Reference source not found^

* Authors contributed equally to this work × Corresponding author

**ABSTRACT**

**SECTION 1: INTRODUCTION**

**SECTION 2: METHODS**

**SECTION 3: RESULTS SUMMARY**

**SECTION 4: DISCUSSION**

**SECTION 4: LIST OF INCLUDED STUDIES**

**SECTION 5: LIST OF EXCLUDED STUDIES – METHODOLOGICAL PAPERS**

**SECTION 6: LIST OF EXCLUDED STUDIES - REVIEWS**

**SECTION 7: LIST OF EXCLUDED STUDIES - EDITORIALS**

**ABSTRACT**

Background: The STROBE-RDS checklist has been developed as studies employing Respondent Driven Sampling (RDS) are proliferating in the literature.

Objective: The objective of this systematic review was to describe the global distribution of studies employing RDS and how the number of publications have changed over time.

Data Sources: Data were collected from MEDLINE, EMBASE and Global Health.

Eligibility Criteria: Studies claiming to use RDS for primary data collection in any study population from any country were eligible.

Results: Over 460 RDS studies have been conducted in 69 countries, with the most studies having been conducted in the US and China. Several studies have also been conducted in India, Mexico and South Africa.

Conclusions and implications of key findings: The widespread, and increasing, use of RDS methodology globally, combined with the limited reporting quality, indicates the need for STROBE-RDS guidelines for reporting of such studies.

**SECTION 1: INTRODUCTION**

The rationale and objective of this systematic review is to better understand the use globally of RDS methodology in epidemiological studies as evidenced by the reporting of such studies in the literature, either as peer-reviewed articles or presentations at international conferences. The main study question addressed by this review is where the methodology is being employed globally and how it may be changing over time*.* As such, the review does not limit included studies based on participants, interventions, comparisons, outcomes or study designs (PICOS).

**SECTION 2: METHODS**

We searched published (physically published or online), peer-reviewed literature accessible through July, 2013 that reported using respondent-driven sampling. Studies from all countries were included. We conducted searches using MEDLINE (1970 – 2013), EMBASE (1974 – 2013), and Global Health (1910 – 2013). Search terms used included “respondent driven” or “respondent-driven” or “RDS”. The combined search was narrowed in an attempt to capture relevant RDS studies by excluding such terms as “rapidly digestible starch”, “road-deposit sediment”, “reward deficiency syndrome”, “ant RD”, “renal damage and screening”, “rheumatoid diseases”, “repeated donors”, “registered dietitians”, “retinal detachment”, “renal duplex sonography”, “research documentation system”, “rubber dams”, “region of difference”, “gamma radiation”, “respiratory distress syndrome”, “respiratory infections”, “rate determining step”, “rate-determining step”, “rapid discharge sintering”, “risk difference”, “rate difference”, “reading disabilities”, “retinal degeneration slow”, “retina”, “RDS mice”, and “retired drug subsidy”. One person (KO) screened and selected the studies.

Eligible studies included those that claimed to use RDS techniques for primary data collection or modified RDS. Studies conducted in any country, in any language, among any study population were included. We excluded reviews, opinion pieces, editorials, commentaries and papers strictly addressing RDS methodology. In an attempt to detect more recent RDS studies, data for published abstracts from presentations at international conferences were included but no data from unpublished manuscripts, protocols, agency or technical reports or personal communication were included.

All relevant articles from the search were reviewed and the country and year of data collection were extracted. More information was not collected because of resource limitations and because an earlier study had highlighted the limitations of RDS study reporting (*Hafeez, S. (2012). A review of the proposed STROBE - RDS reporting checklist as an effective tool for assessing the reporting quality of RDS studies from the developing world. London, LSHTM.*). If the year of data collection was not available, the year of publication was used as a proxy, as was the case for some published conference abstracts. These data were then used to create a map, which shows the number of RDS studies conducted, by country. Studies were included in the map more than once if they collected data in more than one country. The list of articles and abstracts were shared with experts in the field to identify outstanding articles using RDS that were not identified by the search strategy.

**SECTION 3: RESULTS SUMMARY**

4562 articles were identified through the search of all three databases (Table 1). The titles and abstracts were scanned and duplicates (2360) and irrelevant articles (1716) were excluded, three of these were erratum for relevant articles. After excluding reviews, editorials and methodological papers, 442 papers remained for data extraction (Figure 1). Upon sharing the systematic review results with experts in the field, 19 additional relevant papers were identified and included, giving a total of 461 articles. 11 papers reported findings from multiple countries, which resulted in 516 studies in the global map (Figure 2).

The 461 papers identified for data extraction came from 141 different journals. The majority of the journals (91) had published either one or two papers included in the review. However, nine journals had published ten or more articles (or conference abstracts). These nine journals accounted for 39% of the studies included in the review (Table 2). In addition, the use of RDS has been increasing over time (Main text: Figure 1 A). Extraction and collation of data from the systematic review indicate RDS studies have been conducted in 69 countries. The largest number of published, peer-reviewed studies using RDS have been conducted in the US (151) and China (70). Large numbers of studies have also been conducted in India (32), Mexico (22) and South Africa (16).

**SECTION 4: DISCUSSION**

This systematic review indicates that studies using RDS methodology have taken place all over the world and have been increasing in number over time. The increase in RDS studies over time suggests we may have underestimated the number of RDS studies from 2010 onwards due to lag time in publication. In an attempt to address this, we incorporated published data from conference abstracts that met our study criteria. However, the distribution of studies globally should be accurate. As the use of this methodology has become more ubiquitous, the STROBE-RDS guidelines will ensure accurate and complete reporting of these types of studies globally.

**Table 1**: Literature search results by database

| **Search** | **Term** | **EMBASE** | **Medline** | **Global Health** |
| --- | --- | --- | --- | --- |
| 1 | Respondent driven | 494 | 473 | 277 |
| 2 | Respondent-driven | 494 | 473 | 277 |
| 3 | RDS | 5391 | 4160 | 472 |
| 4 | 1 or 2 or 3 | 5709 | 4465 | 660 |
| 5 | 4 and excluding the irrelevant terms as listed in methods | 2459 | 1699 | 404 |

**Table 2: Journals publishing RDS studies at a high frequency (ten or more)**

| **Journal** | **Number of Studies (%)** |
| --- | --- |
| AIDS | 11 (2.4) |
| AIDS and Behavior | 31 (6.7) |
| BMC Public Health | 12 (2.6) |
| Drug and Alcohol Dependence | 16 (3.5) |
| International Journal of STD and AIDS | 11 (2.4) |
| Journal of Urban Health | 28 (6.1) |
| PLoS One | 15 (3.3) |
| Sexually Transmitted Diseases | 13 (2.8) |
| Sexually Transmitted Infections | 41* (8.9) |
| **Total** | 178* (38.6) |

* Includes 18 conference abstracts.

**Figure 1: Flow diagram of global systematic review for RDS studies.**

EMBASE

Searched 7/25/13

2459

Medline

Searched 7/25/13

1699

Global Health

Searched 7/26/13

404

4562

Duplicates

2360

461 articles met our criteria (after adding 19 articles from expert knowledge) and data on author, country of data collection and year were extracted

Reviews

9

Irrelevant

1716

Editorials

15

Methodology

20

**516 studies were included in the map after adjusting for 11 articles that reported data from multiple countries**

**Figure 2: Map depicting number of RDS studies conducted through July 2013, by country**


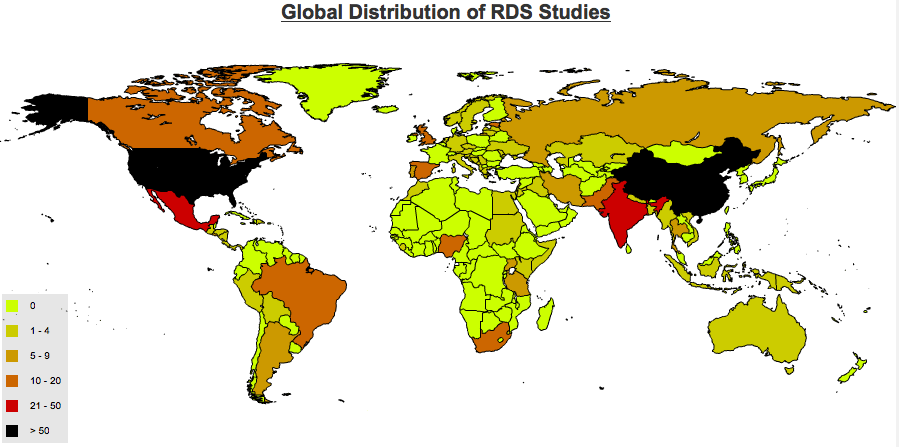


**SECTION 4: LIST OF INCLUDED STUDIES**

1. Bull, S. S., L. T. Breslin, E. E. Wright, S. R. Black, D. Levine and J. S. Santelli (1082). "Case study: An ethics case study of HIV prevention research on Facebook: the Just/Us study." Journal of Pediatric Psychology 36(10): 1082-1092.
2. Heckathorn, D. D. (1997). "Respondent-Driven Sampling: A New Approach to the Study of Hidden Populations." Social Problems 44(2): 174-199.
3. Clements-Nolle, K., R. Marx, R. Guzman and M. Katz (2001). "HIV prevalence, risk behaviors, health care use, and mental health status of transgender persons: Implications for public health intervention." American Journal of Public Health 91(6): 915-921.
4. Heckathorn, D. D. and J. Jeffri (2001). "Finding the beat: Using respondent-driven sampling to study jazz musicians." Poetics 28(4): 307-329.
5. Heckathorn, D. D., S. Semaan, R. S. Broadhead and J. J. Hughes (2002). "Extensions of respondent-driven sampling: A new approach to the study of injection drug users aged 18-25." AIDS and Behavior 6(1): 55-67.
6. Steele, M. S., E. Bukusi, C. R. Cohen, B. A. Shell-Duncan and K. K. Holmes (2004). "Male genital hygiene beliefs and practices in Nairobi, Kenya." Sexually Transmitted Infections 80(6): 471-476.
7. Carlson, R. G., J. Wang, R. S. Falck and H. A. Siegal (2005). "Drug use practices among MDMA/ecstasy users in Ohio: a latent class analysis." Drug and alcohol dependence 79(2): 167-179.
8. Falck, R. S., H. A. Siegal, J. Wang, R. G. Carlson and P. J. Draus (2005). "Nonmedical drug use among stimulant-using adults in small towns in rural Ohio." Journal of Substance Abuse Treatment 28(4): 341-349.
9. Fielder, O. and F. L. Altice (2005). "Attitudes toward and beliefs about prenatal HIV testing policies and mandatory HIV testing of newborns among drug users." AIDS and Public Policy Journal 20(3-4): 74-91.
10. Magis-Rodr√≠guez, C., K. C. Brouwer, S. Morales, C. Gayet, R. Lozada, R. Ortiz-Mondrag√≥n, E. P. Ricketts and S. A. Strathdee (2005). "HIV prevalence and correlates of receptive needle sharing among injection drug users in the Mexican-US border city of Tijuana." Journal of psychoactive drugs 37(3): 333-339.
11. Ramirez-Valles, J., D. D. Heckathorn, R. Vazquez, R. M. Diaz and R. T. Campbell (2005). "From networks to populations: The development and application of Respondent-Driven Sampling among IDUs and Latino gay men." AIDS and Behavior 9(4): 387-402.
12. Abdul-Quader, A. S., D. D. Heckathorn, C. McKnight, H. Bramson, C. Nemeth, K. Sabin, K. Gallagher and D. C. Des Jarlais (2006). "Effectiveness of respondent-driven sampling for recruiting drug users in New York City: findings from a pilot study." Journal of urban health : bulletin of the New York Academy of Medicine 83(3): 459-476.
13. Clements-Nolle, K., R. Marx and M. Katz (2006). "Attempted suicide among transgender persons: The influence of gender-based discrimination and victimization." Journal of Homosexuality 51(3): 53-69.
14. Draus, P. and R. Carlson (2006). "Needles in the haystacks: The social context of initiation to heroin injection in rural Ohio." Substance Use and Misuse 41(8): 1111-1124.
15. Evans-Campbell, T., T. Lindhorst, B. Huang and K. L. Walters (2006). "Interpersonal violence in the lives of urban American Indian and Alaska Native women: implications for health, mental health, and help-seeking." American journal of public health 96(8): 1416-1422.
16. Frost, S. D. W., K. C. Brouwer, M. A. Firestone Cruz, R. Ramos, M. E. Ramos, R. M. Lozada, C. Magis-Rodriguez and S. A. Strathdee (2006). "Respondent-driven sampling of injection drug users in two U.S.-Mexico border cities: Recruitment dynamics and impact on estimates of HIV and syphilis prevalence." Journal of Urban Health 83(1 SUPPL.): i83-i97.
17. Johnston, L. G., K. Sabin, M. T. Hien and P. T. Huong (2006). "Assessment of respondent driven sampling for recruiting female sex workers in two Vietnamese cities: Reaching the unseen sex worker." Journal of Urban Health 83(1 SUPPL.): i16-i28.
18. McKnight, C., D. Des Jarlais, H. Bramson, L. Tower, A. S. Abdul-Quader, C. Nemeth and D. Heckathorn (2006). "Respondent-driven sampling in a study of drug users in New York City: Notes from the field." Journal of Urban Health 83(1 SUPPL.): i54-i59.
19. Platt, L., M. Wall, T. Rhodes, A. Judd, M. Hickman, L. G. Johnston, A. Renton, N. Bobrova and A. Sarang (2006). "Methods to recruit hard-to-reach groups: Comparing two chain referral sampling methods of recruiting injecting drug users across nine studies in Russia and Estonia." Journal of Urban Health 83(1 SUPPL.): i39-i53.
20. Robinson, W. T., J. M. H. Risser, S. McGoy, A. B. Becker, H. Rehman, M. Jefferson, V. Griffin, M. Wolverton and S. Tortu (2006). "Recruiting injection drug users: A three-site comparison of results and experiences with respondent-driven and targeted sampling procedures." Journal of Urban Health 83(1 SUPPL.): i29-i38.
21. Siegal, H. A., P. J. Draus, R. G. Carlson, R. S. Falck and J. Wang (2006). "Perspectives on health among adult users of illicit stimulant drugs in rural Ohio." Journal of Rural Health 22(2): 169-173.
22. Simic, M., L. G. Johnston, L. Platt, S. Baros, V. Andjelkovic, T. Novotny and T. Rhodes (2006). "Exploring barriers to 'respondent driven sampling' in sex worker and drug-injecting sex worker populations in Eastern Europe." Journal of Urban Health 83(1 SUPPL.): i6-i15.
23. Steele, M. S., E. Bukusi, C. R. Cohen, B. A. Shell-Duncan and K. K. Holmes (2006). "The ABCs of HIV prevention in men: Associations with HIV risk and protective behaviors." Journal of Acquired Immune Deficiency Syndromes 43(5): 571-576.
24. Stormer, A., W. Tun, L. Guli, A. Harxhi, Z. Bodanovskaia, A. Yakovleva, M. Rusakova, O. Levina, R. Bani, K. Rjepaj and S. Bino (2006). "An analysis of respondent driven sampling with injection drug users (IDU) in Albania and the Russian Federation." Journal of Urban Health 83(1 SUPPL.): i73-i82.
25. Witteveen, E., E. Van Ameijden and G. Schippers (2006). "Motives for and against injecting drug use among young adults in Amsterdam: Qualitative findings and considerations for disease prevention." Substance Use and Misuse 41(6-7): 1001-1016.
26. Yeka, W., G. Maibani-Michie, D. Prybylski and D. Colby (2006). "Application of respondent driven sampling to collect baseline data on FSWs and MSM for HIV risk reduction interventions in two urban centres in Papua New Guinea." Journal of Urban Health 83(1 SUPPL.): i60-i72.
27. Bailey, S. L., L. J. Ouellet, M. E. Mackesy-Amiti, E. T. Golub, H. Hagan, S. M. Hudson, M. H. Latka, W. Gao and R. S. Garfein (2007). "Perceived risk, peer influences, and injection partner type predict receptive syringe sharing among young adult injection drug users in five U.S. cities." Drug and Alcohol Dependence 91(SUPPL. 1): S18-S29.
28. Clark, M. A., C. J. Neighbors, M. R. Wasserman, G. F. Armstrong, M. L. Drnach, S. L. Howie and T. L. Hawthorne (2007). "Strategies and cost of recruitment of middle-aged and older unmarried women in a cancer screening study." Cancer Epidemiology Biomarkers and Prevention 16(12): 2605-2614.
29. Des Jarlais, D. C., K. Arasteh, T. Perlis, H. Hagan, A. Abdul-Quader, D. D. Heckathorn, C. McKnight, H. Bramson, C. Nemeth, L. V. Torian and S. R. Friedman (2007). "Convergence of HIV seroprevalence among injecting and non-injecting drug users in New York City." Aids 21(2): 231-235.
30. Des Jarlais, D. C., K. Arasteh, T. Perlis, H. Hagan, D. D. Heckathorn, C. McKnight, H. Bramson and S. R. Friedman (2007). "The transition from injection to non-injection drug use: Long-term outcomes among heroin and cocaine users in New York City." Addiction 102(5): 778-785.
31. Garrity, T. F., C. G. Leukefeld, R. G. Carlson, R. S. Falck, J. Wang and B. M. Booth (2007). "Physical health, illicit drug use, and demographic characteristics in rural stimulant users." Journal of Rural Health 23(2): 99-107.
32. He, N., F. Y. Wong, Z. J. Huang, Y. Ding, C. Fu, B. D. Smith, D. Young and Q. Jiang (2007). "HIV risks among two types of male migrants in Shanghai, China: Money boys vs. general male migrants." Aids 21(SUPPL. 8): S73-S79.
33. Lansky, A., A. S. Abdul-Quader, M. Cribbin, T. Hall, T. J. Finlayson, R. S. Garfein, L. S. Lin and P. S. Sullivan (2007). "Developing an HIV behavioral surveillance system for injecting drug users: The national HIV behavioral surveillance system." Public Health Reports 122(SUPPL. 1): 48-55.
34. Ma, X., Q. Zhang, X. He, W. Sun, H. Yue, S. Chen, H. F. Raymond, Y. Li, M. Xu, H. Du and W. McFarland (2007). "Trends in prevalence of HIV, syphilis, hepatitis C, hepatitis B, and sexual risk behavior among men who have sex with men: Results of 3 consecutive respondent-driven sampling surveys in Beijing, 2004 through 2006." Journal of Acquired Immune Deficiency Syndromes 45(5): 581-587.
35. Ma, X. Y., Q. Y. Zhang, X. He, J. K. Zhao, Y. Li, W. D. Sun, M. Xu, Q. Zhang and M. F. Willi (2007). "Epidemiological study on the status of HIV/STDs and relative behaviors among MSM in Beijing. [Chinese]." Zhonghua liu xing bing xue za zhi = Zhonghua liuxingbingxue zazhi 28(9): 851-855.
36. Millett, G. A., H. Ding, J. Lauby, S. Flores, A. Stueve, T. Bingham, A. Carballo-Dieguez, C. Murrill, K. L. Liu, D. Wheeler, A. Liau and G. Marks (2007). "Circumcision status and HIV infection among black and Latino men who have sex with men in 3 US cities." Journal of Acquired Immune Deficiency Syndromes 46(5): 643-650.
37. Mimiaga, M. J., H. Goldhammer, C. Belanoff, A. M. Tetu and K. H. Mayer (2007). "Men who have sex with men: Perceptions about sexual risk, HIV and sexually transmitted disease testing, and provider communication." Sexually Transmitted Diseases 34(2): 113-119.
38. Salazar, L. F., R. A. Crosby, D. R. Holtgrave, S. Head, B. Hadsock, J. Todd and R. L. Shouse (2007). "Homelessness and HIV-associated risk behavior among African American men who inject drugs and reside in the urban south of the United States." AIDS and Behavior 11(SUPPL. 2): S70-S77.
39. Soto, R. J., A. E. Ghee, C. A. Nunez, R. Mayorga, K. A. Tapia, S. G. Astete, J. P. Hughes, A. L. Buffardi, S. E. Holte and K. K. Holmes (2007). "Sentinel surveillance of sexually transmitted infections/HIV and risk behaviors in vulnerable populations in 5 Central American countries." JAIDS Journal of Acquired Immune Deficiency Syndromes 46(1): 101-111.
40. Wang, J., R. S. Falck, L. Li, A. Rahman and R. G. Carlson (2007). "Respondent-driven sampling in the recruitment of illicit stimulant drug users in a rural setting: Findings and technical issues." Addictive Behaviors 32(5): 924-937.
41. Wattana, W., F. van Griensven, O. Rhucharoenpornpanich, C. Manopaiboon, W. Thienkrua, R. Bannatham, K. Fox, P. A. Mock, J. W. Tappero and W. C. Levine (2007). "Respondent-driven sampling to assess characteristics and estimate the number of injection drug users in Bangkok, Thailand." Drug and Alcohol Dependence 90(2-3): 228-233.
42. White, E. F., R. S. Garfein, K. C. Brouwer, R. Lozada, R. Ramos, M. Firestone-Cruz, S. G. Perez, C. Magis-Rodriguez, C. J. Conde-Glez and S. A. Strathdee (2007). "Prevalence of hepatitis C virus and HIV infection among injection drug users in two Mexican cities bordering the U.S." Salud Publica de Mexico 49(3): 165-172.
43. Witteveen, E., E. J. C. Van Ameijden, M. Prins and G. M. Schippers (2007). "Unmet needs and barriers to health-care utilization among young adult, problematic drug users: An exploratory study." Sucht 53(3): 169-176.
44. Witteveen, E., E. J. C. Van Ameijden, M. Prins and G. M. Schippers (2007). "Factors associated with the initiation of cocaine and heroin among problem drug users - Reflections on interventions." Substance Use and Misuse 42(6): 933-947.
45. Baumbach, J. P., L. N. Foster, M. Mueller, M. Firestone Cruz, S. Arbona, S. Melville, R. Ramos and S. A. Strathdee (2008). "Seroprevalence of select bloodborne pathogens and associated risk behaviors among injection drug users in the Paso del Norte region of the United States - Mexico border." Harm Reduction Journal 5(33).
46. Becona, E., M. Juan, A. Calafat and M. Ros (2008). "Reasons for not accepting a sexual liaison in nightlife recreational contexts according to gender and state of drunkenness. [Spanish] Razones para no aceptar una relacion sexual en jovenes que se divierten en contextos recreativos nocturnos en funcion del genero y la embriaguez." Adicciones 20(4): 357-364.
47. Bellis, M. A., K. Hughes, A. Calafat, M. Juan, A. Ramon, J. A. Rodriguez, F. Mendes, S. Schnitzer and P. Phillips-Howard (2008). "Sexual uses of alcohol and drugs and the associated health risks: a cross sectional study of young people in nine European cities." BMC Public Health 8(155).
48. Blankenship, K. M., B. S. West, T. S. Kershaw and M. R. Biradavolu (2008). "Power, community mobilization, and condom use practices among female sex workers in Andhra Pradesh, India." Aids 22(SUPPL. 5): S109-S116.
49. Borders, T. F., B. M. Booth, X. Han, P. Wright, C. Leukefeld, R. S. Falck and R. G. Carlson (2008). "Longitudinal changes in methamphetamine and cocaine use in untreated rural stimulant users: Racial differences and the impact of methamphetamine legislation." Addiction 103(5): 800-808.
50. Calafat Far, A., D. Adrover Roig, M. J. Jerez and N. T. Blay Franzke (2008). "Relationship between alcohol, drug use and traffic accidents related to nightlife among a Spanish youth sample in three autonomous communities in 2007. [Spanish] Relacion del consumo de alcohol y drogas de los jovenes Espanoles con la siniestralidad vial durante la vida recreativa nocturna en tres comunidades autonomas en 2007." Revista Espanola de Salud Publica 82(3): 323-331.
51. Colby, D., T. T. Minh and T. T. Toan (2008). "Down on the farm: homosexual behaviour, HIV risk and HIV prevalence in rural communities in Khanh Hoa province, Vietnam." Sexually Transmitted Infections 84(6): 439-443.
52. Deiss, R. G., K. C. Brouwer, O. Loza, R. M. Lozada, R. Ramos, M. A. F. Cruz, T. L. Patterson, D. D. Heckathorn, S. D. Frost and S. A. Strathdee (2008). "High-risk sexual and drug using behaviors among male injection drug users who have sex with men in 2 Mexico-US border cities." Sexually Transmitted Diseases 35(3): 243-249.
53. Far, A. C., D. A. Roig, M. J. Jerez and N. T. Blay Franzke (2008). "Relationship between alcohol, drug use and traffic accidents related to nightlife among a Spanish youth sample in three autonomous communities in 2007. [Spanish] Relacion del consumo de alcohol y drogas de los jovenes Espanoles con la siniestralidad vial durante la vida recreativa nocturna en tres comunidades autonomas en 2007." Revista Espanola de Salud Publica 82(3): 323-331.
54. He, Q., Y. Wang, Y. Li, Y. Zhang, P. Lin, F. Yang, X. Fu, J. Li, H. F. Raymond, L. Ling and W. McFarland (2008). "Accessing men who have sex with men through long-chain referral recruitment, Guangzhou, China." AIDS and Behavior 12(SUPPL. 1): S93-S96.
55. Jagadish, M., G. K. Medhi, R. S. Paranjape, R. Nandan, K. Anjalee, S. B. Akoijam, D. Bernice, H. K. Das, G. Prabuddhagopal and T. Gay (2008). "Injecting and sexual risk behaviours, sexually transmitted infections and HIV prevalence in injecting drug users in three states in India. (Special Issue: Characterizing the Indian HIV epidemic and assessing large-scale prevention efforts - Avahan.)." Aids 22(Supplement 5): S59-S68.
56. Johnston, L. G., R. Khanam, M. Reza, S. I. Khan, S. Banu, S. Alam Md, M. Rahman and T. Azim (2008). "The effectiveness of respondent driven sampling for recruiting males who have sex with males in Dhaka, Bangladesh." AIDS and Behavior 12(2): 294-304.
57. Kajubi, P., M. R. Kamya, H. F. Raymond, S. Chen, G. W. Rutherford, J. S. Mandel and W. McFarland (2008). "Gay and bisexual men in Kampala, Uganda." AIDS and Behavior 12(3): 492-504.
58. Kalichman, S. C., L. C. Simbayi, A. Cloete, C. Cherry, A. Strebel, M. O. Kalichman, T. Shefer, M. Crawford, M. Thabalala, N. Henda and D. Cain (2008). "HIV/AIDS risk reduction and domestic violence prevention intervention for South African men." International Journal of Men's Health 7(3): 255-273.
59. Kissinger, P., N. Liddon, N. Schmidt, E. Curtin, O. Salinas and A. Narvaez (2008). "HIV/STI risk behaviors among Latino migrant workers in New Orleans post-Hurricane Katrina disaster." Sexually transmitted diseases 35(11): 924-929.
60. Lauby, J. L., G. A. Millett, A. B. LaPollo, L. Bond, C. S. Murrill and G. Marks (2008). "Sexual risk behaviors of HIV-positive, HIV-negative, and serostatus-unknown Black men who have sex with men and women." Archives of Sexual Behavior 37(5): 708-719.
61. Mahanta, J., G. K. Medhi, R. S. Paranjape, N. Roy, A. Kohli, S. Brogen Akoijam, B. Dzuvichu, H. K. Das, P. Goswami and G. Thongamba (2008). "Injecting and sexual risk behaviours, sexually transmitted infections and HIV prevalence in injecting drug users in three states in India." Aids 22(SUPPL. 5): S59-S68.
62. Mantecon, A., M. Juan, A. Calafat, E. Becona and E. Roman (2008). "Respondent-Driven Sampling: A new sampling method to study visible and hidden populations. [Spanish] Respondent-Driven Sampling: Un nuevo metodo de muestreo para el estudio de poblaciones visibles y ocultas." Adicciones 20(2): 161-170.
63. Moyer, L. B., K. C. Brouwer, S. K. Brodine, R. Ramos, R. Lozada, M. F. Cruz, C. Magis-Rodriguez and S. A. Strathdee (2008). "Barriers and missed opportunities to HIV testing among injection drug users in two Mexico-US border cities." Drug and Alcohol Review 27(1): 39-45.
64. Philbin, M., R. A. Pollini, R. Ramos, R. Lozada, K. C. Brouwer, M. E. Ramos, M. Firestone-Cruz, P. Case and S. A. Strathdee (2008). "Shooting gallery attendance among IDUs in Tijuana and Ciudad Juarez, Mexico: Correlates, prevention opportunities, and the role of the environment." AIDS and Behavior 12(4): 552-560.
65. Pollini, R. A., K. C. Brouwer, R. M. Lozada, R. Ramos, M. F. Cruz, C. Magis-Rodriguez, P. Case, S. Burris, M. Pu, S. D. W. Frost, L. A. Palinkas, C. Miller and S. A. Strathdee (2008). "Syringe possession arrests are associated with receptive syringe sharing in two Mexico-US border cities." Addiction 103(1): 101-108.
66. Ramirez-Valles, J., D. Garcia, R. T. Campbell, R. M. Diaz and D. D. Heckathorn (2008). "HIV infection, sexual risk behavior, and substance use among Latino gay and bisexual men and transgender persons." American Journal of Public Health 98(6): 1036-1042.
67. Richards, J. E., J. M. Risser, P. M. Padgett, H. U. Rehman, M. L. Wolverton and R. R. Arafat (2008). "Condom use among high-risk heterosexual women with concurrent sexual partnerships, Houston, Texas, USA." International Journal of STD and AIDS 19(11): 768-771.
68. Ruan, S., H. Yang, Y. Zhu, Y. Ma, J. Li, J. Zhao, W. McFarland and H. F. Raymond (2008). "HIV prevalence and correlates of unprotected anal intercourse among men who have sex with men, Jinan, China." AIDS and Behavior 12(3): 469-475.
69. Strathdee, S. A., R. Lozada, V. D. Ojeda, R. A. Pollini, K. C. Brouwer, A. Vera, W. Cornelius, L. Nguyen, C. Magis-Rodriguez and T. L. Patterson (2008). "Differential effects of migration and deportation on HIV infection among male and female injection drug users in Tijuana, Mexico." PLoS ONE 3(7): e2690.
70. Strathdee, S. A., R. Lozada, R. A. Pollini, K. C. Brouwer, A. Mantsios, D. A. Abramovitz, T. Rhodes, C. A. Latkin, O. Loza, J. Alvelais, C. Magis-Rodriguez and T. L. Patterson (2008). "Individual, social, and environmental influences associated with HIV infection among injection drug users in Tijuana, Mexico." Journal of acquired immune deficiency syndromes (1999) 47(3): 369-376.
71. Tun, W., M. De Mello, A. Pinho, M. Chinaglia and J. Diaz (2008). "Sexual risk behaviours and HIV seroprevalence among male sex workers who have sex with men and non-sex workers in Campinas, Brazil." Sexually Transmitted Infections 84(6): 455-457.
72. Uuskula, A., K. Fischer, R. Raudne, H. Kilgi, R. Krylov, M. Salminen, H. Brummer-Korvenkontio, J. St Lawrence and S. Aral (2008). "A study on HIV and hepatitis C virus among commercial sex workers in Tallinn." Sexually transmitted infections 84(3): 189-191.
73. Uuskula, A., M. Kals, K. Rajaleid, K. Abel, A. Talu, K. Ruutel, L. Platt, T. Rhodes, J. DeHovitz and D. Des Jarlais (2008). "High-prevalence and high-estimated incidence of HIV infection among new injecting drug users in Estonia: Need for large scale prevention programs." Journal of Public Health 30(2): 119-125.
74. Wheeler, D. P., J. L. Lauby, K. L. Liu, L. G. Van Sluytman and C. Murrill (2008). "A comparative analysis of sexual risk characteristics of Black men who have sex with men or with men and women." Archives of Sexual Behavior 37(5): 697-707.
75. Abel-Ollo, K., M. Rahu, K. Rajaleid, A. Talu, K. Ruutel, L. Platt, N. Bobrova, T. Rhodes and A. Uuskula (2009). "Knowledge of HIV serostatus and risk behaviour among injecting drug users in Estonia." AIDS Care - Psychological and Socio-Medical Aspects of AIDS/HIV 21(7): 851-857.
76. Abramovitz, D., E. M. Volz, S. A. Strathdee, T. L. Patterson, A. Vera and S. D. W. Frost (2009). "Using respondent-driven sampling in a hidden population at risk of HIV infection: Who do HIV-positive recruiters recruit?" Sexually Transmitted Diseases 36(12): 750-756.
77. Altaf, A. (2009). "Explosive expansion of HIV and associated risk factors among male and hijra sex workers in Sindh, Pakistan." Journal of Acquired Immune Deficiency Syndromes 51: 158.
78. Bao, Y. G., Y. H. Zhang, J. K. Zhao, J. P. Sun and H. Z. Tan (2009). "HIV infection and KAP status among men who have sex with men in 14 Chinese cities. [Chinese]." Zhonghua yu fang yi xue za zhi [Chinese journal of preventive medicine] 43(11): 981-983.
79. Bjorkhaug, I. and A. Hatloy (2009). "Utilization of respondent-driven sampling among a population of child workers in the diamond-mining sector of Sierra Leone." Global Public Health 4(1): 96-109.
80. Bobashev, G. V., W. A. Zule, K. C. Osilla, T. L. Kline and W. M. Wechsberg (2009). "Transactional sex among men and women in the south at high risk for hiv and other STIs." Journal of Urban Health 86(SUPPL. 1): S32-S47.
81. Booth, B., C. Leukefeld, R. Falck and R. Carlson (2009). "Health correlates of long-term substance use and increasing age." American Journal of Geriatric Psychiatry 17: A100-A101.
82. Bozicevic, I., O. D. Rode, S. Z. Lepej, L. G. Johnston, A. Stulhofer, Z. Dominkovic, V. Bacak, D. Lukas and J. Begovac (2009). "Prevalence of sexually transmitted infections among men who have sex with men in Zagreb, Croatia." AIDS and Behavior 13(2): 303-309.
83. Brouwer, K. C., R. Lozada, W. A. Cornelius, M. Firestone Cruz, C. Magis-Rodriguez, M. L. Zuniga de Nuncio and S. A. Strathdee (2009). "Deportation along the U.S.-Mexico border: Its relation to drug use patterns and accessing care." Journal of Immigrant and Minority Health 11(1): 1-6.
84. Burt, R. D., H. Thiede and H. Hagan (2009). "Serosorting for hepatitis C status in the sharing of injection equipment among Seattle area injection drug users." Drug and Alcohol Dependence 105(3): 215-220.
85. Calafat, A., N. Blay, M. Juan, D. Adrover, M. A. Bellis, K. Hughes, P. Stocco, I. Siamou, F. Mendes and K. Bohrn (2009). "Traffic risk behaviors at nightlife: drinking, taking drugs, driving, and use of public transport by young people." Traffic injury prevention 10(2): 162-169.
86. Calafat, A., M. Juan, E. Becona, A. Mantecon and A. Ramon (2009). "Risky sexual behaviour and drug use in recreational nightlife context. A gender perspective. [Spanish] Sexualidad de riesgo y consumo de drogas en el contexto recreativo. Una perspectiva de genero." Psicothema 21(2): 227-233.
87. Chopra, M., L. Townsend, L. Johnston, C. Mathews, M. Tomlinson, H. O'Bra and C. Kendall (2009). "Estimating hiv prevalence and risk behaviors among high-risk heterosexual men with multiple sex partners: Use of respondent-driven sampling." Journal of Acquired Immune Deficiency Syndromes 51(1): 72-77.
88. Clark, M. A., M. L. Rogers, G. F. Armstrong, W. Rakowski, D. J. Bowen, T. Hughes and K. A. McGarry (2009). "Comprehensive cancer screening among unmarried women aged 40-75 years: Results from the cancer screening project for women." Journal of Women's Health 18(4): 451-459.
89. Daniulaityte, R., R. S. Falck, J. Wang and R. G. Carlson (2009). "Illicit use of pharmaceutical opioids among young polydrug users in Ohio." Addictive Behaviors 34(8): 649-653.
90. DeJong, J., Z. Mahfoud, D. Khoury, F. Barbir and R. A. Afifi (2009). "Ethical considerations in HIV/AIDS biobehavioral surveys that use respondent-driven sampling: Illustrations from Lebanon." American Journal of Public Health 99(9): 1562-1567.
91. Farooq, M. U. (2009). "HIV and sexual practices of transgender community in Pakistan." Tropical Medicine and International Health 14: 68.
92. Feng, T., H. Liu, H. Feng, Y. Cai, A. G. Rhodes and O. Grusky (2009). "Egocentric networks of Chinese men who have sex with men: Network components, condom use norms, and safer sex." AIDS Patient Care and STDs 23(10): 885-893.
93. Feng, T., A. G. Rhodes and H. Liu (2009). "Assessment of the Chinese version of HIV and homosexuality related stigma scales." Sexually Transmitted Infections 85(1): 65-69.
94. Garfein, R. S., R. Lozada, L. Liu, R. Laniado-Laborin, T. C. Rodwell, R. Deiss, J. Alvelais, A. Catanzaro, P. G. Chiles and S. A. Strathdee (2009). "High prevalence of latent tuberculosis infection among injection drug users in Tijuana, Mexico." International Journal of Tuberculosis and Lung Disease 13(5): 626-632.
95. Gondim, R. C., L. R. F. S. Kerr, G. L. Werneck, R. H. M. Macena, M. K. Pontes and C. Kendall (2009). "Risky sexual practices among men who have sex with men in Northeast Brazil: Results from four sequential surveys. [Portuguese] Praticas sexuais de risco de homens que fazem sexo com homens no nordeste do brasil: Resultados de quatro inqueritos sequenciais." Cadernos de Saude Publica 25(6): 1390-1398.
96. Gorbach, P. M., R. Murphy, R. E. Weiss, C. Hucks-Ortiz and S. Shoptaw (2009). "Bridging sexual boundaries: Men who have sex with men and women in a street-based sample in Los Angeles." Journal of Urban Health 86(SUPPL. 1): S63-S76.
97. Hawkes, S., M. Collumbien, L. Platt, N. Lalji, N. Rizvi, A. Andreasen, J. Chow, R. Muzaffar, H. ur-Rehman, N. Siddiqui, S. Hasan and A. Bokhari (2009). "HIV and other sexually transmitted infections among men, transgenders and women selling sex in two cities in Pakistan: a cross-sectional prevalence survey." Sexually transmitted infections 85 Suppl 2: ii8-16.
98. Hequembourg, A. L. and R. L. Dearing (2009). "Preliminary findings regarding shame and guilt among sexual minorities." Alcoholism: Clinical and Experimental Research 33: 125A.
99. Hickman, M., V. Hope, B. Coleman, J. Parry, M. Telfer, J. Twigger, C. Irish, J. Macleod and H. Annett (2009). "Assessing IDU prevalence and health consequences (HCV, overdose and drug-related mortality) in a primary care trust: implications for public health action." Journal of public health (Oxford, England) 31(3): 374-382.
100. Iguchi, M. Y., A. J. Ober, S. H. Berry, T. Fain, D. D. Heckathorn, P. M. Gorbach, R. Heimer, A. Kozlov, L. J. Ouellet, S. Shoptaw and W. A. Zule (2009). "Simultaneous recruitment of drug users and men who have sex with men in the united states and Russia using respondent-driven sampling: Sampling methods and implications." Journal of Urban Health 86(SUPPL. 1): S5-S31.
101. Jenness, S. M., C. S. Murrill, K. L. Liu, T. Wendel, E. Begier and H. Hagan (2009). "Missed opportunities for HIV testing among high-risk heterosexuals." Sexually Transmitted Diseases 36(11): 704-710.
102. Johnson, C. V., M. J. Mimiaga, S. L. Reisner, A. M. Tetu, K. Cranston, T. Bertrand, D. S. Novak and K. H. Mayer (2009). "Health care access and sexually transmitted infection screening frequency among at-risk Massachusetts men who have sex with men." American journal of public health 99 Suppl 1: S187-192.
103. Johnston, L. G., A. Trummal, L. Lohmus and A. Ravalepik (2009). "Efficacy of convenience sampling through the internet versus respondent driven sampling among males who have sex with males in Tallinn and Harju County, Estonia: Challenges reaching a hidden population." AIDS Care - Psychological and Socio-Medical Aspects of AIDS/HIV 21(9): 1195-1202.
104. Judd, A., T. Rhodes, L. G. Johnston, L. Platt, V. Andjelkovic, D. Simic, B. Mugosa, M. Simic, S. Zerjav, R. P. Parry and J. V. Parry (2009). "Improving survey methods in sero-epidemiological studies of injecting drug users: a case example of two cross sectional surveys in Serbia and Montenegro." BMC Infectious Diseases 9(14).
105. King, W. D., S. Larkins, C. Hucks-Ortiz, P. C. Wang, P. M. Gorbach, R. Veniegas and S. Shoptaw (2009). "Factors associated with HIV viral load in a respondent driven sample in Los Angeles." AIDS and Behavior 13(1): 145-153.
106. Lepej, S. Z., I. B. Vrakela, M. Poljak, I. Bozicevic and J. Begovac (2009). "Phylogenetic analysis of HIV sequences obtained in a respondent-driven sampling study of men who have sex with men." AIDS Research and Human Retroviruses 25(12): 1335-1338.
107. Li, Y., P. Lin, R. Detels, X. Fu, Z. Deng, Y. Liu, J. Li, Y. Tan and G. Huang (2009). "Prevalence of HIV infection and sexually transmitted diseases and associated risk factors among female sex workers in Guangdong province. [Chinese]." Disease Surveillance 24(8): 599-602.
108. Lin, P., J. K. Zhao, Y. Li, M. Wang and Q. L. Zhang (2009). "[Study on cognition and behavior of methadone maintenance treatment among the community-based drug users in Dongguan, Guangdong province]. [Chinese]." Zhonghua liu xing bing xue za zhi = Zhonghua liuxingbingxue zazhi 30(12): 1234-1237.
109. Liu, H., Y. Cai, A. G. Rhodes and F. Hong (2009). "Money boys, HIV risks, and the associations between norms and safer sex: A respondent-driven sampling study in Shenzhen, China." AIDS and Behavior 13(4): 652-662.
110. Liu, H., T. Feng, A. G. Rhodes and H. Liu (2009). "Assessment of the Chinese version of HIV and homosexuality related stigma scales." Sexually Transmitted Infections 85(1): 65-69.
111. Liu, H. J., T. Feng, H. Liu, H. Feng, Y. Cai, A. G. Rhodes and O. Grusky (2009). "Egocentric networks of Chinese men who have sex with men: network components, condom use norms, and safer sex." AIDS Patient Care and STDs 23(10): 885-893.
112. Lomba, L., J. Apostolo and F. Mendes (2009). "Drugs and alcohol consumption and sexual behaviours in night recreational settings in Portugal." Adicciones 21(4): 309-325.
113. Magis-Rodriguez, C., G. Lemp, M. T. Hernandez, M. A. Sanchez, F. Estrada and E. Bravo-Garcia (2009). "Going North: Mexican migrants and their vulnerability to HIV." Journal of acquired immune deficiency syndromes (1999) 51 Suppl 1: S21-25.
114. Magnus, M., I. Kuo, K. Shelley, A. Rawls, J. Peterson, L. Montanez, T. West-Ojo, S. Hader, F. Hamilton and A. E. Greenberg (2009). "Risk factors driving the emergence of a generalized heterosexual HIV epidemic in Washington, District of Columbia networks at risk." Aids 23(10): 1277-1284.
115. Maryam, S., W. Sonali, F. Cowan, D. Mabey, A. Copas and P. Vikram (2009). "Suicidal behavior among female sex workers in Goa, India: the silent epidemic." American Journal of Public Health 99(7): 1239-1246.
116. Mimiaga, M. J., P. Case, C. V. Johnson, S. A. Safren and K. H. Mayer (2009). "Preexposure antiretroviral prophylaxis attitudes in high-risk Boston area men who report having sex with men: limited knowledge and experience but potential for increased utilization after education." Journal of acquired immune deficiency syndromes (1999) 50(1): 77-83.
117. Mimiaga, M. J., S. L. Reisner, S. Bland, M. Skeer, K. Cranston, D. Isenberg, B. A. Vega and K. H. Mayer (2009). "Health system and personal barriers resulting in decreased utilization of HIV and STD testing services among at-risk black men who have sex with men in Massachusetts." AIDS Patient Care and STDs 23(10): 825-835.
118. Mimiaga, M. J., S. L. Reisner, K. Cranston, D. Isenberg, D. Bright, G. Daffin, S. Bland, M. A. Driscoll, R. Vanderwarker, B. Vega and K. H. Mayer (2009). "Sexual mixing patterns and partner characteristics of black msm in massachusetts at increased risk for HIV infection and transmission." Journal of Urban Health 86(4): 602-623.
119. Mimiaga, M. J., S. L. Reisner, A. M. Tetu, K. E. Bonafide, K. Cranston, T. Bertrand, D. S. Novak and K. H. Mayer (2009). "Partner notification after STD and HIV exposures and infections: Knowledge, attitudes, and experiences of Massachusetts men who have sex with men." Public Health Reports 124(1): 111-119.
120. Ober, A., S. Shoptaw, P. C. Wang, P. Gorbach and R. E. Weiss (2009). "Factors associated with event-level stimulant use during sex in a sample of older, low-income men who have sex with men in Los Angeles." Drug and Alcohol Dependence 102(1-3): 123-129.
121. Ouellet, L. J., Y. Youm, M. E. MacKesy-Amiti and C. T. Williams (2009). "Identifying hidden sexual bridging communities in chicago." Journal of Urban Health 86(SUPPL. 1): S107-S120.
122. Ouyang, L., L. G. Feng, X. B. Ding, J. K. Zhao, J. Xu, M. Han and C. Zhou (2009). "A respondent-driven sampling survey on HIV and risk factors among men who have sex with men in Chongqing. [Chinese]." Zhonghua liu xing bing xue za zhi = Zhonghua liuxingbingxue zazhi 30(10): 1001-1004.
123. Paintsil, E., S. V. Verevochkin, E. Dukhovlinova, L. Niccolai, R. Barbour, E. White, O. V. Toussova, L. Alexander, A. P. Kozlov and R. Heimer (2009). "Hepatitis C virus infection among drug injectors in St Petersburg, Russia: Social and molecular epidemiology of an endemic infection." Addiction 104(11): 1881-1890.
124. Pollini, R. A., J. Alvelais, M. Gallardo, A. Vera, R. Lozada, C. Magis-Rodriquez and S. A. Strathdee (2009). "The harm inside: Injection during incarceration among male injection drug users in Tijuana, Mexico." Drug and Alcohol Dependence 103(1-2): 52-58.
125. Qiu, P., Y. Yang and P. Yuan (2009). "Implication of respondent-driven sampling in epidemiological research. [Chinese]." Modern Preventive Medicine 36(23): 4401-4403.
126. Raymond, H. F., P. Kajubi, M. R. Kamya, G. W. Rutherford, J. S. Mandel and W. McFarland (2009). "Correlates of unprotected receptive anal intercourse among gay and bisexual men: Kampala, Uganda." AIDS and Behavior 13(4): 677-681.
127. Rehan, N., I. Chaudhary and S. K. Shah (2009). "Socio-sexual behaviour of Hijras of Lahore." Journal of the Pakistan Medical Association 59(6): 380-384.
128. Reisner, S. L., M. J. Mimiaga, P. Case, C. V. Johnson, S. A. Safren and K. H. Mayer (2009). "Predictors of Identifying as a Barebacker among High-Risk New England HIV Seronegative Men Who Have Sex with Men." Journal of Urban Health 86(2): 250-262.
129. Reisner, S. L., M. J. Mimiaga, S. A. Safren and K. H. Mayer (2009). "Stressful or traumatic life events, post-traumatic stress disorder (PTSD) symptoms, and HIV sexual risk taking among men who have sex with men." AIDS Care - Psychological and Socio-Medical Aspects of AIDS/HIV 21(12): 1481-1489.
130. Reisner, S. L., M. J. Mimiaga, M. Skeer, D. Bright, K. Cranston, D. Isenberg, S. Bland, T. A. Barker and K. H. Mayer (2009). "Clinically significant depressive symptoms as a risk factor for HIV infection among black MSM in Massachusetts." AIDS and Behavior 13(4): 798-810.
131. Risser, J. M. H., P. Padgett, M. Wolverton and W. L. Risser (2009). "Relationship between heterosexual anal sex, injection drug use and HIV infection among black men and women." International Journal of STD and AIDS 20(5): 310-314.
132. Ruan, S., H. Yang, Y. Zhu, M. Wang, Y. Ma, J. Zhao, W. McFarland and H. F. Raymond (2009). "Rising HIV prevalence among married and unmarried among men who have sex with men: Jinan, China." AIDS and Behavior 13(4): 671-676.
133. Ruan, S., H. Yang, Y. Zhu, J. Zhao, M. Wang, C. Zhang, L. Xu and Z. Jia (2009). "Application of respondent driven sampling in the study on male homosexual population in Jinan city. [Chinese]." Disease Surveillance 24(6): 416-418.
134. Rusch, M. L., R. Lozada, R. A. Pollini, A. Vera, T. L. Patterson, P. Case and S. A. Strathdee (2009). "Polydrug use among IDUs in Tijuana, Mexico: Correlates of methamphetamine use and route of administration by gender." Journal of Urban Health 86(5): 760-775.
135. Shahmanesh, M., F. Cowan, S. Wayal, A. Copas, V. Patel and D. Mabey (2009). "The burden and determinants of HIV and sexually transmitted infections in a population-based sample of female sex workers in Goa, India." Sexually Transmitted Infections 85(1): 50-59.
136. Shahmanesh, M., S. Wayal, A. Copas, V. Patel, D. Mabey and F. Cowan (2009). "A study comparing sexually transmitted infections and hiv among ex-red-light district and non-red-light district sex workers after the demolition of baina red-light district." Journal of Acquired Immune Deficiency Syndromes 52(2): 253-257.
137. Shahmanesh, M., S. Wayal, F. Cowan, D. Mabey, A. Copas and V. Patel (2009). "Suicidal behavior among female sex workers in Goa, India: the silent epidemic." American journal of public health 99(7): 1239-1246.
138. Sharra, E. and R. Bani (2009). "An analysis of HIV-related risk behaviors of men having sex with men (MSM), using respondent driven sampling (RDS), in Albania." Archives The International Journal of Medicine 2(2): 231-234.
139. Shoptaw, S., R. E. Weiss, B. Munjas, C. Hucks-Ortiz, S. D. Young, S. Larkins, G. D. Victorianne and P. M. Gorbach (2009). "Homonegativity, substance use, sexual risk behaviors, and hiv status in poor and ethnic men who have sex with men in Los Angeles." Journal of Urban Health 86(SUPPL. 1): S77-S92.
140. Verevochkin, S. (2009). "High incidence cohort of IDUs infected with HIV with low genetics diversity for HIV vaccine efficacy trials." Journal of Acquired Immune Deficiency Syndromes 51: 167.
141. Vorobjov, S., A. Uuskula, K. Abel-Ollo, A. Talu, K. Ruutel and D. C. Des Jarlais (2009). "Comparison of injecting drug users who obtain syringes from pharmacies and syringe exchange programs in Tallinn, Estonia." Harm Reduction Journal 6(3).
142. Wang, M., P. Lin, J. Zhao, Y. Li and Q. Zhang (2009). "Correlation analysis of drug abuse, sexual behaviours and other risk factors in the community-based drug addicts. [Chinese]." Journal of Tropical Medicine 9(11): 1291-1294.
143. Wang, M., P. Lin, J. K. Zhao, Y. Li and Q. L. Zhang (2009). "HIV prevalence and its correlation among the community-based drug users in Dongguan in 2008. [Chinese]." Zhonghua yu fang yi xue za zhi [Chinese journal of preventive medicine] 43(11): 1004-1008.
144. Williams, C. T., M. E. MacKesy-Amiti, D. J. McKirnan and L. J. Ouellet (2009). "Differences in sexual identity, risk practices, and sex partners between bisexual men and other men among a low-income drug-using sample." Journal of Urban Health 86(SUPPL. 1): S93-S106.
145. Yang, H., S. Ruan and Y. Zhu (2009). "Analysis of syphilis and HIV infection situation among men who have sex with men in Jinan city. [Chinese]." Chinese Preventive Medicine 10(8): 740-742.
146. Youm, Y., M. E. Mackesy-Amiti, C. T. Williams and L. J. Ouellet (2009). "Identifying hidden sexual bridging communities in Chicago. (Special Issue: Sexual Acquisition and Transmission of HIV Cooperative Agreement Program (SATHCAP).)." Journal of Urban Health: Bulletin of the New York Academy of Medicine 86(Suppl. 1): 107-120.
147. Abdelrahim, M. S. (2010). "HIV prevalence and risk behaviors of female sex workers in Khartoum, north Sudan." Aids 24(SUPPL. 2): S55-S60.
148. Ahmed, H. (2010). "Sexual practices of Transgender community in Pakistan." AIDS Research and Human Retroviruses 26 (10): A96.
149. Bauer, G., M. Boyce, T. Coleman, N. Khobzi, R. Travers, J. Pyne and K. Scanlon (2010). "Lessons learned from respondent-driven sampling implementation: Trans pulse project." Canadian Journal of Infectious Diseases and Medical Microbiology SB): 28B-29B.
150. Bauer, G., T. Coleman, R. Travers, R. Hammond, K. Anjali, M. Kaay, N. Redman, K. Tokawa and A. Travers (2010). "HIV-related behavioural risk in ontario's trans communities: Trans pulse project." Canadian Journal of Infectious Diseases and Medical Microbiology SB): 77B.
151. Bhat, S., Jayaprakash, M. M. Shenoy, M. Vinay and M. Bhavanishankar (2010). "A study on mental health of sex workers." Indian Journal of Psychiatry 52: S43.
152. Blankenship, K. M., R. Burroway and E. Reed (2010). "Factors associated with awareness and utilisation of a community mobilisation intervention for female sex workers in Andhra Pradesh, India." Sexually Transmitted Infections 86(SUPPL. 1): i69-i75.
153. Blay, N., A. Calafat, M. Juan, E. Becona, A. Mantecon, M. Ros and A. Far (2010). "Violence in nightlife environments and its relationship with the consumption of alcohol and drugs among young Spaniards. [Spanish] Violencia en contextos recreativos nocturnos: Su relacion con el consumo de alcohol y drogas entre jovenes Espanoles." Psicothema 22(3): 396-402.
154. Boodram, B., E. T. Golub and L. J. Ouellet (2010). "Socio-behavioral and geographic correlates of prevalent hepatitis C virus infection among young injection drug users in metropolitan Baltimore and Chicago." Drug and Alcohol Dependence 111(1-2): 136-145.
155. Booth, B. M., G. Curran, X. Han, P. Wright, S. Frith, C. Leukefeld, R. Falck and R. G. Carlson (2010). "Longitudinal relationship between psychological distress and multiple substance use: Results from a three-year multisite natural-history study of rural stimulant users." Journal of Studies on Alcohol and Drugs 71(2): 258-267.
156. Burt, R. D., H. Hagan, K. Sabin and H. Thiede (2010). "Evaluating Respondent-Driven Sampling in a Major Metropolitan Area: Comparing Injection Drug Users in the 2005 Seattle Area National HIV Behavioral Surveillance System Survey with Participants in the RAVEN and Kiwi Studies." Annals of Epidemiology 20(2): 159-167.
157. Busari, O. A., M. Nakayima, O. Busari and D. Oyerinde (2010). "Preventive HIV vaccine acceptability and behavioural risk compensation among men who have sex with men in Nigeria, sub-Saharan Africa." AIDS Research and Human Retroviruses 26 (10): A18.
158. Busari, O. A., M. Nakayima and D. Oyerinde (2010). "HIV and STI prevalence among men who have sex with men recruited through respondent driven sample in Lagos, sub-Saharan Africa." AIDS Research and Human Retroviruses 26 (10): A98-A99.
159. Cao, X., L. Yu and H. Zhou (2010). "Smoking and its impact factors among migrant workers in Chengdu. [Chinese]." Modern Preventive Medicine 37(15): 2860-2862.
160. Carlson, R. G., R. Sexton, J. Wang, R. Falck, C. G. Leukefeld and B. M. Booth (2010). "Predictors of substance abuse treatment entry among rural illicit stimulant users in Ohio, Arkansas, and Kentucky." Substance abuse : official publication of the Association for Medical Education and Research in Substance Abuse 31(1): 1-7.
161. Doocy, S., S. Malik and G. Burnham (2010). "Experiences of Iraqi doctors in Jordan during conflict and factors associated with migration." American journal of disaster medicine 5(1): 41-47.
162. Gwadz, M. V., C. M. Cleland, R. Quiles, D. Nish, J. Welch, L. S. Michaels, J. L. Gonzalez, A. S. Ritchie and N. R. Leonard (2010). "CDC HIV testing guidelines and the rapid and conventional testing practices of homeless youth." AIDS Education and Prevention 22(4): 312-327.
163. Ha, T. H., H. Liu, Y. Cai and T. Feng (2010). "Concurrent sexual partnerships among men who have sex with men in Shenzhen, China." Sexually Transmitted Diseases 37(8): 506-511.
164. Hagan, H., S. M. Jenness, T. Wendel, C. R. Murrill, A. Neaigus and C. Gelpi-Acosta (2010). "Herpes simplex virus type 2 associated with HIV infection among New York heterosexuals living in high-risk areas." International Journal of STD and AIDS 21(8): 580-583.
165. Han, D. L., Z. Liu, X. Y. Ma, L. L. Wang, J. Xu, L. Pang, H. B. Zhang and Z. Y. Wu (2010). "[The prevalence of HIV infection and the risk factors among MSM in 4 cities, China]. [Chinese]." Zhonghua yu fang yi xue za zhi [Chinese journal of preventive medicine] 44(11): 975-980.
166. Hathaway, A. D., E. Hyshka, P. G. Erickson, M. Asbridge, S. Brochu, M. Cousineau, C. Duff and D. Marsh (2010). "Whither RDS? An investigation of Respondent Driven Sampling as a method of recruiting mainstream marijuana users." Harm Reduction Journal 7(15).
167. Hequembourg, A., J. Livingston and J. Jefferson (2010). "Adult sexual victimization among sexual minority men and women: The role of alcohol." Alcoholism: Clinical and Experimental Research 34 (6): 126A.
168. Iskandar, S., D. Basar, T. Hidayat, I. M. Siregar, L. Pinxten, R. van Crevel, A. J. Van der Ven and C. A. De Jong (2010). "High risk behavior for HIV transmission among former injecting drug users: a survey from Indonesia." BMC public health 10: 472.
169. Jenness, S. M., A. Neaigus, H. Hagan, C. S. Murrill and T. Wendel (2010). "Heterosexual HIV and sexual partnerships between injection drug users and noninjection drug users." AIDS Patient Care and STDs 24(3): 175-181.
170. Johnston, L., H. O'Bra, M. Chopra, C. Mathews, L. Townsend, K. Sabin, M. Tomlinson and C. Kendall (2010). "The associations of voluntary counseling and testing acceptance and the perceived likelihood of being HIV-infected among men with multiple sex partners in a South African township." AIDS and behavior 14(4): 922-931.
171. Johnston, L. G., A. Holman, M. Dahoma, L. A. Miller, E. Kim, M. Mussa, A. A. Othman, A. Kim, C. Kendall and K. Sabin (2010). "HIV risk and the overlap of injecting drug use and high-risk sexual behaviours among men who have sex with men in Zanzibar (Unguja), Tanzania." International Journal of Drug Policy 21(6): 485-492.
172. Johnston, L. G., T. R. Thurman, N. Mock, L. Nano and V. Carcani (2010). "Respondent-driven sampling: A new method for studying street children with findings from Albania." Vulnerable Children and Youth Studies 5(1): 1-11.
173. Johnston, L. G., S. Whitehead, M. Simic-Lawson and C. Kendall (2010). "Formative research to optimize respondent-driven sampling surveys among hard-to-reach populations in HIV behavioral and biological surveillance: Lessons learned from four case studies." AIDS Care - Psychological and Socio-Medical Aspects of AIDS/HIV 22(6): 784-792.
174. Kogan, S. M., G. H. Brody, Y. F. Chen, C. M. Grange, L. M. Slater and R. J. DiClemente (2010). "Risk and protective factors for unprotected intercourse among rural African American young adults." Public health reports (Washington, D.C: 1974). 125(5): 709-717.
175. Kral, A. H., M. Malekinejad, J. Vaudrey, A. N. Martinez, J. Lorvick, W. McFarland and H. F. Raymond (2010). "Comparing respondent-driven sampling and targeted sampling methods of recruiting injection drug users in San Francisco." Journal of urban health : bulletin of the New York Academy of Medicine 87(5): 839-850.
176. Kriitmaa, K., A. Testa, M. Osman, I. Bozicevic, G. Riedner, J. Malungu, G. Irving and I. Abdalla (2010). "HIV prevalence and characteristics of sex work among female sex workers in Hargeisa, Somaliland, Somalia." Aids 24(SUPPL. 2): S61-S67.
177. Li, L., J. Qi and W. Liu (2010). "Survey on HIV infection status among men who have sex with men and their behavioral feature. [Chinese]." Modern Preventive Medicine 37(13): 2484-2486.
178. Li, Y., R. Detels, P. Lin, X. Fu, Z. Deng, Y. Liu, Y. Tan, J. Li and Z. Wu (2010). "Prevalence of HIV and STIs and associated risk factors among female sex workers in guangdong Province, China." Journal of Acquired Immune Deficiency Syndromes 53(SUPPL. 1): S48-S53.
179. Lomba, L., J. Apostolo and F. Mendes (2010). "Sexual risk behaviours related to alcohol and drug use at night recreational settings." Sexologies 19: S141-S142.
180. Mahfoud, Z., R. Afifi, S. Ramia, D. E. Khoury, K. Kassak, F. E. Barbir, M. Ghanem, M. El-Nakib and J. Dejong (2010). "HIV/AIDS among female sex workers, injecting drug users and men who have sex with men in Lebanon: Results of the first biobehavioral surveys." Aids 24(SUPPL. 2): S45-S54.
181. Mahfoud, Z., K. Kassak, K. Kreidieh, S. Shamra and S. Ramia (2010). "Distribution of hepatitis C virus genotypes among injecting drug users in Lebanon." Virology Journal 7(96).
182. Mimiaga, M. J., S. L. Reisner, Y. M. Fontaine, S. E. Bland, M. A. Driscoll, D. Isenberg, K. Cranston, M. R. Skeer and K. H. Mayer (2010). "Walking the line: Stimulant use during sex and HIV risk behavior among Black urban MSM." Drug and Alcohol Dependence 110(1-2): 30-37.
183. Mimiaga, M. J., S. L. Reisner, H. Goldhammer, A. M. Tetu, C. Belanoff and K. H. Mayer (2010). "Sources of human immunodeficiency virus and sexually transmitted disease information and responses to prevention messages among Massachusetts men who have sex with men." American journal of health promotion : AJHP 24(3): 170-177.
184. Pilon, R., L. E. Leonard, J. Kim, D. Vallee, E. De Rubeis, A. M. Jolly, J. Wylie, L. Pelude and P. Sandstrom (2010). "Transmission patterns of HIV and HCV within ottawa IDU social networks." Canadian Journal of Infectious Diseases and Medical Microbiology SB): 44B.
185. Qiu, P., Y. Yang and Q. Chen (2010). "Depression and its impact factors among migrant workers in Chengdu. [Chinese]." Modern Preventive Medicine 37(22): 4263-4266.
186. Ragnarsson, A., L. Townsend, A. M. Ekstrom, M. Chopra and A. Thorson (2010). "The construction of an idealised urban masculinity among men with concurrent sexual partners in a South African township." Global Health Action 3(5092).
187. Ramia, S., Z. Mahfoud, R. Afifi, J. Dejong, K. Kreidieh, S. Shamra and K. Kassak (2010). "HIV and viral hepatitis (HBV and HCV) among four vulnerable groups in Lebanon." Clinical Microbiology and Infection 16: S335.
188. Ramirez-Valles, J., L. M. Kuhns, R. T. Campbell and R. M. Diaz (2010). "Social integration and health: community involvement, stigmatized identities, and sexual risk in Latino sexual minorities." Journal of health and social behavior 51(1): 30-47.
189. Ramos, R. L., J. B. Ferreira-Pinto, M. Rusch and M. E. Ramos (2010). "Pasa la Voz (spread the word): using women's social networks for HIV education and testing." Public Health Reports 125(4): 528-533.
190. Reed, E., J. Gupta, M. Biradavolu, V. Devireddy and K. M. Blankenship (2010). "The context of economic insecurity and its relation to violence and risk factors for HIV among female sex workers in Andhra Pradesh, India." Public health reports (Washington, D.C: 1974). 125 Suppl 4: 81-89.
191. Reisner, S. L., M. J. Mimiaga, S. Bland, M. Skeer, K. Cranston, D. Isenberg, M. Driscoll and K. H. Mayer (2010). "Problematic alcohol use and HIV risk among black men who have sex with men in massachusetts." AIDS Care - Psychological and Socio-Medical Aspects of AIDS/HIV 22(5): 577-587.
192. Reisner, S. L., M. J. Mimiaga, C. V. Johnson, S. Bland, P. Case, S. A. Safren and K. H. Mayer (2010). "What makes a respondent-driven sampling "seed" productive? Example of finding at-risk Massachusetts men who have sex with men." Journal of urban health : bulletin of the New York Academy of Medicine 87(3): 467-479.
193. Risser, J., A. Cates, H. Rehman and W. Risser (2010). "Gender differences in social support and depression among injection drug users in Houston, Texas." American Journal of Drug and Alcohol Abuse 36(1): 18-24.
194. Robertson, A. M., A. Y. Vera, M. Gallardo, R. A. Pollini, T. L. Patterson, P. Case, L. Nguyen and S. A. Strathdee (2010). "Correlates of seeking injection assistance among injection drug users in Tijuana, Mexico." American Journal on Addictions 19(4): 357-363.
195. Salazar, L. F., R. A. Crosby, S. Head and A. Siegler (2010). "Male injecting drug users in the Deep South: Bisexual behaviour is a marker for elevated HIV risk." International Journal of STD and AIDS 21(10): 691-696.
196. Shah, A. M., L. Guo, M. Magee, W. Cheung, M. Simon, A. LaBreche and H. Liu (2010). "Comparing selected measures of health outcomes and health-seeking behaviors in Chinese, Cambodian, and Vietnamese communities of Chicago: results from local health surveys." Journal of urban health : bulletin of the New York Academy of Medicine 87(5): 813-826.
197. Soliman, C., I. A. Rahman, S. Shawky, T. Bahaa, S. Elkamhawi, A. A. E. Sattar, D. Oraby, D. Khaled, B. Feyisetan, E. Salah, Z. E. Taher and N. E. Sayed (2010). "HIV prevalence and risk behaviors of male injection drug users in Cairo, Egypt." Aids 24(SUPPL. 2): S33-S38.
198. Solomon, S. S., S. H. Mehta, A. Latimore, A. K. Srikrishnan and D. D. Celentano (2010). "The impact of HIV and high-risk behaviours on the wives of married men who have sex with men and injection drug users: implications for HIV prevention." Journal of the International AIDS Society 13 Suppl 2: S7.
199. Solomon, S. S., A. K. Srikrishnan, F. Sifakis, S. H. Mehta, C. K. Vasudevan, P. Balakrishnan, K. H. Mayer, S. Solomon and D. D. Celentano (2010). "The emerging HIV epidemic among men who have sex with men in Tamil Nadu, India: geographic diffusion and bisexual concurrency." AIDS and behavior 14(5): 1001-1010.
200. Syvertsen, J., R. A. Pollini, R. Lozada, A. Vera, G. Rangel and S. A. Strathdee (2010). "Managing la malilla: Exploring drug treatment experiences among injection drug users in Tijuana, Mexico, and their implications for drug law reform." International Journal of Drug Policy 21(6): 459-465.
201. Talu, A., K. Rajaleid, K. Abel-Ollo, K. Ruutel, M. Rahu, T. Rhodes, L. Platt, N. Bobrova and A. Uuskula (2010). "HIV infection and risk behaviour of primary fentanyl and amphetamine injectors in Tallinn, Estonia: Implications for intervention." International Journal of Drug Policy 21(1): 56-63.
202. Tao, X., R. Gai, N. Zhang, W. Zheng, X. Zhang, A. Xu and S. Li (2010). "HIV infection and mental health of "money boys": A pilot study in Shandong Province, China." Southeast Asian Journal of Tropical Medicine and Public Health 41(2): 358-368.
203. Townsend, L., L. G. Johnston, A. J. Flisher, C. Mathews and Y. Zembe (2010). "Effectiveness of respondent-driven sampling to recruit high risk heterosexual men who have multiple female sexual partners: differences in HIV prevalence and sexual risk behaviours measured at two time points." AIDS and behavior 14(6): 1330-1339.
204. Townsend, L., S. R. Rosenthal, C. D. H. Parry, Y. Zembe, C. Mathews and A. J. Flisher (2010). "Associations between alcohol misuse and risks for HIV infection among men who have multiple female sexual partners in Cape Town, South Africa." AIDS Care - Psychological and Socio-Medical Aspects of AIDS/HIV 22(12): 1544-1554.
205. Uuskula, A., L. G. Johnston, M. Raag, A. Trummal, A. Talu and D. C. Des Jarlais (2010). "Evaluating recruitment among female sex workers and injecting drug users at risk for HIV using respondent-driven sampling in Estonia." Journal of urban health : bulletin of the New York Academy of Medicine 87(2): 304-317.
206. Wang, Y., H. B. Zhang, J. Xu, G. G. Zhang, H. W. Yang and J. Fan (2010). "[Relations between self-discrimination of MSM and sexual behavior and psychological factors]. [Chinese]." Zhonghua yu fang yi xue za zhi [Chinese journal of preventive medicine] 44(7): 636-644.
207. Wejnert, C. (2010). "Social network analysis with respondent-driven sampling data: A study of racial integration on campus." Social networks 32(2): 112-124.
208. Xu, J., D. L. Han, Z. Liu, X. Y. Ma, L. L. Wang, J. Xu, L. Pang, H. B. Zhang and Z. Y. Wu (2010). "[The prevalence of HIV infection and the risk factors among MSM in 4 cities, China]. [Chinese]." Chung Hua Yu Fang i Hsueh Tsa Chih [Chinese Journal of Preventive Medicine] 44(11): 975-980.
209. Youm, Y. (2010). "A sociological interpretation of emerging properties in STI transmission dynamics: walk-betweenness of sexual networks." Sexually transmitted infections 86 Suppl 3: iii24-28.
210. Zaritsky, E. and S. L. Dibble (2010). "Risk factors for reproductive and breast cancers among older lesbians." Journal of Women's Health 19(1): 125-131.
211. Zule, W. A., C. M. Coomes, W. M. Wechsberg and H. E. Jones (2010). "Bisexual men, binge drinking and HIV risk behaviors." Alcoholism: Clinical and Experimental Research 34 (6): 282A.
212. Adeyemi, A., K. Oyediran, O. Fakunle, K. Issa and A. Azeez (2011). "Evaluating predictors of HIV infection among MSM in Nigeria towards participation in HIV prevention vaccine trial." AIDS Research and Human Retroviruses 27 (10): A81.
213. Ahmad, Z. (2011). "Sexual practices of transgender community in Pakistan." Sexually Transmitted Infections 87: A68-A69.
214. Al-Tayyib, A. A. and C. A. Rietmeijer (2011). "Detecting chlamydial and gonococcal infections through social and sexual networks." Sexually Transmitted Infections 87: A17-A18.
215. Alvarez, B., W. Miller, F. M. Hernandez, A. Alvarado, S. Morales and G. Paz-Bailey (2011). "Demographic characteristics and HIV risk behaviours among men who have sex with men in Guatemala city, Guatemala, 2010." Sexually Transmitted Infections 87: A150.
216. Armstrong, G., C. Humtsoe and M. Kermode (2011). "HIV risk behaviours among injecting drug users in Northeast India following scale-up of a targeted HIV prevention programme." BMC public health 11 Suppl 6: S9.
217. Balan, I. C., A. Carballo-Dieguez, R. Marone, C. Dolezal, M. A. Pando, V. Barreda and M. M. Avila (2011). "Alcohol, drug use, and sexual risk behavior among a diverse sample of men who have sex with men in Buenos Aires, Argentina." Alcoholism: Clinical and Experimental Research 35: 278A.
218. Benzaken, A. S., E. G. Galban, N. S. Benzaken, C. K, A. Pinho, M. Mello, F. Vasquez, C. A. Barros and L. R. Franco Sansigolo Kerr (2011). "Behavioural and biological surveillance among man who have sex with man using respondentdriven sampling methodology in Manaus, Amazon, Brazil." Sexually Transmitted Infections 87: A141.
219. Berg, C. J., E. J. Nehl, F. Y. Wong, N. He, Z. Jennifer Huang, J. S. Ahluwalia and T. Zheng (2011). "Prevalence and correlates of tobacco use among a sample of MSM in Shanghai, China." Nicotine and Tobacco Research 13(1): 22-28.
220. Bermudez-Aza, E. H., L. R. F. S. Kerr, C. Kendall, A. A. Pinho, M. B. De Mello, R. S. Mota, M. D. C. Guimaraes, C. S. Alencar, A. M. De Brito, I. C. Dourado, S. M. B. Da Batista, F. Abreu, L. C. De Oliveira, A. De Souza Moraes, A. S. Benzaken, E. Merchan-Hamann, G. M. B. De Freitas, W. McFarland, E. Albuquerque, G. W. Rutherford and E. Sabino (2011). "Antiretroviral drug resistance in a respondent-driven sample of HIV-infected men who have sex with men in Brazil." Journal of Acquired Immune Deficiency Syndromes 57(SUPPL. 3): S186-S192.
221. Boodram, B., R. C. Hershow, D. Klinzman and J. T. Stapleton (2011). "GB virus C infection among young, HIV-negative injection drug users with and without hepatitis C virus infection." Journal of Viral Hepatitis 18(4): e153-e159.
222. Busari, O., M. Nakayima and A. Busari (2011). "Female clients and partners of msm sex workers in a resource-poor setting, West Africa." Sexually Transmitted Infections 87: A126-A127.
223. Busaro, O. A., M. Nakayima, A. Adeyemi and S. Agboola (2011). "HIV and STI prevalence among men who have sex with men recruited through respondent driven sample in a nigerian oil-rich port Harcourt City, Nigeria." AIDS Research and Human Retroviruses 27 (10): A82.
224. Calafat, A., N. T. Blay, K. Hughes, M. Bellis, M. Juan, M. Duch and A. Kokkevi (2011). "Nightlife young risk behaviours in Mediterranean versus other European cities: are stereotypes true?" European journal of public health 21(3): 311-315.
225. Carballo-Dieguez, A., I. Balan, R. Marone, M. A. Pando, C. Dolezal, V. Barreda, C. S. Leu and M. M. Avila (2011). "Use of respondent driven sampling (RDS) generates a very diverse sample of men who have sex with men (MSM) in Buenos Aires, Argentina." PLoS ONE 30.
226. Curran, G. M., B. M. Booth and T. Borders (2011). "Perceived need for substance use treatment among active african american substance users: A qualitative study in rural and urban locations." Alcoholism: Clinical and Experimental Research 35: 202A.
227. Dahoma, M., L. G. Johnston, A. Holman, L. A. Miller, M. Mussa, A. Othman, A. Khatib, R. Issa, C. Kendall and A. A. Kim (2011). "HIV and related risk behavior among men who have sex with men in Zanzibar, Tanzania: results of a behavioral surveillance survey." AIDS and behavior 15(1): 186-192.
228. Damacena, G. N., C. L. Szwarcwald and A. Barbosa Jr (2011). "Implementation of respondent-driven sampling among female sex workers in Brazil, 2009. [Portuguese] Implementacao do metodo de amostragem respondent-driven sampling entre mulheres profissionais do sexo no Brasil, 2009." Cadernos de Saude Publica 27(SUPPL. 1): S45-S55.
229. Damacena, G. N., C. L. Szwarcwald, P. R. B. De Souza Junior and I. Dourado (2011). "Risk factors associated with HIV prevalence among female sex workers in 10 Brazilian Cities." Journal of Acquired Immune Deficiency Syndromes 57(SUPPL. 3): S144-S152.
230. Des Jarlais, D. C., S. Vorobjov, K. Arasteh, A. Uuskula and D. C. Perlman (2011). "Can non-injecting drug use protect against blood-borne viruses among people who inject drugs? Evidence from Tallinn, Estonia, and New York City, USA." Journal of the International Association of Physicians in AIDS Care 10 (3): 203.
231. Erausquin, J. T., E. Reed and K. M. Blankenship (2011). "Police-related experiences and HIV risk among female sex workers in Andhra Pradesh, India." Journal of Infectious Diseases 204(SUPPL. 5): S1223-S1228.
232. Evans, A. R., G. J. Hart, R. Mole, C. H. Mercer, V. Parutis, C. J. Gerry, J. Imrie and F. M. Burns (2011). "Central and East European migrant men who have sex with men in London: a comparison of recruitment methods." BMC Medical Research Methodology 11(69).
233. Gelpi-Acosta, C., H. Hagan, S. M. Jenness, T. Wendel and A. Neaigus (2011). "Sexual and injection-related risks in Puerto Rican-born injection drug users living in New York City: A mixed-methods analysis." Harm Reduction Journal 8(28).
234. Gupta, J., E. Reed, T. Kershaw and K. M. Blankenship (2011). "History of sex trafficking, recent experiences of violence, and HIV vulnerability among female sex workers in coastal Andhra Pradesh, India." International Journal of Gynecology and Obstetrics 114(2): 101-105.
235. Gwadz, M. V., N. R. Leonard, C. M. Cleland, M. Riedel, A. Banfield, D. Mildvan and A. C. T. P. C. R. Team (2011). "The effect of peer-driven intervention on rates of screening for AIDS clinical trials among African Americans and Hispanics." American journal of public health 101(6): 1096-1102.
236. Hao, C., H. Yan, H. Yang, X. Huan, W. Guan, X. Xu, M. Zhang, W. Tang, N. Wang, J. Gu and J. T. F. Lau (2011). "The incidence of syphilis, HIV and HCV and associated factors in a cohort of men who have sex with men in Nanjing, China." Sexually Transmitted Infections 87(3): 199-201.
237. Hernandez, F., N. Arambu, B. Alvarez, L. Romero, E. J. Goins, G. Paz-Bailey and S. Morales (2011). "High incidence of HIV and low HIV prevention coverage among men who have sex with men in Managua, Nicaragua." Sexually Transmitted Infections 87: A146.
238. Hope, V., A. Jeannin, B. Spencer, J. P. Gervasoni, M. J. v. d. Laar and F. Dubois-Arber (2011). "Mapping HIV-related behavioural surveillance among injecting drug users in Europe, 2008." Eurosurveillance 16(36): 19960.
239. Hope, V. D., M. Hickman, S. L. Ngui, S. Jones, M. Telfer, M. Bizzarri, F. Ncube and J. V. Parry (2011). "Measuring the incidence, prevalence and genetic relatedness of hepatitis C infections among a community recruited sample of injecting drug users, using dried blood spots." Journal of Viral Hepatitis 18(4): 262-270.
240. Hopfer, S., J. Wylie and X. Tan (2011). "Risk profiles of winnipeg street populations: A latent class analysis." Sexually Transmitted Infections 87: A246-A247.
241. Jacobson, J. O., M. Alonso-Gonzalez, D. Ramachandran, S. Morales-Miranda, A. Carballo-Dieguez, J. Medrano, T. Solano and M. D. Rosales-Perez (2011). "Crack/cocaine use among MSM in Latin America: A multilevel analysis of RDS studies to identify drug use patterns and associated factors across cities." Sexually Transmitted Infections 87: A150.
242. Jacobson, J. O., M. Alonso-Gonzalez, D. Ramachandran, G. Paz-Bailey, I. Balan, S. Morales-Miranda, M. E. Guardado, R. Salamanca-Kacic, A. I. Nieto-Gomez and M. Maddaleno (2011). "Regional hiv surveillance of youth MSM through multilevel analysis of rds studies in latin America." Sexually Transmitted Infections 87: A29.
243. Jeffri, J., D. D. Heckathorn and M. W. Spiller (2011). "Painting your life: A study of aging visual artists in New York City." Poetics 39(1): 19-43.
244. Jenness, S. M., E. M. Begier, A. Neaigus, C. S. Murrill, T. Wendel and H. Hagan (2011). "Unprotected anal intercourse and sexually transmitted diseases in high-risk heterosexual women." American journal of public health 101(4): 745-750.
245. Jenness, S. M., H. Hagan, K. L. Liu, T. Wendel and C. S. Murrill (2011). "Continuing HIV risk in New York City injection drug users: the association of syringe source and syringe sharing." Substance use & misuse 46(2-3): 192-200.
246. Jenness, S. M., P. Kobrak, T. Wendel, A. Neaigus, C. S. Murrill and H. Hagan (2011). "Patterns of exchange sex and HIV infection in high-risk heterosexual men and women." Journal of urban health : bulletin of the New York Academy of Medicine 88(2): 329-341.
247. Jenness, S. M., A. Neaigus, C. S. Murrill, T. Wendel, L. Forgione and H. Hagan (2011). "Estimated HIV incidence among high-risk heterosexuals in New York city, 2007." Journal of Acquired Immune Deficiency Syndromes 56(2): 193-197.
248. Johnston, L., A. Saumtally, S. Corceal, I. Mahadoo and F. Oodally (2011). "High HIV and hepatitis C prevalence amongst injecting drug users in Mauritius: Findings from a population size estimation and respondent driven sampling survey." International Journal of Drug Policy 22(4): 252-258.
249. Kassak, K., Z. Mahfoud, K. Kreidieh, S. Shamra, R. Afifi and S. Ramia (2011). "Hepatitis B virus and hepatitis C virus infections among female sex workers and men who have sex with men in Lebanon: Prevalence, risk behaviour and immune status." Sexual Health 8(2): 229-233.
250. Khamsiriwatchara, A., P. Wangroongsarb, J. Thwing, J. Eliades, W. Satimai, C. Delacollette and J. Kaewkungwal (2011). "Respondent-driven sampling on the Thailand-Cambodia border. I. Can malaria cases be contained in mobile migrant workers?" Malaria Journal 10(120).
251. Khan, M. S., M. Unemo, S. Zaman and C. S. Lundborg (2011). "HIV, STI prevalence and risk behaviours among women selling sex in Lahore, Pakistan." BMC Infectious Diseases 11(119).
252. Khan, M. S., M. Unemo, S. Zaman and C. Stalsby Lundborg (2011). "Health-seeking behaviour of women selling sex in Lahore, Pakistan." International Journal of STD and AIDS 22(7): 376-380.
253. Kogan, S. M., G. H. Brody, Y. F. Chen and R. J. DiClemente (2011). "Self-regulatory problems mediate the association of contextual stressors and unprotected intercourse among rural, African American, young adult men." Journal of health psychology 16(1): 50-57.
254. Kogan, S. M., C. Wejnert, Y.-f. Chen, G. H. Brody and L. M. Slater (2011). "Respondent-driven sampling with hard-to-reach emerging adults: An introduction and case study with rural African Americans." Journal of Adolescent Research 26(1): 30-60.
255. Koram, N., H. Liu, J. Li, J. Luo and J. Nield (2011). "Role of social network dimensions in the transition to injection drug use: actions speak louder than words." AIDS and behavior 15(7): 1579-1588.
256. Korhonen, L. D., M. Sobota, D. Tranter and K. Hudson (2011). "Engaging populations at risk - Strengthening connections." Canadian Journal of Infectious Diseases and Medical Microbiology 22: 117B.
257. Kurtz, S. P., H. Surratt, T. Cicero, G. Ibanez, A. Rosenblum and R. Dart (2011). "Internet-based diversion of prescription opioids." Pain Medicine 12 (3): 476.
258. Lane, T., H. F. Raymond, S. Dladla, J. Rasethe, H. Struthers, W. McFarland and J. McIntyre (2011). "High HIV prevalence among men who have sex with men in Soweto, South Africa: results from the Soweto Men's Study." AIDS and behavior 15(3): 626-634.
259. Lansky, A., E. A. DiNenno and C. Wejnert (2011). "Use of respondent-driven sampling for monitoring HIV behaviours among injecting drug users in the united states." Sexually Transmitted Infections 87: A16.
260. Lee, R., J. Ranaldi, M. Cummings, J. B. Crucetti, H. Stratton and L. A. McNutt (2011). "Given the Increasing Bias in Random Digit Dial Sampling, Could Respondent-Driven Sampling be a Practical Alternative?" Annals of Epidemiology 21(4): 272-279.
261. Leonard, L., E. A. Medd, S. McWilliam, E. DeRubeis, A. Germain and A. Reynolds (2011). "Women in Ottawa who smoke crack engage in riskier smoking practices compared to Ottawa men who smoke crack." Canadian Journal of Infectious Diseases and Medical Microbiology 22: 80B-81B.
262. Leonard, L., E. A. Medd, S. McWilliam, E. DeRubeis, A. Germain and A. Reynolds (2011). "A need for gender-specific harm reduction services in ottawa: Women IDUs report more binging on injection drugs and using previously used injection equipment compared to men." Canadian Journal of Infectious Diseases and Medical Microbiology 22: 80B.
263. Li, J., H. Liu, J. Li, J. Luo, D. d. Jarlais and N. Koram (2011). "Role of sexual transmission of HIV among young noninjection and injection opiate users: a respondent-driven sampling study." Sexually Transmitted Diseases 38(12): 1161-1166.
264. Li, J., H. Liu, H. Liu, T. Feng and Y. Cai (2011). "Psychometric assessment of HIV/STI sexual risk scale among MSM: a Rasch model approach." BMC Public Health 11(763).
265. Li, J., H. J. Liu, J. Li, J. Luo, N. Koram and R. Detels (2011). "Sexual transmissibility of HIV among opiate users with concurrent sexual partnerships: an egocentric network study in Yunnan, China." Addiction 106(10): 1780-1787.
266. Lin, G., Y. Chen, C. Chen and B. Jiang (2011). "Investigation on knowledge, attitude and practice among MSM in Benxi City. [Chinese]." Occupation and Health 27(5): 532-534.
267. Liu, H. and J. Li (2011). "Verification of random selection assumption in respondent-driven sampling in egocentric social network data." American Journal of Epidemiology 173: S110.
268. Liu, H., J. Li, J. Luo, D. Des Jarlais and N. Koram (2011). "Role of sexual transmission of HIV among young noninjection and injection opiate users: A respondent-driven sampling study." Sexually Transmitted Diseases 38(12): 1161-1166.
269. Liu, H., J. Li, J. Luo, N. Koram and R. Detels (2011). "Sexual transmissibility of HIV among opiate users with concurrent sexual partnerships: an egocentric network study in Yunnan, China." Addiction (Abingdon, England) 106(10): 1780-1787; discussion 1788-1789.
270. Liu, J., B. Qu, H. Q. Guo and G. Sun (2011). "Factors that influence risky sexual behaviors among men who have sex with men in Liaoning Province, China: A structural equation model." AIDS Patient Care and STDs 25(7): 423-429.
271. Liu, T., M. Liao, H. Zhang, X. Nie, R. Pan, X. Tao, D. Kang and B. Jiang (2011). "Health seeking behavior and influencing factors among female sex workers in Jinan. [Chinese]." Journal of Shandong University 49(1): 115-119.
272. Makyao, N., A. Kangolle, A. Gilly, G. Somi, M. Kazaura, E. Kim, M. Kibona and S. Kamazima (2011). "High hiv prevalence within a generalised epidemic; condom use, violence, and sexually transmitted infections among female sex workers in DAR ES Salaam, Tanzania." Sexually Transmitted Infections 87: A40-A41.
273. Malekinejad, M., W. McFarland, J. Vaudrey and H. F. Raymond (2011). "Accessing a diverse sample of injection drug users in San Francisco through respondent-driven sampling." Drug and Alcohol Dependence 118(2-3): 83-91.
274. McCreesh, N., S. Frost, J. Seeley, J. Katongole, M. Ndagire Tarsh, R. Ndungutse, F. Jichi, D. Maher, P. Sonnenberg, A. Copas, R. J. Hayes and R. G. White (2011). "An empirical evaluation of respondent-driven sampling." Sexually Transmitted Infections 87: A15-A16.
275. McCreesh, N., L. G. Johnston, A. Copas, P. Sonnenberg, J. Seeley, R. J. Hayes, S. D. Frost and R. G. White (2011). "Evaluation of the role of location and distance in recruitment in respondent-driven sampling." International Journal of Health Geographics [Electronic Resource] 10(56).
276. Merrigan, M., A. Azeez, B. Afolabi, O. N. Chabikuli, O. Onyekwena, G. Eluwa, B. Aiyenigba, I. Kawu, K. Ogungbemi and C. Hamelmann (2011). "HIV prevalence and risk behaviours among men having sex with men in Nigeria." Sexually Transmitted Infections 87(1): 65-70.
277. Miller, W., B. Alvarez, S. Boyce, A. Alvarado, C. Barrington and G. Paz-Bailey (2011). "Transgender persons in guatemala - Overexposed and under-protected - The findings of an RDS behavioural survey." Sexually Transmitted Infections 87: A132.
278. Mmbaga, E. J., M. J. Dodo, G. H. Leyna, K. Moen and M. T. Leshabari (2011). "Sexual practices and perceived susceptibility to HIV infection among men who have sex with men in Dar Es Salaam, mainland Tanzania. (Special Issue: Risk behaviors: HIV/AIDS - Volume 1.)." Journal of AIDS and Clinical Research 1(012).
279. Montealegre, J., J. Risser and B. J. Selwyn (2011). "HIV Testing behaviours among undocumented central american immigrant women in Houston, Texas, 2010." Sexually Transmitted Infections 87: A264-A265.
280. Montealegre, J., J. Risser, B. J. Selwyn and K. Sabin (2011). "Effectiveness of respondent driven sampling among undocumented central American immigrant women in houston, texas, 2010." Sexually Transmitted Infections 87: A174.
281. Montealegre, J. R., B. J. Selwyn, K. Sabin, S. A. McCurdy and J. M. Risser (2011). "Healthcare access and use among undocumented Central American immigrant women in Houston, Texas, and its implications for cervical cancer screening." Cancer Epidemiology Biomarkers and Prevention 20 (10 Meeting Abstracts).
282. Montealegre, J. R., B. J. Selwyn, K. Sabin and J. Risser (2011). "Using respondent-driven sampling to study healthcare and screening behaviors among undocumented immigrant women in the U.S." Cancer Epidemiology Biomarkers and Prevention 20 (10 Meeting Abstracts).
283. Mota, R. M. S., L. R. F. S. Kerr, C. Kendall, A. Pinho, M. B. d. Mello, I. Dourado, M. D. C. Guimaraes, A. Brito, S. Batista, F. Abreu, A. Benzaken, L. Oliveira, A. Moraes, E. Merchan-Hamann, G. Freitas, E. M. Albuquerque, W. McFarland and G. Rutherford (2011). "Reliability of self-report of HIV status among men who have sex with men in Brazil. (AIDS research in Brazil.)." JAIDS, Journal of Acquired Immune Deficiency Syndromes 57(Suppl. 3): S153-S156.
284. Pando, M., R. Marone, I. Balan, C. Dolezal, V. Barreda, A. Carballo-Dieguez and M. M. Avila (2011). "Circumcision and HIV acquisition risk among men who have sex with men (MSM) in Buenos Aires, Argentina." AIDS Research and Human Retroviruses 27 (10): A78.
285. Paquette, D. M., J. Bryant, S. Crawford and J. B. F. de Wit (2011). "Conducting a respondent-driven sampling survey with the use of existing resources in Sydney, Australia." Drug and Alcohol Dependence 116(1-3): 125-131.
286. Paquette, D. M., J. Bryant and J. De Wit (2011). "Use of respondent-driven sampling to enhance understanding of injecting networks: A study of people who inject drugs in Sydney, Australia." International Journal of Drug Policy 22(4): 267-273.
287. Paz-Bailey, G., B. Alvarez, W. Miller, B. Sabrina, C. Barrington, A. Kim, S. Morales and S. Chen (2011). "Population size estimates for men who have sex with men in Guatemala city using time location sampling and respondent driven sampling." Sexually Transmitted Infections 87: A163.
288. Paz-Bailey, G., J. O. Jacobson, M. E. Guardado, F. M. Hernandez, A. I. Nieto, M. Estrada and J. Creswell (2011). "How many men who have sex with men and female sex workers live in El Salvador? Using respondent-driven sampling and capture-recapture to estimate population sizes." Sexually Transmitted Infections 87(4): 279-282.
289. Pervaiz, A. (2011). "Respondent driven sampling with hard-to-reach migrant workers in Pakistan: Experiences and lessons learned." Canadian Journal of Infectious Diseases and Medical Microbiology 22: 107B.
290. Qiu, P., E. Caine, Y. Yang, Q. Chen, J. Li and X. Ma (2011). "Depression and associated factors in internal migrant workers in China." Journal of Affective Disorders 134(1-3): 198-207.
291. Reed, E., J. Gupta, M. Biradavolu, V. Devireddy and K. M. Blankenship (2011). "The role of housing in determining HIV risk among female sex workers in Andhra Pradesh, India: Considering women's life contexts." Social Science and Medicine 72(5): 710-716.
292. Rispel, L. C., C. A. Metcalf, A. Cloete, J. Moorman and V. Reddy (2011). "You become afraid to tell them that you are gay: health service utilization by men who have sex with men in South African cities. (Special Issue: Public health, health sector reforms, and policy implementation in South Africa: studies and perspectives on the 24th anniversary of the centre for health policy.)." Journal of Public Health Policy 32(S1): S137-S151.
293. Rispel, L. C., C. A. Metcalf, A. Cloete, V. Reddy and C. Lombard (2011). "HIV prevalence and risk practices among men who have sex with men in two South African Cities." Journal of Acquired Immune Deficiency Syndromes 57(1): 69-76.
294. Risser, J., P. Padgett and J. Montealegre (2011). "Can rds be used to recruit unbiased samples from the same population with repeated sampling?" Sexually Transmitted Infections 87: A174-A175.
295. Risser, J., P. Padgett and J. Montealegre (2011). "Failure of respondent driven sampling in a transgender population due to inadequate formative work and non-network associated selection criteria." Sexually Transmitted Infections 87: A173.
296. Rudolph, A. E., N. D. Crawford, C. Latkin, R. Heimer, E. O. Benjamin, K. C. Jones and C. M. Fuller (2011). "Subpopulations of Illicit Drug Users Reached by Targeted Street Outreach and Respondent-Driven Sampling Strategies: Implications for Research and Public Health Practice." Annals of Epidemiology 21(4): 280-289.
297. Rudolph, A. E., N. D. Crawford, C. Latkin, K. White, E. O. Benjamin, K. Jones and C. M. Fuller (2011). "Individual, study, and neighborhood level characteristics associated with peer recruitment of young illicit drug users in New York City: Optimizing respondent driven sampling." Social Science and Medicine 73(7): 1097-1104.
298. Rudolph, A. E., K. C. Jones, C. Latkin, N. D. Crawford and C. M. Fuller (2011). "The association between parental risk behaviors during childhood and having high risk networks in adulthood." Drug and Alcohol Dependence 118(2-3): 437-443.
299. Rudolph, A. E., C. Latkin, N. D. Crawford, K. C. Jones and C. M. Fuller (2011). "Does respondent driven sampling alter the social network composition and health-seeking behaviors of illicit drug users followed prospectively?" PloS one 6(5): e19615.
300. Salani Mota, R. M., L. R. F. S. Kerr, C. Kendall, A. Pinho, M. B. De Mello, I. Dourado, M. D. C. Guimaraes, A. Brito, S. Batista, F. Abreu, A. Benzaken, L. Oliveira, A. Moraes, E. Merchan-Hamann, G. Freitas, E. M. Albuquerque, W. McFarland and G. Rutherford (2011). "Reliability of self-report of HIV status among men who have sex with men in Brazil." Journal of Acquired Immune Deficiency Syndromes 57(SUPPL. 3): S153-S156.
301. Sarin, E., L. Samson, M. Sweat and C. Beyrer (2011). "Human rights abuses and suicidal ideation among male injecting drug users in Delhi, India." International Journal of Drug Policy 22(2): 161-166.
302. Sayarifard, A., A. Kolahi and M. H. Hamedani (2011). "Frequency of performing HIV test and reasons of not-testing among female sex workers." American Journal of Epidemiology 173: S197.
303. Sayarifard, A., A. A. Kolahi and M. A. Hajjar Hamedani (2011). "Frequency of unprotected sexual behaviors regarding HIV/AIDS transmission in at-risk women." American Journal of Epidemiology 173: S198.
304. Sayarifard, A., A. A. Kolahi and M. H. Hamedani (2011). "The vision of female sex workers and their noncommercial sexual partners regarding their perilous role." American Journal of Epidemiology 173: S150.
305. Shah, N. S., R. W. Shiraishi, W. Subhachaturas, A. Anand, S. J. Whitehead, S. Tanpradech, C. Manopaiboon, K. M. Sabin, K. K. Fox and A. Y. Kim (2011). "Bridging populations-sexual risk behaviors and HIV prevalence in clients and partners of female sex workers, Bangkok, Thailand 2007." Journal of urban health : bulletin of the New York Academy of Medicine 88(3): 533-544.
306. Shaw, S., K. Deering, A. Jolly and J. Wylie (2011). "Outlier populations: Heightened risk for HIV, HCV and HIV/HCV co-infection among solvent-using injection drug users." Sexually Transmitted Infections 87: A65.
307. Shaw, S. Y., K. N. Deering, A. M. Jolly and J. L. Wylie (2011). "Outlier populations: Elevated risk for HIV, HCV and HIV/HCV co-infection among solvent-using injection drug users in manitoba." Canadian Journal of Infectious Diseases and Medical Microbiology 22: 22B.
308. Sobota, M., D. Tranter, K. Hudson and L. D. Korhonen (2011). "Engaging populations at risk - Unique approach to data collection from at risk populations." Canadian Journal of Infectious Diseases and Medical Microbiology 22: 105B-106B.
309. Sonali, W., F. Cowan, P. Warner, A. Copas, D. Mabey and S. Maryam (2011). "Contraceptive practices, sexual and reproductive health needs of HIV-positive and negative female sex workers in Goa, India." Sexually Transmitted Infections 87(1): 58-64.
310. Taran, Y. S., L. G. Johnston, N. B. Pohorila and T. O. Saliuk (2011). "Correlates of HIV risk among injecting drug users in sixteen Ukrainian cities." AIDS and behavior 15(1): 65-74.
311. Thayansin, S. (2011). "Respondent-Driven Sampling: reaching male youth with drug use experience in Thailand." Journal of Population and Social Studies 20(1): 43-56.
312. Townsend, L., R. Jewkes, C. Mathews, L. G. Johnston, A. J. Flisher, Y. Zembe and M. Chopra (2011). "HIV risk behaviours and their relationship to intimate partner violence (IPV) among men who have multiple female sexual partners in Cape Town, South Africa." AIDS and behavior 15(1): 132-141.
313. Townsend, L., C. Mathews and Y. Zembe (2011). "Intimate partner sexual violence and its relationship to HIV sexual risk behaviour among men who have multiple female sexual partners in Cape Town, South Africa." Journal of Sexual Medicine 8: 140.
314. Uuskula, A., D. C. Des Jarlais, M. Kals, K. Ruutel, K. Abel-Ollo, A. Talu and I. Sobolev (2011). "Expanded syringe exchange programs and reduced HIV infection among new injection drug users in Tallinn, Estonia." BMC Public Health 11(517).
315. Volkmann, T., R. Lozada, C. M. Anderson, T. L. Patterson, A. Vera and S. A. Strathdee (2011). "Factors associated with drug-related harms related to policing in Tijuana, Mexico." Harm Reduction Journal 8(7).
316. Wagner, K. D., R. A. Pollini, T. L. Patterson, R. Lozada, V. D. Ojeda, K. C. Brouwer, A. Vera, T. A. Volkmann and S. A. Strathdee (2011). "Cross-border drug injection relationships among injection drug users in Tijuana, Mexico." Drug and Alcohol Dependence 113(2-3): 236-241.
317. Wall, M., E. Schmidt, A. Sarang, R. Atun and A. Renton (2011). "Sex, drugs and economic behaviour in Russia: A study of socio-economic characteristics of high risk populations." International Journal of Drug Policy 22(2): 133-139.
318. Wang, N., M. Zhang and S. Wu (2011). "Survey of knowledge, attitude and behavior related to AIDS among man who have sex with man (MSM) in Nanjing. [Chinese]." Modern Preventive Medicine 38(1): 117-118.
319. Wang, W., L. Xue and Y. Chen (2011). "Study on vulnerability to STD/HTV infections and prevention/control among MSM in Kunshan City, Jiangsu Province. [Chinese]." China Tropical Medicine 11(3): 296-298.
320. Wangroongsarb, P., W. Satimai, A. Khamsiriwatchara, J. Thwing, J. M. Eliades, J. Kaewkungwal and C. Delacollette (2011). "Respondent-driven sampling on the Thailand-Cambodia border. II. Knowledge, perception, practice and treatment-seeking behaviour of migrants in malaria endemic zones." Malaria Journal 10(117).
321. Wayal, S., F. Cowan, P. Warner, A. Copas, D. Mabey and M. Shahmanesh (2011). "Contraceptive practices, sexual and reproductive health needs of HIV-positive and negative female sex workers in Goa, India." Sexually Transmitted Infections 87(1): 58-64.
322. Wei, C., S. Ruan, J. Zhao, H. Yang, Y. Zhu and H. F. Raymond (2011). "Which Chinese men who have sex with men miss out on HIV testing?" Sexually Transmitted Infections 87(3): 225-228.
323. Zhang, H., M. Liao, X. Nie, R. Pan, C. Wang, S. Ruan, C. Zhang, X. Tao, D. Kang and B. Jiang (2011). "Predictors of consistent condom use based on the Information-Motivation-Behavioral Skills (IMB) model among female sex workers in Jinan, China." BMC Public Health 11(113).
324. Zhong, F., P. Lin, H. Xu, Y. Wang, M. Wang, Q. He, L. Fan, Y. Li, F. Wen, Y. Liang, H. F. Raymond and J. Zhao (2011). "Possible increase in HIV and syphilis prevalence among men who have sex with men in Guangzhou, China: results from a respondent-driven sampling survey." AIDS and behavior 15(5): 1058-1066.
325. Adebajo, S. B., G. I. Eluwa, D. Allman, T. Myers and B. A. Ahonsi (2012). "Prevalence of internalized homophobia and HIV associated risks among men who have sex with men in Nigeria." African journal of reproductive health 16(4): 21-28.
326. Ahmadi, K., M. Rezazade, M. Nafarie, B. Moazen, M. Y. Vasel and S. Assari (2012). "Unprotected sex with injecting drug users among Iranian female sex workers: unhide HIV risk study." AIDS Research and Treatment 651070(36).
327. Avina, S., T. Waimar, B. Aruna, D. Lewis, Y. S. Singh and L. Apicella (2012). "Assessment of unsafe injection practices and sexual behaviors among male injecting drug users in two urban cities of India using respondent driven sampling." Southeast Asian Journal of Tropical Medicine and Public Health 43(3): 652-667.
328. Barrington, C., C. Wejnert, M. E. Guardado, A. I. Nieto and G. P. Bailey (2012). "Social network characteristics and HIV vulnerability among transgender persons in San Salvador: identifying opportunities for HIV prevention strategies." AIDS and behavior 16(1): 214-224.
329. Barua, P., J. Mahanta, G. K. Medhi, J. Dale, R. S. Paranjape and G. Thongamba (2012). "Sexual activity as risk factor for hepatitis C virus (HCV) transmission among the female sex workers in Nagaland." Indian Journal of Medical Research, Supplement 136(SUPPL): 30-35.
330. Bauer, G. R., R. Travers, K. Scanlon and T. A. Coleman (2012). "High heterogeneity of HIV-related sexual risk among transgender people in Ontario, Canada: a province-wide respondent-driven sampling survey." BMC Public Health 12(292).
331. Bauermeister, J. A., M. A. Zimmerman, M. M. Johns, P. Glowacki, S. Stoddard and E. Volz (2012). "Innovative recruitment using online networks: Lessons learned from an online study of alcohol and other drug use utilizing a web-based, Respondent- Driven Sampling (webRDS) strategy." Journal of Studies on Alcohol and Drugs 73(5): 834-838.
332. Bengtsson, L., X. Lu, Q. C. Nguyen, M. Camitz, N. L. Hoang, T. A. Nguyen, F. Liljeros and A. Thorson (2012). "Implementation of web-based respondent-driven sampling among men who have sex with men in Vietnam." PLoS ONE [Electronic Resource] 7(11).
333. Bernhardt, A., M. Spiller and N. Theodore (2012). "Employers gone rogue: Explaining industry variation in violations of workplace laws." Ind Lab Rel Rev Available at SSRN 2013376 66(4): 808-832.
334. Berry, M., A. L. Wirtz, A. Janayeva, V. Ragoza, A. Terlikbayeva, B. Amirov, S. Baral and C. Beyrer (2012). "Risk factors for HIV and unprotected anal intercourse among men who have sex with men (MSM) in Almaty, Kazakhstan." PLoS ONE 7(8).
335. Borders, T. F. and B. M. Booth (2012). "Stimulant use trajectories and the longitudinal risk of heavy drinking: Findings from a rural population-based study." Addictive Behaviors 37(3): 269-272.
336. Bozicevic, I., S. Z. Lepej, O. D. Rode, I. Grgic, P. Jankovic, Z. Dominkovic, D. Lukas, L. G. Johnston and J. Begovac (2012). "Prevalence of HIV and sexually transmitted infections and patterns of recent HIV testing among men who have sex with men in Zagreb, Croatia." Sexually Transmitted Infections 88(7): 539-544.
337. Brouwer, K., M. L. Rusch, J. R. Weeks, R. Lozada, A. Vera, C. Magis-Rodriguez and S. Strathdee (2012). "Spatial epidemiology of HIV among injection drug users in Tijuana, Mexico." Annals of the Association of American Geographers 102(5): 1190-1199.
338. Burkett, J. G., P. Parson, K. Lapane and P. Boling (2012). "Engaging an invisible population: African american elders in public housing." Journal of the American Geriatrics Society 60: S219.
339. Burnham, G., S. Malik, A. S. Dhari Al-Shibli, A. R. Mahjoub, A. Q. Baqer, Z. Q. Baqer, F. A. Qaraghuli and S. Doocy (2012). "Understanding the impact of conflict on health services in Iraq: Information from 401 Iraqi refugee doctors in Jordan." International Journal of Health Planning and Management 27(1): e51-e64.
340. Burt, R. D. and H. Thiede (2012). "Evaluating Consistency in Repeat Surveys of Injection Drug Users Recruited by Respondent-Driven Sampling in the Seattle Area: Results from the NHBS-IDU1 and NHBS-IDU2 Surveys." Annals of Epidemiology 22(5): 354-363.
341. Carballo-Dieguez, A., I. Balan, C. Dolezal and M. B. Mello (2012). "Recalled sexual experiences in childhood with older partners: a study of Brazilian men who have sex with men and male-to-female transgender persons." Archives of sexual behavior 41(2): 363-376.
342. Chen, R., Z. Li and Y. Qiu (2012). "Study on effect of AIDS high-risk behaviour interventions by Tengxun QQ among MSM in Puning city. [Chinese]." Journal of Tropical Medicine 12(8): 1022-1024.
343. Cranston, R. D., R. Murphy, R. E. Weiss, M. Da Costa, J. Palefsky, S. Shoptaw and P. M. Gorbach (2012). "Anal human papillomavirus infection in a street-based sample of drug using HIV-positive men." International Journal of STD and AIDS 23(3): 195-200.
344. Creswell, J., M. E. Guardado, J. Lee, A. I. Nieto, A. A. Kim, E. Monterroso and G. Paz-Bailey (2012). "HIV and STI control in El Salvador: Results from an integrated behavioural survey among men who have sex with men." Sexually Transmitted Infections 88(8): 633-638.
345. Curran, G. M., B. M. Booth and A. M. Cheney (2012). "Barriers to help-seeking for problem drinking/drug use among the national guard." Alcoholism: Clinical and Experimental Research 36: 167A.
346. Daniulaityte, R., R. Falck and R. G. Carlson (2012). "Illicit use of buprenorphine in a community sample of young adult non-medical users of pharmaceutical opioids." Drug and Alcohol Dependence 122(3): 201-207.
347. Daniulaityte, R., R. Falck, L. Li, R. W. Nahhas and R. G. Carlson (2012). "Respondent-driven sampling to recruit young adult non-medical users of pharmaceutical opioids: Problems and solutions." Drug and Alcohol Dependence 121(1-2): 23-29.
348. De La Rosa, M., R. Babino, A. Rosario, N. V. Martinez and L. Aijaz (2012). "Challenges and strategies in recruiting, interviewing, and retaining recent latino immigrants in substance abuse and HIV epidemiologic studies." American Journal on Addictions 21(1): 11-22.
349. Deshpande, S., A. Kohli, S. Rathod, M. Mainkar, S. Kazi, D. Pardeshi, J. Dale, S. Aralkar, N. Panchal, U. Mahajan and R. Paranjape (2012). "Recruitment of at-risk population through network based sampling: Experiences from Maharashtra IBBA Round-I survey." Indian Journal of Medical Research, Supplement 136(SUPPL): 36-43.
350. Erausquin, J. T., M. Biradavolu, E. Reed, R. Burroway and K. M. Blankenship (2012). "Trends in condom use among female sex workers in Andhra Pradesh, India: the impact of a community mobilisation intervention." Journal of epidemiology and community health 66 Suppl 2: ii49-54.
351. Fan, S., H. Lu, X. Ma, Y. Sun, X. He, C. Li, H. F. Raymond, W. McFarland, J. Sun, W. Ma, Y. Jia, Y. Xiao, Y. Shao and Y. Ruan (2012). "Behavioral and serologic survey of men who have sex with Men in Beijing, China: Implication for HIV intervention." AIDS Patient Care and STDs 26(3): 148-155.
352. Gele, A. A., E. B. Johansen, M. I. Huage and J. Sundby (2012). "When female circumcision comes to the West: attitudes toward the practice among Somali immigrants in Oslo." BMC Public Health 12(697).
353. Gele, A. A., E. B. Johansen and J. Sundby (2012). "When female circumcision comes to the West: attitudes toward the practice among Somali Immigrants in Oslo." BMC public health 12: 697.
354. Guo, S., H. Zhou and X. Cao (2012). "Usage of condom and the effect factors among the floating women in Chengdu city. [Chinese]." Maternal and Child Health Care of China 27(9): 1306-1308.
355. Hakansson, A., P. Isendahl, C. Wallin and M. Berglund (2012). "Respondent-driven sampling in a syringe exchange setting." Scandinavian journal of public health 40(8): 725-729.
356. Heimer, R., N. Dasgupta, K. S. Irwin, M. Kinzly, A. P. Harvey, A. Givens and L. E. Grau (2012). "Chronic pain, Addiction severity, and misuse of opioids in Cumberland County, Maine." Addictive Behaviors 37(3): 346-349.
357. Hequembourg, A., J. Livingston and K. Parks (2012). "Lifetime sexual victimization risks among sexual minority men." Alcoholism: Clinical and Experimental Research 36: 300A.
358. Hladik, W., J. Barker, J. M. Ssenkusu, A. Opio, J. W. Tappero, A. Hakim and D. Serwadda (2012). "HIV infection among men who have sex with men in kampala, uganda-a respondent driven sampling survey." PLoS ONE 7(5).
359. Iskandar, S., C. A. J. de Jong, T. Hidayat, I. M. P. Siregar, T. H. Achmad, R. van Crevel and A. van der Ven (2012). "Successful testing and treating of HIV/AIDS in Indonesia depends on the addiction treatment modality." Journal of Multidisciplinary Healthcare 5: 329-336.
360. Johnston, L. G., G. Paz-Bailey, S. Morales-Miranda, M. Morgan, B. Alvarez, L. Hickman and E. Monterroso (2012). "High prevalence of Mycoplasma genitalium among female sex workers in honduras: Implications for the spread of HIV and other sexually transmitted infections." International Journal of STD and AIDS 23(1): 5-11.
361. Jonas, A. B., A. M. Young, C. B. Oser, C. G. Leukefeld and J. R. Havens (2012). "OxyContin as currency: OxyContin use and increased social capital among rural Appalachian drug users." Social Science and Medicine 74(10): 1602-1609.
362. Jung, M. (2012). "Immigrant workers' knowledge of HIV/AIDS and their sexual risk behaviors: A respondent-driven sampling survey in South Korea." Sexuality and Disability 30(2): 199-208.
363. Kim, E. J., J. Creswell, M. E. Guardado, N. Shah, A. A. Kim, A. I. Nieto, F. de Maria Hernandez-Ayala, E. Monterroso and G. Paz-Bailey (2012). "Correlates of bisexual behaviors among men who have sex with men in El Salvador." AIDS and Behavior 17(4): 1279-1287.
364. Kolahi, A. A., A. Sayyarifard, A. Rastegarpour, M. R. Sohrabi, A. Abadi and M. Nabavi (2012). "The function of vulnerable and at-risk women in prevention of HIV/AIDS. [Persian]." Qom University of Medical Sciences Journal 6(2): e58-Pe64.
365. Lansky, A., A. Drake, C. Wejnert, H. Pham, M. Cribbin and D. D. Heckathorn (2012). "Assessing the assumptions of respondent-driven sampling in the national HIV behavioral surveillance system among injecting drug users." Open AIDS Journal 6(SPEC.ISSUE 1): 77-82.
366. Lauby, J. L., G. Marks, T. Bingham, K. L. Liu, A. Liau, A. Stueve and G. A. Millett (2012). "Having supportive social relationships is associated with reduced risk of unrecognized HIV infection among black and Latino men who have sex with men." AIDS and behavior 16(3): 508-515.
367. Li, J., X. S. Chen, M. G. Merli, S. S. Weir and G. E. Henderson (2012). "Systematic differences in risk behaviors and syphilis prevalence across types of female sex workers: A preliminary study in Liuzhou, China." Sexually Transmitted Diseases 39(3): 195-200.
368. Li, X., H. Lu, H. F. Raymond, Y. Sun, Y. Jia, X. He, S. Fan, Y. Shao, W. McFarland, Y. Xiao and Y. Ruan (2012). "Untested and undiagnosed: Barriers to HIV testing among men who have sex with men, Beijing, China." Sexually Transmitted Infections 88(3): 187-193.
369. Liao, M., X. Nie, R. Pan, C. Wang, S. Ruan, C. Zhang, D. Kang, J. Fu, Y. Qian, X. Tao and J. Zhao (2012). "Consistently low prevalence of syphilis among female sex workers in Jinan, China: Findings from two consecutive respondent driven sampling surveys." PLoS ONE 7(4).
370. Lin, P., M. Wang, Y. Li, Q. Zhang, F. Yang and J. Zhao (2012). "Detoxification center-based sampling missed a subgroup of higher risk drug users, a case from Guangdong, China." PLoS ONE 7(4).
371. Ma, W., H. F. Raymond, E. C. Wilson, W. McFarland, H. Lu, X. Ding, R. Lu, X. Ma, D. Xia, J. Xu, X. He, L. Feng, S. Fan, X. Li, J. Sun, Y. Jia, Y. Shao, Y. Ruan and Y. Xiao (2012). "Participation of HIV prevention programs among men who have sex with men in two cities of China - a mixed method study." BMC Public Health 12(847).
372. Mackesy-Amiti, M. E., G. R. Donenberg and L. J. Ouellet (2012). "Prevalence of psychiatric disorders among young injection drug users." Drug and Alcohol Dependence 124(1-2): 70-78.
373. McCreesh, N., S. D. W. Frost, J. Seeley, J. Katongole, M. N. Tarsh, R. Ndunguse, F. Jichi, N. L. Lunel, D. Maher, L. G. Johnston, P. Sonnenberg, A. J. Copas, R. J. Hayes and R. G. White (2012). "Evaluation of respondent-driven sampling." Epidemiology 23(1): 138-147.
374. Medhi, G. K., M. Jagadish, M. Kermode, R. S. Paranjape, R. Adhikary, S. K. Phukan and P. Ngully (2012). "Factors associated with history of drug use among female sex workers (FSW) in a high HIV prevalence state of India." BMC Public Health 12(273).
375. Medhi, G. K., J. Mahanta, R. S. Paranjape, R. Adhikary, S. G. Singh, S. Brogen Akoijam and P. Goswami (2012). "Factors associated with ever HIV testing among injecting drug users (IDUs) in two HIV high prevalent States of India." Indian Journal of Medical Research, Supplement 136(SUPPL): 64-71.
376. Milkman, R., A. L. Gonz√°lez and P. Ikeler (2012). "Wage and hour violations in urban labour markets: a comparison of Los Angeles, New York and Chicago." Industrial Relations Journal 43(5): 378-398.
377. Mills, H. L., C. Colijn, P. Vickerman, D. Leslie, V. Hope and M. Hickman (2012). "Respondent driven sampling and community structure in a population of injecting drug users, Bristol, UK." Drug and Alcohol Dependence 126(3): 324-332.
378. Mizuno, Y., C. Borkowf, G. A. Millett, T. Bingham, G. Ayala and A. Stueve (2012). "Homophobia and racism experienced by Latino men who have sex with men in the United States: correlates of exposure and associations with HIV risk behaviors." AIDS and behavior 16(3): 724-735.
379. Montealegre, J. R., J. M. Risser, B. J. Selwyn, K. Sabin and S. A. McCurdy (2012). "HIV testing behaviors among undocumented Central American immigrant women in Houston, Texas." Journal of immigrant and minority health / Center for Minority Public Health 14(1): 116-123.
380. Morineau, G., L. J. M. Bollen, R. I. Syafitri, N. Nurjannah, D. E. Mustikawati and R. Magnani (2012). "HIV prevalence and risk behaviours among injecting drug users in six indonesian cities implications for future HIV prevention programs." Harm Reduction Journal 9(37).
381. Navadeh, S., A. Mirzazadeh, L. Mousavi, A. A. Haghdoost, N. Fahimfar and A. Sedaghat (2012). "HIV, HSV2 and syphilis prevalence in female sex workers in Kerman, South-East Iran; using respondent-driven sampling." Iranian Journal of Public Health 41(12): 60-65.
382. Nehl, E. J., F. Y. Wong, N. He, Z. J. Huang and T. Zheng (2012). "Prevalence and correlates of alcohol use among a sample of general MSM and money boys in Shanghai, China." AIDS care 24(3): 324-330.
383. Oteo Perez, A., A. Benschop and D. J. Korf (2012). "Differential profiles of crack users in respondent-driven and institutional samples: A three-site comparison." European Addiction Research 18(4): 184-192.
384. Pando, M. A., I. C. Balan, R. Marone, C. Dolezal, C. Leu, L. Squiquera, V. Barreda, M. Rodriguez Fermepin, L. Gallo Vaulet, J. Rey, M. Picconi, A. Carballo-Dieguez and M. M. Avila (2012). "HIV and other sexually transmitted infections among men who have sex with men recruited by RDS in Buenos Aires, Argentina: high HIV and HPV infection." PLoS ONE 7(6).
385. Purnima, B., M. Jagadish, G. K. Medhi, D. Jayesh, R. S. Paranjape and T. Gay (2012). "Sexual activity as risk factor for hepatitis C virus (HCV) transmission among the female sex workers in Nagaland." Indian Journal of Medical Research 136(7 Suppl): 30-35.
386. Qiu, P., Y. Yang, X. Ma, F. Wu, P. Yuan, Q. Liu and E. Caine (2012). "Respondent-driven sampling to recruit in-country migrant workers in China: a methodological assessment." Scandinavian journal of public health 40(1): 92-101.
387. Reed, E., J. Gupta, M. Biradavolu and K. M. Blankenship (2012). "Migration/mobility and risk factors for HIV among female sex workers in Andhra Pradesh, India: Implications for HIV prevention." International Journal of STD and AIDS 23(4): e7-e13.
388. Reisner, S., M. Mimiaga, S. E. Bland, M. A. Driscoll, K. Cranston and K. H. Mayer (2012). "Pathways to embodiment of HIV risk: Black men who have sex with transgender partners, Boston, Massachusetts." AIDS Education and Prevention 24(1): 15-26.
389. Rhodes, S. D., T. P. McCoy, K. C. Hergenrather, A. T. Vissman, M. Wolfson, J. Alonzo, F. R. Bloom, J. Alegria-Ortega and E. Eng (2012). "Prevalence estimates of health risk behaviors of immigrant latino men who have sex with men." Journal of Rural Health 28(1): 73-83.
390. Rosa, M. d. l., R. Babino, A. Rosario, N. V. Martinez and L. Aijaz (2012). "Challenges and strategies in recruiting, interviewing, and retaining recent Latino immigrants in substance abuse and HIV epidemiologic studies." American Journal of Addictions 21(1): 11-22.
391. Sanchez, T., A. Smith, D. Denson, E. DiNenno and A. Lansky (2012). "Developing a web-based HIV behavioral surveillance pilot project among men who have sex with men." Open AIDS Journal 6(SPEC.ISSUE 1): 224-231.
392. Sarin, E. and D. Kerrigan (2012). "The impact of human rights violations and perceptions of discrimination on health service utilization among injection drug users in Delhi, India." Substance use & misuse 47(3): 230-243.
393. Sarna, A., W. Tun, A. Bhattacharya, D. Lewis, Y. S. Singh and L. Apicella (2012). "Assessment of unsafe injection practices and sexual behaviors among male injecting drug users in two urban cities of India using respondent driven sampling." Southeast Asian Journal of Tropical Medicine and Public Health 43(3): 652-667.
394. Schneider, J., S. Michaels and A. Bouris (2012). "Family network proportion and HIV risk among black men who have sex with men." Journal of acquired immune deficiency syndromes (1999) 61(5): 627-635.
395. Schneider, J. A., T. Walsh, B. Cornwell, D. Ostrow, S. Michaels and E. O. Laumann (2012). "HIV health center affiliation networks of black men who have sex with men: Disentangling fragmented patterns of HIV prevention service utilization." Sexually Transmitted Diseases 39(8): 598-604.
396. Silva-Santisteban, A., H. F. Raymond, X. Salazar, J. Villayzan, S. Leon, W. McFarland and C. F. Caceres (2012). "Understanding the HIV/AIDS epidemic in transgender women of Lima, Peru: results from a sero-epidemiologic study using respondent driven sampling." AIDS and Behavior 16(4): 872-881.
397. Stoddard, S. A., J. A. Bauermeister, D. Gordon-Messer, M. Johns and M. A. Zimmerman (2012). "Permissive norms and young adults' alcohol and marijuana use: The role of online communities." Journal of Studies on Alcohol and Drugs 73(6): 968-975.
398. Stulhofer, A., A. Chetty, R. A. Rabie, I. Jwehan and A. Ramlawi (2012). "The prevalence of HIV, HBV, HCV, and HIV-related risk-taking behaviors among Palestinian injecting drug users in the East Jerusalem Governorate." Journal of urban health : bulletin of the New York Academy of Medicine 89(4): 671-676.
399. Sucheta, D., K. Anjalee, R. Sunil, M. Mandar, K. Shirin, P. Dilip, D. Jayesh, A. Shailaja, P. Narayan, M. Uma and P. Ramesh (2012). "Recruitment of at-risk population through network based sampling: experiences from Maharashtra IBBA Round-I survey." Indian Journal of Medical Research 136(7 Suppl): 36-43.
400. Tun, W., S. Kellerman, S. Maimane, Z. Fipaza, M. Sheehy, L. Vu and D. Nel (2012). "HIV-related conspiracy beliefs and its relationships with HIV testing and unprotected sex among men who have sex with men in Tshwane (Pretoria), South Africa." AIDS Care - Psychological and Socio-Medical Aspects of AIDS/HIV 24(4): 459-467.
401. Villanti, A., D. German, F. Sifakis, C. Flynn and D. Holtgrave (2012). "Smoking, HIV status, and HIV risk behaviors in a respondent-driven sample of injection drug users in Baltimore, Maryland: The besure study." AIDS Education and Prevention 24(2): 132-147.
402. Vu, L., W. Tun, M. Sheehy and D. Nel (2012). "Levels and correlates of internalized homophobia among men who have sex with men in Pretoria, South Africa." AIDS and behavior 16(3): 717-723.
403. Wang, Y., J. Xu, Z. Li, G. Zhang, L. Li, J. Fan, S. Jia, H. Yang and W. Yao (2012). "Analysis on HIV/syphilis infection with Queue baseline of MSM and the influencing factors in Mianyang City. [Chinese]." Chinese Journal of Dermatovenereology 26(5): 410-414.
404. Wang, Y., G. Zhang and J. Li (2012). "Survey of syphilis infections among MSM in Mianyang and the influencing factors. [Chinese]." China Tropical Medicine 12(7): 798-802.
405. Wang, Y., G. Zhang, L. Li, J. Fan, X. Zhao, S. Jia, H. Yang and W. Yao (2012). "HIV infection and influencing factors in MSM (men having sex with men) population in Mianyang, China. [Chinese]." Chinese Journal of Viral Diseases 2(4): 271-276.
406. Wang, Y., G. Zhang, L. Li, J. Fan, X. Zhao, S. Jia, H. Yang and W. Yao (2012). "Analysis on the new HIV/syphilis infections of MSM in Mianyang and the influencing factors. [Chinese]." Chinese Journal of Dermatovenereology 26(6): 509-512.
407. Wangroongsarb, P. (2012). "Respondent-driven sampling on the thailandcambodia border. i. can malaria cases be contained in mobile migrant workers?" American Journal of Tropical Medicine and Hygiene 1): 110.
408. Wei, C., W. McFarland, G. N. Colfax, V. Fuqua and H. F. Raymond (2012). "Reaching black men who have sex with men: A comparison between respondent-driven sampling and time-location sampling." Sexually Transmitted Infections 88(8): 622-626.
409. Weir, S. S., M. G. Merli, J. Li, A. D. Gandhi, W. W. Neely, J. K. Edwards, C. M. Suchindran, G. E. Henderson and X. S. Chen (2012). "A comparison of respondent-driven and venue-based sampling of female sex workers in Liuzhou, China." Sexually transmitted infections 88 Suppl 2: i95-101.
410. Young, A. M. and J. R. Havens (2012). "Transition from first illicit drug use to first injection drug use among rural Appalachian drug users: a cross-sectional comparison and retrospective survival analysis." Addiction (Abingdon, England) 107(3): 587-596.
411. Zembe, Y. Z., L. Townsend, A. Thorson and A. M. Ekstrom (2012). "Predictors of inconsistent condom use among a hard to reach population of young women with multiple sexual partners in peri-urban South Africa." PLoS ONE 7(12).
412. Zhang, L., X. Ding, R. Lu, L. Feng, X. Li, Y. Xiao, Y. Ruan, S. H. Vermund, Y. Shao and H. Z. Qian (2012). "Predictors of hiv and syphilis among men who have sex with men in a chinese metropolitan city: Comparison of risks among students and non-students." PLoS ONE 7(5).
413. Arfken, C. L., S. Ahmed and W. Abu-Ras (2013). "Respondent-driven sampling of Muslim undergraduate U.S. college students and alcohol use: pilot study." Social Psychiatry and Psychiatric Epidemiology 48(6): 945-953.
414. Armstrong, G., G. K. Medhi, M. Kermode, M. Jagadish, G. Prabuddhagopal and R. S. Paranjape (2013). "Exposure to HIV prevention programmes associated with improved condom use and uptake of HIV testing by female sex workers in Nagaland, Northeast India." BMC Public Health 13(476).
415. Arney, J., R. L. Street Jr and A. D. Naik (2013). "Consumers' various and surprising responses to direct-to-consumer advertisements in magazine print." Patient Preference and Adherence 7: 95-102.
416. Bacak, V., D. Lausevic, B. Mugosa, Z. Vratnica and N. Terzic (2013). "Hepatitis C virus infection and related risk factors among injection drug users in montenegro." European Addiction Research 19(2): 68-73.
417. Bernat, D., J. Bauermeister, K. Chevallier, M. Gerend and M. Zimmerman (2013). "Young adult initiation of the HPV vaccine: Results from a national survey of men and women." Journal of Adolescent Health 1): S31.
418. Bernhardt, A., M. W. Spiller and D. Polson (2013). "All work and no pay: Violations of employment and labor laws in Chicago, Los Angeles and New York City." Social forces 91(3): 725-746.
419. Borders, T. F. and B. M. Booth (2013). "Stimulant use, religiosity, and the odds of developing or maintaining an alcohol use disorder over time." Journal of Studies on Alcohol and Drugs 74(3): 369-377.
420. Dennis, A. M., W. Murillo, F. De Maria Hernandez, M. E. Guardado, A. I. Nieto, I. Lorenzana De Rivera, J. J. Eron and G. Paz-Bailey (2013). "Social network-based recruitment successfully reveals HIV-1 transmission networks among high-risk individuals in El Salvador." Journal of Acquired Immune Deficiency Syndromes 63(1): 135-141.
421. Djonic, D., M. Djuric, F. Bassioni-Stamenic, W. McFarland, T. Knezevic, S. Nikolic, V. Zivkovic and S. Vallabhaneni (2013). "HIV-related risk behaviors among roma youth in Serbia: Results of two community-based surveys." Journal of Adolescent Health 52(2): 234-240.
422. Dunkle, K. L., F. Y. Wong, E. J. Nehl, L. Lin, N. He, J. Huang and T. Zheng (2013). "Male-on-male intimate partner violence and sexual risk behaviors among money boys and other men who have sex with men in Shanghai, China." Sexually Transmitted Diseases 40(5): 362-365.
423. Eluwa, G. I., S. A. Strathdee, S. B. Adebayo, B. Ahonsi and S. B. Adebajo (2013). "A profile on HIV prevalence and risk behaviors among injecting drug users in Nigeria: Should we be alarmed?" Drug and Alcohol Dependence 127(1-3): 65-71.
424. Eritsyan, K. U., O. S. Levina, E. White, T. T. Smolskaya and R. Heimer (2013). "HIV prevalence and risk behavior among injection drug users and their sex partners in two Russian cities." AIDS Research and Human Retroviruses 29(4): 687-690.
425. Eritsyan, K. U., O. S. Levina, E. White, T. T. Smolskaya and R. Heimer (2013). "Short communication: HIV prevalence and risk behavior among injection drug users and their sex partners in two Russian cities." AIDS Research and Human Retroviruses 29(4): 687-690.
426. Feng, Y., Z. Wang and L. Wang (2013). "The investigation of HIV knowledge and high risky behavior state among men who have sex with men in Qinhuangdao city. [Chinese]." Chinese Journal of Dermatovenereology 27(3): 271-273.
427. Garfein, R. S., A. Rondinelli, R. F. W. Barnes, J. Cuevas, M. Metzner, M. Velasquez, D. Rodriguez, M. Reilly, J. Xing and E. H. Teshale (2013). "HCV infection prevalence lower than expected among 18-40-year-old injection drug users in San Diego, CA." Journal of Urban Health: Bulletin of the New York Academy of Medicine 90(3): 516-528.
428. Gordon-Messer, D., J. A. Bauermeister, A. Grodzinski and M. Zimmerman (2013). "Sexting among young adults." Journal of Adolescent Health 52(3): 301-306.
429. Horyniak, D., P. Higgs, R. Jenkinson, L. Degenhardt, M. Stoove, T. Kerr, M. Hickman, C. Aitken and P. Dietze (2013). "Establishing the Melbourne injecting drug user cohort study (MIX): Rationale, methods, and baseline and twelve-month follow-up results." Harm Reduction Journal 10(1).
430. Jiang, C., J. Na, N. Li, W. Diao, Y. Gu, L. Zhao, Y. Zou, Y. Chen, L. Liu, H. Mu, Y. Liu, L. Yu, X. Yang and G. Pan (2013). "Elevated 12-Month and Lifetime Prevalence and Comorbidity Rates of Mood, Anxiety, and Alcohol Use Disorders in Chinese Men Who Have Sex with Men." PLoS ONE 8(4).
431. Johnston, L., T. Caballero, Y. Dolores and H. Vales (2013). "HIV, Hepatitis B/C and syphilis prevalence and risk behaviors among gay/trans/men who have sex with men, Dominican Republic." Int J STD AIDS 24(4): 313-321.
432. Johnston, L. G., K. Alami, M. H. El Rhilani, M. Karkouri, O. Mellouk, A. Abadie, N. Rafif, L. Ouarsas, A. Bennani and B. El Omari (2013). "HIV, syphilis and sexual risk behaviours among men who have sex with men in Agadir and Marrakesh, Morocco." Sexually transmitted infections.
433. Johnston, L. G. and S. Corceal (2013). "Unexpectedly high injection drug use, HIV and hepatitis C prevalence among female sex workers in the Republic of Mauritius." AIDS and Behavior 17(2): 574-584.
434. Kerr, L. R. F. S., R. S. Mota, C. Kendall, A. D. A. Pinho, M. B. Mello, M. D. C. Guimaraes, I. Dourado, A. M. De Brito, A. Benzaken, W. McFarland and G. Rutherford (2013). "HIV among MSM in a large middle-income country." Aids 27(3): 427-435.
435. Lin, P., M. Wang, Y. Li and Q. Zhang (2013). "Comparison of HIV prevalence and its correlates between the community-based and the detoxification center-based drug users. [Chinese]." Modern Preventive Medicine 40(3): 463-466.
436. Lung, V., S. Adebajo, W. Tun, M. Sheehy, A. Karlyn, J. Njab, A. Azeez and B. Ahonsi (2013). "High HIV prevalence among men who have sex with men in Nigeria: implications for combination prevention." JAIDS, Journal of Acquired Immune Deficiency Syndromes 63(2): 221-227.
437. Magnus, M., I. Kuo, G. Phillips, II, A. Rawls, J. Peterson, L. Montanez, T. West-Ojo, Y. J. Jia, J. Opoku, N. Kamanu-Elias, F. Hamilton, A. Wood and A. E. Greenberg (2013). "Differing HIV risks and prevention needs among men and women injection drug users (IDU) in the District of Columbia." Journal of Urban Health: Bulletin of the New York Academy of Medicine 90(1): 157-166.
438. Manopaiboon, C., D. Prybylski, W. Subhachaturas, S. Tanpradech, O. Suksripanich, U. Siangphoe, L. G. Johnston, P. Akarasewi, A. Anand, K. K. Fox and S. J. Whitehead (2013). "Unexpectedly high HIV prevalence among female sex workers in Bangkok, Thailand in a respondent-driven sampling survey." International Journal of STD and AIDS 24(1): 34-38.
439. Martins, T. A., L. R. F. S. Kerr, R. H. M. Macena, R. S. Mota, K. L. Carneiro, R. C. Gondim and C. Kendall (2013). "Travestis, an unexplored population at risk of HIV in a large metropolis of northeast Brazil: a respondent-driven sampling survey." AIDS Care 25(5): 606-612.
440. McCoy, S. I., K. Shiu, T. E. Martz, C. D. Smith, L. Mattox, D. R. Gluth, N. Murgai, M. Martin and N. S. Padian (2013). "Improving the efficiency of HIV testing with peer recruitment, financial incentives, and the involvement of persons living with HIV infection." Journal of acquired immune deficiency syndromes (1999) 63(2): e56-63.
441. Mirzoyan, L., S. Berendes, C. Jeffery, J. Thomson, H. B. Othman, L. Danon, A. A. Turki, R. Saffialden and J. J. Valadez (2013). "New evidence on the HIV epidemic in Libya: Why countries must implement prevention programs among people who inject drugs." Journal of Acquired Immune Deficiency Syndromes 62(5): 577-583.
442. Mishra, S. R. and V. Khanal (2013). "Sexual behaviors among men who have sex with men: A quantitative cross sectional study in Kathmandu Valley, Nepal." HIV/AIDS - Research and Palliative Care 5: 81-88.
443. Nyoni, J. E. and M. W. Ross (2013). "Condom use and HIV-related behaviors in urban Tanzanian men who have sex with men: A study of beliefs, HIV knowledge sources, partner interactions and risk behaviors." AIDS Care - Psychological and Socio-Medical Aspects of AIDS/HIV 25(2): 223-229.
444. Perez, A. O., M. J. L. F. Cruyff, A. Benschop and D. J. Korf (2013). "Estimating the prevalence of crack dependence using capture-recapture with institutional and field data: a three-city study in the Netherlands." Substance Use and Misuse 48(1/2): 173-180.
445. Rudolph, A. E., N. D. Crawford, C. Latkin, J. H. Fowler and C. M. Fuller (2013). "Individual and neighborhood correlates of membership in drug using networks with a higher prevalence of HIV in New York City (2006-2009)." Annals of Epidemiology 23(5): 267-274.
446. Saw, Y. M., J. Yasuoka, T. N. Saw, K. C. Poudel, S. Tun and M. Jimba (2013). "What are the factors associated with HIV testing among male injecting and non-injecting drug users in Lashio, Myanmar: A cross-sectional study." BMJ Open 3(6).
447. Silva-Santisteban, A., E. R. Segura, C. Sandoval, M. Giron, M. Petrera and C. F. Caceres (2013). "Social and economic determinants of unequal HIV care access among people living with HIV in Peru." Globalization and Health 9(22).
448. Sordo, L., S. Perez-Vicente, M. M. Rodriguez Del Aguila and M. J. Bravo (2013). "Respondent-driven sampling for the study of difficult access populations. [Muestreo dirigido por los participantes para el estudio de poblaciones de dificil acceso." Medicina Clinica 140(2): 83-87.
449. Stephens, D. B. and J. R. Havens (2013). "Predictors of alcohol use among rural drug users after disclosure of hepatitis C virus status." Journal of Studies on Alcohol and Drugs 74(3): 386-395.
450. Townsend, L., Y. Zembe, C. Mathews and A. J. Mason-Jones (2013). "Estimating HIV prevalence and HIV-related risk behaviors among heterosexual women who have multiple sex partners using respondent-driven sampling in a high-risk community in South Africa." Journal of Acquired Immune Deficiency Syndromes 62(4): 457-464.
451. Valadez, J. J., S. Berendes, C. Jeffery, J. Thomson, H. Ben Othman, L. Danon, A. A. Turki, R. Saffialden and L. Mirzoyan (2013). "Filling the Knowledge Gap: Measuring HIV Prevalence and Risk Factors among Men Who Have Sex with Men and Female Sex Workers in Tripoli, Libya." PLoS ONE 8(6).
452. Vandenhoudt, H. M., L. Langat, J. Menten, F. Odongo, S. Oswago, G. Luttah, C. Zeh, T. Crucitti, K. Laserson, J. Vulule and A. Buve (2013). "Prevalence of HIV and other sexually transmitted infections among female sex workers in Kisumu, Western Kenya, 1997 and 2008." PLoS ONE 8(1).
453. Vorobjov, S., D. C. Des Jarlais, K. Abel-Ollo, A. Talu, K. Ruutel and A. Uuskula (2013). "Socio-demographic factors, health risks and harms associated with early initiation of injection among people who inject drugs in Tallinn, Estonia: Evidence from cross-sectional surveys." International Journal of Drug Policy 24(2): 150-155.
454. Vu, L., S. Adebajo, W. Tun, M. Sheehy, A. Karlyn, J. Njab, A. Azeez and B. Ahonsi (2013). "High HIV prevalence among men who have sex with men in Nigeria: implications for combination prevention." Journal of acquired immune deficiency syndromes (1999) 63(2): 221-227.
455. Wang, Y., J. Xu, Z. Li, G. Zhang, L. Li and J. Fan (2013). "Features and the influencing factors of the sexual behaviors in last 6 months among MSM in Mianyang City. [Chinese]." Modern Preventive Medicine 40(1): 64-69.
456. Yam, E. A., Z. Mnisi, B. Sithole, C. Kennedy, D. L. Kerrigan, A. O. Tsui and S. Baral (2013). "Association between condom use and use of other contraceptive methods among female sex workers in swaziland: A relationship-level analysis of condom and contraceptive use." Sexually Transmitted Diseases 40(5): 406-412.
457. Yu, L., C. Jiang, J. Na, N. Li, W. Diao, Y. Gu, L. Zhao, Y. Zou, Y. Chen, L. Liu, H. Mu, Y. Liu, L. Yu, X. Yang and G. Pan (2013). "Elevated 12-month and lifetime prevalence and comorbidity rates of mood, anxiety, and alcohol use disorders in Chinese men who have sex with men." PLoS ONE 8(4).
458. Zhang, L., Y. Xiao, R. Lu, G. Wu, X. Ding, H. Z. Qian, W. McFarland, Y. Ruan, S. H. Vermund and Y. Shao (2013). "Predictors of HIV testing among men who have sex with men in a large Chinese City." Sexually Transmitted Diseases 40(3): 235-240.
459. Zhang, R., Q. Liu, Y. Yang, S. Zhang, F. He, S. Guo and H. Zhou (2013). "Research on influencing factors of floating women's reproductive health-related behaviors. [Chinese]." Modern Preventive Medicine 40(1): 190-193.
460. Zohrabyan, L., L. Johnston, O. Scutelniciuc, A. Iovita, L. Todirascu, T. Costin, V. Plesca, T. Cotelnic-Harea and G. Ionascu (2013). "HIV, hepatitis and syphilis prevalence and correlates of condom use during anal sex among men who have sex with men in the Republic of Moldova." International Journal of STD & AIDS 24(5): 357-364.
461. Zohrabyan, L., L. G. Johnston, O. Scutelniciuc, A. Iovita, L. Todirascu, T. Costin, V. Plesca, T. Cotelnic-Harea and G. Ionascu (2013). "Determinants of HIV Infection Among Female Sex Workers in Two Cities in the Republic of Moldova: The Role of Injection Drug Use and Sexual Risk." AIDS and Behavior: 1-9.

**SECTION 5: LIST OF EXCLUDED STUDIES – METHODOLOGICAL PAPERS**

1. Wang, J., R. G. Carlson, R. S. Falck, H. A. Siegal, A. Rahman and L. Li (2005). "Respondent-driven sampling to recruit MDMA users: A methodological assessment." Drug and Alcohol Dependence 78(2): 147-157.
2. Salganik, M. J. (2006). "Variance estimation, design effects, and sample size calculations for respondent-driven sampling." Journal of Urban Health 83(1 SUPPL.): i98-i112.
3. Tiffany, J. S. (2006). "Respondent-driven sampling in participatory research contexts: Participant-driven recruitment." Journal of Urban Health 83(1 SUPPL.): i113-i124.
4. Kendall, C., L. R. F. S. Kerr, R. C. Gondim, G. L. Werneck, R. H. M. Macena, M. K. Pontes, L. G. Johnston, K. Sabin and W. McFarland (2008). "An empirical comparison of respondent-driven sampling, time location sampling, and snowball sampling for behavioral surveillance in men who have sex with men, Fortaleza, Brazil." AIDS and Behavior 12(SUPPL. 1): S97-S104.
5. Ompad, D. C., S. Galea, G. Marshall, C. M. Fuller, L. Weiss, J. R. Beard, C. Chan, V. Edwards and D. Vlahov (2008). "Sampling and recruitment in multilevel studies among marginalized urban populations: The IMPACT studies." Journal of Urban Health 85(2): 268-280.
6. Goel, S. and M. J. Salganik (2009). "Respondent-driven sampling as Markov chain Monte Carlo." Statistics in Medicine 28(17): 2202-2229.
7. Poon, A. F., K. C. Brouwer, S. A. Strathdee, M. Firestone-Cruz, R. M. Lozada, S. L. Pond, D. D. Heckathorn and S. D. Frost (2009). "Parsing social network survey data from hidden populations using stochastic context-free grammars." PloS one 4(9): e6777.
8. Goel, S. and M. J. Salganik (2010). "Assessing respondent-driven sampling." Proceedings of the National Academy of Sciences of the United States of America 107(15): 6743-6747.
9. Barbosa Jr, A., A. R. P. Pascom, C. L. Szwarcwald, C. Kendall and W. McFarland (2011). "Transfer of sampling methods for studies on most-at-risk populations (MARPs) in Brazil. [Portuguese]

Transferencia de metodos de amostragem para estudos em populacoes sob maior risco a infeccao pelo HIV no Brasil." Cadernos de Saude Publica 27(SUPPL. 1): S36-S44.

1. Bauer, G. R. and N. Khobzi (2011). "Scaling up respondent-driven sampling beyond the local level: Implementation lessons from the trans pulse project." American Journal of Epidemiology 173: S110.
2. Burlew, K., S. Larios, L. Suarez-Morales, B. Holmes, K. Venner and R. Chavez (2011). "Increasing Ethnic Minority Participation in Substance Abuse Clinical Trials: Lessons Learned in the National Institute on Drug Abuse's Clinical Trials Network." Cultural Diversity and Ethnic Minority Psychology 17(4): 345-356.
3. Frost, S. (2011). "Respondent-driven sampling: Uses, assumptions, limits and prospects." Sexually Transmitted Infections 87: A15.
4. Goel, S. (2011). "Assessing respondent-driven sampling." Sexually Transmitted Infections 87: A15.
5. Szwarcwald, C. L., P. R. B. De Souza Junior, G. N. Damacena, A. B. Junior and C. Kendall (2011). "Analysis of data collected by RDS among sex workers in 10 Brazilian cities, 2009: Estimation of the prevalence of HIV, variance, and design effect." Journal of Acquired Immune Deficiency Syndromes 57(SUPPL. 3): S129-S135.
6. Toledo, L., C. T. Codeco, N. Bertoni, E. Albuquerque, M. Malta and F. I. Bastos (2011). "Putting respondent-driven sampling on the map: Insights from Rio de Janeiro, Brazil." Journal of Acquired Immune Deficiency Syndromes 57(SUPPL. 3): S136-S143.
7. Pylli, M. and V. Raftopoulos (2012). "Description of the sampling research methods used for the hard to reach population in the surveillance of HIV-infection." Archives of Hellenic Medicine 29(6): 737-743.
8. Zheng, L. and Y. Zheng (2012). "Efficacy of human immunodeficiency virus prevention interventions among men who have sex with men in China: A meta-analysis." Sexually Transmitted Diseases 39(11): 886-893.
9. Johnston, L. G., D. Prybylski, H. F. Raymond, A. Mirzazadeh, C. Manopaiboon and W. McFarland (2013). "Incorporating the service multiplier method in respondent-driven sampling surveys to estimate the size of hidden and hard-to-reach populations: Case studies from around the world." Sexually Transmitted Diseases 40(4): 304-310.
10. Rondy, M., L. Wiessing, S. J. Hutchinson, C. Mathei, F. Mathis, V. Mravcik, L. Norden, M. Rosinska, O. Scutelniciuc, B. Suligoi, F. Vallejo, M. Van Veen and M. Kretzschmar (2013). "Hepatitis C prevalence in injecting drug users in Europe, 1990-2007: Impact of study recruitment setting." Epidemiology and Infection 141(3): 563-572.
11. Yan, B. and S. Gregory (2013). "Identifying Communities and Key Vertices by Reconstructing Networks from Samples." PLoS ONE 8(4).

**SECTION 6: LIST OF EXCLUDED STUDIES - REVIEWS**

1. Des Jarlais, D. C., K. Arasteh, S. Semaan and E. Wood (2009). "HIV among injecting drug users: current epidemiology, biologic markers, respondent-driven sampling, and supervised-injection facilities." Current opinion in HIV and AIDS **4**(4): 308-313.
2. Diaz, T., J. M. Garcia-Calleja, P. D. Ghys and K. Sabin (2009). "Advances and future directions in HIV surveillance in low- and middle-income countries." Current opinion in HIV and AIDS **4**(4): 253-259.
3. Johnston, L. G., M. Malekinejad, C. Kendall, I. M. Iuppa and G. W. Rutherford (2008). "Implementation challenges to using respondent-driven sampling methodology for HIV biological and behavioral surveillance: Field experiences in international settings." AIDS and Behavior **12**(SUPPL. 1): S131-S141.
4. Magnani, R., K. Sabin, T. Saidel and D. Heckathorn (2005). "Review of sampling hard-to-reach and hidden populations for HIV surveillance." Aids **19**(SUPPL. 2): S67-S72.
5. Malekinejad, M., L. G. Johnston, C. Kendall, L. R. F. S. Kerr, M. R. Rifkin and G. W. Rutherford (2008). "Using respondent-driven sampling methodology for HIV biological and behavioral surveillance in international settings: A systematic review." AIDS and Behavior **12**(SUPPL. 1): S105-S130.
6. Nomura, Y., K. C. Poudel and M. Jimba (2007). "Hard-to-reach populations in Japan." Southeast Asian Journal of Tropical Medicine and Public Health **38**(2): 325-327.
7. Paquette, D. and J. De Wit (2010). "Sampling methods used in developed countries for behavioural surveillance among men who have sex with men." AIDS and behavior **14**(6): 1252-1264.
8. Semaan, S., J. Lauby and J. Liebman (2002). "Street and network sampling in evaluation studies of HIV risk-reduction interventions." AIDS Reviews **4**(4): 213-223.
9. Semaan, S., S. Santibanez, R. S. Garfein, D. D. Heckathorn and D. C. Des Jarlais (2009). "Ethical and regulatory considerations in HIV prevention studies employing respondent-driven sampling." International Journal of Drug Policy **20**(1): 14-27.

**SECTION 7: LIST OF EXCLUDED STUDIES - EDITORIALS**

1. Heimer, R. (2005). "Critical issues and further questions about respondent-driven sampling: Comment on Ramirez-Valles, et al. (2005)." AIDS and Behavior 9(4): 403-408.
2. Ramirez-Valles, J., D. D. Heckathorn, R. Vazquez, R. M. Diaz and R. T. Campbell (2005). "The fit between theory and data in respondent-driven sampling: Response to Heimer." AIDS and Behavior 9(4): 409-414.
3. Abdul-Quader, A. S., D. D. Heckathorn, K. Sabin and T. Saidel (2006). "Implementation and analysis of respondent driven sampling: Lessons learned from the field." Journal of Urban Health 83(1 SUPPL.): i1-i5.
4. Broadhead, R. S. (2008). "Notes on a cautionary (tall) tale about respondent-driven sampling: A critique of Scott's ethnography." International Journal of Drug Policy 19(3): 235-237.
5. Lansky, A. and T. D. Mastro (2008). "Using respondent-driven sampling for behavioural surveillance: Response to Scott." International Journal of Drug Policy 19(3): 241-243.
6. Ouellet, L. J. (2008). "Cautionary comments on an ethnographic tale gone wrong." International Journal of Drug Policy 19(3): 238-240.
7. Scott, G. (2008). "Responses on a cautionary tale concerning the ethics of using respondent-driven sampling to study injection drug users." International Journal of Drug Policy 19(3): 246-247.
8. Scott, G. (2008). ""They got their program, and I got mine": A cautionary tale concerning the ethical implications of using respondent-driven sampling to study injection drug users." International Journal of Drug Policy 19(1): 42-51.
9. Bengtsson, L. and A. Thorson (2010). "Global HIV surveillance among MSM: Is risk behavior seriously underestimated?" Aids 24(15): 2301-2303.
10. Constantine, M. (2010). "Disentangling methodologies: The ethics of traditional sampling methodologies, community-based participatory research, and respondent-driven sampling." American Journal of Bioethics 10(3): 22-24.
11. Semaan, S., D. D. Heckathorn, D. C. Des Jarlais and R. S. Garfein (2010). "Ethical considerations in surveys employing respondent-driven sampling." American journal of public health 100(4): 582-583; author reply 583-584.
12. Berchenko, Y. and S. D. W. Frost (2011). "Capture-recapture methods and respondent-driven sampling: Their potential and limitations." Sexually Transmitted Infections 87(4): 267-268.
13. Marcus, U., F. Hickson, P. Weatherburn, A. J. Schmidt and E. Network (2012). "Prevalence of HIV among MSM in Europe: comparison of self-reported diagnoses from a large scale internet survey and existing national estimates." BMC public health 12: 978.
14. Salganik, M. J. (2012). "Commentary: Respondent-driven sampling in the real world." Epidemiology 23(1): 148-150.
15. White, R. G., A. Lansky, S. Goel, D. Wilson, W. Hladik, A. Hakim and S. D. W. Frost (2012). "Respondent driven sampling - Where we are and where should we be going?" Sexually Transmitted Infections 88(6): 397-399.

1. Department of Infectious Disease Epidemiology, London School of Hygiene and Tropical Medicine, UK ([Richard.White@lshtm.ac.uk](mailto:Richard.White@lshtm.ac.uk); [kate.orroth@gmail.com](mailto:kate.orroth@gmail.com) ) [↑](#footnote-ref-2)
2. US Centers for Disease Control and Prevention. Atlanta, GA, USA ([hxv8@cdc.gov](mailto:hxv8@cdc.gov); [ajd9@cdc.gov](mailto:ajd9@cdc.gov); [wfh3@cdc.gov](mailto:wfh3@cdc.gov), [wfu3@cdc.gov](mailto:wfu3@cdc.gov) ) [↑](#footnote-ref-3)
3. Department of Sociology and Office of Population Research, Princeton University ([mjs3@princeton.edu](mailto:mjs3@princeton.edu)) [↑](#footnote-ref-4)
4. Department of Global Community Health and Behavioral Sciences, Tulane University School of Public Health and Tropical Medicine; Global Health Science, University of California San Francisco, USA ( [lsjohnston.global@gmail.com](mailto:lsjohnston.global@gmail.com)) [↑](#footnote-ref-5)
5. Departamento de Saude Comunitaria, Universidade Federal do Ceara, Fortaleza, Ceara, Brazil ([ligiakerr@gmail.com](mailto:ligiakerr@gmail.com)) [↑](#footnote-ref-6)
6. Department of Global Community Health and Behavioral Sciences, Tulane University School of Public Health and Tropical Medicine ([ckendall@tulane.edu](mailto:ckendall@tulane.edu)) [↑](#footnote-ref-7)
7. World Bank, USA ([dwilson@worldbank.org](mailto:dwilson@worldbank.org) ) [↑](#footnote-ref-8)
8. Institute of Social & Preventive Medicine (ISPM), University of Bern, Switzerland ([Matthias.egger@ispm.unibe.ch](mailto:Matthias.egger@ispm.unibe.ch)) [↑](#footnote-ref-9)
