# Supplementary material for: Strengthening the Reporting of Observational Studies in Epidemiology for respondent-driven sampling studies: “STROBE-RDS” statement
Source: J Clin Epidemiol. 2015 Dec;68(12):1463–71. doi: 10.1016/j.jclinepi.2015.04.002 (PMC4669303; doi:10.1016/j.jclinepi.2015.04.002)
Supplement: Document 2 [file mmc2.doc]

**SUPPLEMENTARY MATERIAL #2**

**STRENGTHENING THE REPORTING OF OBSERVATIONAL STUDIES IN EPIDEMIOLOGY FOR RESPONDENT-DRIVEN SAMPLING STUDIES: *‘STROBE-RDS’* STATEMENT**

**CHECKLIST AND EXPLANATION AND ELABORATION**

**2015_06_13**

RichardG White[[1]](#footnote-2)*×, Avi J Hakim[[2]](#footnote-3)*, Matthew J Salganik[[3]](#footnote-4), Michael W SpillerError: Reference source not found, Lisa G Johnston[[4]](#footnote-5), Ligia Kerr[[5]](#footnote-6), Carl Kendall[[6]](#footnote-7), Amy DrakeError: Reference source not found, David Wilson[[7]](#footnote-8), Kate OrrothError: Reference source not found, Matthias Egger[[8]](#footnote-9), Wolfgang HladikError: Reference source not found

* Authors contributed equally to this work × Corresponding author

**CONTENTS**

- **AIM, SCOPE, DESCRIPTION AND DEVELOPMENT PROCESS**
- **SECTION 1: THE ‘STROBE RDS’ CHECKLIST**
- **SECTION 2: THE ‘STROBE RDS’ EXPLANATION AND ELABORATION**

**AIM**

In line with the aims of the STROBE initiative [1](#_ENREF_1) this ‘STROBE-RDS’ statement is intended to be a *minimum* checklist of *essential* items to report for studies using respondent-driven sampling (RDS).

**SCOPE**

The scope of this ‘STROBE-RDS’ statement has been limited to (a) epidemiological studies (the scope of the original STROBE guidelines), (b) cross-sectional studies (the most common RDS study design to date) and (c) RDS studies that seek to generate representative estimates for the target population (currently the most contentious and potentially most policy-relevant use of RDS). Further, as RDS is a sampling *and* a data analysis method [2](#_ENREF_2), guidelines for reporting on both aspects of RDS are provided. We also aim to provide a complete statement for use when reporting an RDS study without the need to refer to other documents (as requested during piloting of the checklist).

**DESCRIPTION OF CONTENTS OF THIS STATEMENT**

The first section of the statement contains the checklist table (Table S 1). The checklist table contains three substantive columns. The first column shows the original STROBE checklist for cross-sectional studies. The second column shows the extensions or modifications. The third column shows the complete STROBE-RDS checklist, combining the original STROBE checklist and the extensions and modifications. This structure allows us to follow the recommendation of the STROBE group to clearly highlight the differences from the original STROBE checklist (column 2), while also allowing us to provide a complete checklist (column 3) that can be used by authors without referring to multiple documents. Column 2 (the modification) was omitted from the table shown in the main text for reasons of space.

The second section of this statement contains the Explanation and Elaboration document (E&E). A rationale and explanation for each item in the checklist is presented. In line with the identified need to provide a complete statement that can be used to report on an RDS study without the need to refer to other documents, it is based on the STROBE checklist for cross-sectional studies[3](#_ENREF_3) and the E&E text from the original STROBE statement[4](#_ENREF_4).

**DEVELOPMENT PROCESS**

The STROBE-RDS statement was developed in accordance with the recommendations of *Moher et al.* [5](#_ENREF_5)After initial consultation, an initial draft checklist was published in the journal *Sexually Transmitted Infections* in October 2012.[6](#_ENREF_6) After further consultation, initial piloting, and incorporation of input from the STROBE group, this checklist was discussed and revised by a group of epidemiologists, statisticians and empirical RDS researchers at a meeting in New Orleans, Louisiana, in late October 2012 [7](#_ENREF_7). Following the meeting, an RDS E&E text was developed and the revised checklist and E&E were circulated to the wider RDS community, piloted and revised again. The final versions of the checklist and E&E are presented here. This statement will be updated periodically as RDS methods develop.

**SECTION 1:**

**THE ‘STROBE RDS’ CHECKLIST**

White, RG, Hakim, AJ, Salganik, MJ, Spiller, MW, Johnston, LG, Kerr, L, Kendall, C, Drake, A, Wilson, D, Orroth, K, Egger, M, Hladik, W

Table S 1 STROBE-RDS statement checklist. Italics highlight changes from STROBE statement checklist for cross-sectional studies [3](#_ENREF_3). ‘#’ = number.

| **Item** | **#** | **Original STROBE Checklist for Cross Sectional Studies** | **STROBE-RDS Extension / Modification** | **Complete STROBE-RDS Checklist *(italics highlight changes)*** |
| --- | --- | --- | --- | --- |
| **Title and Abstract** | 1 | (a) Indicate the study’s design with a commonly used term in the title or the abstract | Indicate "respondent-driven sampling" in the title or abstract | (a) Indicate *“respondent-driven sampling"* in the title or abstract |
|  |  | (b) Provide in the abstract an informative and balanced summary of what was done and what was found |  | (b) Provide in the abstract an informative and balanced summary of what was done and what was found |
| **Introduction** |  |  |  |  |
| *Background/ rationale* | 2 | Explain the scientific background and rationale for the investigation being reported |  | Explain the scientific background and rationale for the investigation being reported |
| *Objectives* | 3 | State specific objectives, including any pre-specified hypotheses |  | State specific objectives, including any pre-specified hypotheses |
| **Methods** |  |  |  |  |
| *Study design* | 4 | (a) Present key elements of study design early in the paper |  | (a) Present key elements of study design early in the paper |
|  |  |  | (b) State why RDS was chosen as the sampling method | *(b) State why RDS was chosen as the sampling method* |
| *Setting* | 5 | (a) Describe the setting, locations, and relevant dates, including periods of recruitment, exposure, follow-up, and data collection | Deletion of ‘exposure, follow-up,’ | *a) Describe the setting, locations, and relevant dates, including periods of recruitment, and data collection* |
|  |  |  | (b) Describe formative research findings used to inform RDS study | *(b) Describe formative research findings used to inform RDS study* |
| *Participants* | 6 | (a) Give the eligibility criteria, and the sources and methods of selection of participants | Add ‘Describe how participants were trained/ instructed to recruit others, number of coupons issued per person, and any time limits for referral’ | (a) Give the eligibility criteria, and the sources and methods of selection of participants. *Describe how participants were trained/ instructed to recruit others, number of coupons issued per person, any time limits for referral* |
|  |  |  | (b) Describe methods of seed selection and state number at start of study and number added later | *(b) Describe methods of seed selection and state number at start of study and number added later* |
|  |  |  | (c) State if there was any variation in study procedures during data collection (e.g., changing numbers of coupons per recruiter, interruptions in sampling, or stopping recruitment chains) | *(c) State if there was any variation in study procedures during data collection (e.g., changing numbers of coupons per recruiter, interruptions in sampling, or stopping recruitment chains)* |
|  |  |  | (d) Report wording of personal network size question(s) | *(d) Report wording of personal network size question(s)* |
|  |  |  | (e) Describe incentives for participation and recruitment | *(e) Describe incentives for participation and recruitment* |
| *Variables* | 7 | (a) Clearly define all outcomes, exposures, predictors, potential confounders, and effect modifiers. Give diagnostic criteria, if applicable | Add ‘If applicable,’ Replace ‘exposures‘ with ‘correlates’ | *(a) If applicable,* clearly define all outcomes, *correlates*, predictors, potential confounders, effect modifiers, and diagnostic criteria |
|  |  |  | (b) State how recruiter-recruit relationship was tracked | *(b) State how recruiter-recruit relationship was tracked* |
| *Data sources/ measurement* | 8 | (a) For each variable of interest, give sources of data and details of methods of assessment (measurement). Describe comparability of assessment methods if there is more than one group | Clarify use of ‘measurement’ | (a) For each variable of interest, give sources of data and details of methods of *measurement.* Describe comparability of *measurement* methods if there is more than one group |
|  |  |  | (b) Describe methods to assess eligibility and reduce repeat enrollment (e.g. coupon manager software, biometrics) | *(b) Describe methods to assess eligibility and reduce repeat enrollment (e.g. coupon manager software, biometrics)* |
| *Bias* | 9 | Describe any efforts to address potential sources of bias |  | Describe any efforts to address potential sources of bias |
| *Study size* | 10 | Explain how the study size was arrived at |  | Explain how the study size was arrived at |
| *Quantitative variables* | 11 | Explain how quantitative variables were handled in the analyses. If applicable, describe which groupings were chosen, and why |  | Explain how quantitative variables were handled in the analyses. If applicable, describe which groupings were chosen, and why |
| *Statistical methods* | 12 | (a) Describe all statistical methods, including those used to control for confounding | Add ‘those to account for sampling strategy (e.g. the estimator used) and, if applicable,’ | *(a) Describe all statistical methods, including those to account for sampling strategy (e.g. the estimator used) and, if applicable, those used to control for confounding* |
|  |  |  | (b) State data analysis software, version number and specific analysis settings used | *(b) State data analysis software, version number and specific analysis settings used* |
|  |  | (b) Describe any methods used to examine subgroups and interactions |  | (c) Describe any methods used to examine subgroups and interactions |
|  |  | (c) Explain how missing data were addressed |  | (d) Explain how missing data were addressed |
|  |  | (d) If applicable, describe analytical methods taking account of sampling strategy | Deleted (included in 12a) |  |
|  |  | (e) Describe any sensitivity analyses |  | (e) Describe any sensitivity analyses |
|  |  |  | (f) Report any criteria used to support statements on whether estimator conditions or assumptions were appropriate | *(f) Report any criteria used to support statements on whether estimator conditions or assumptions were appropriate* |
|  |  |  | (g) Explain how seeds were handled in analysis | *(g) Explain how seeds were handled in analysis* |
| **Results** |  |  |  |  |
| *Participants* | 13 | (a) Report the numbers of individuals at each stage of the study—e.g., numbers potentially eligible, examined for eligibility, confirmed eligible, included in the study, completing follow-up, and analyzed | Deleted ‘completing follow-up,’ | *a) Report the numbers of individuals at each stage of the study —e.g., numbers potentially eligible, examined for eligibility, confirmed eligible, included in the study, and analyzed* |
|  |  | (b) Give reasons for non-participation at each stage | Add ‘(e.g., not eligible, does not consent, decline to recruit others)’ | (b) Give reasons for non-participation at each stage *(e.g., not eligible, does not consent, decline to recruit others)* |
|  |  | (c) Consider use of a flow diagram |  | (c) Consider use of a flow diagram |
|  |  |  | (d) Report number of coupons issued and returned | *(d) Report number of coupons issued and returned* |
|  |  |  | (e) Report number of recruits by seed and number of RDS recruitment waves for each seed. Consider showing graph of entire recruitment network | *(e) Report number of recruits by seed and number of RDS recruitment waves for each seed. Consider showing graph of entire recruitment network* |
|  |  |  | (f) Report recruitment challenges (e.g. commercial exchange of coupons, imposters, duplicate recruits) and how addressed | *(f) Report recruitment challenges (e.g. commercial exchange of coupons, imposters, duplicate recruits) and how addressed* |
|  |  |  | (g) Consider reporting estimated design effect for outcomes of interest | *(g) Consider reporting estimated design effect for outcomes of interest* |
| *Descriptive data* | 14 | (a) Give characteristics of study participants (e.g., demographic, clinical, social) and information on exposures and potential confounders | Add ‘If applicable,’. Replace ‘exposures‘ with ‘correlates’. Add ‘Report unweighted sample size and percentages, estimated population proportions or means with estimated precision (e.g., 95% confidence interval)’ | a) Give characteristics of study participants (e.g., demographic, clinical, social) and, *if applicable,* information on *correlates* and potential confounders. *Report unweighted sample size and percentages, estimated population proportions or means with estimated precision (e.g., 95%* confidence interval*)* |
|  |  | (b) Indicate the number of participants with missing data for each variable of interest |  | (b) Indicate the number of participants with missing data for each variable of interest |
| *Outcome data* | 15 | Report numbers of outcome events or summary measures | Add ‘If applicable,’ | *If applicable,* report number of outcome events or summary measures |
| *Main results* | 16 | (a) Give unadjusted estimates and, if applicable, confounder-adjusted estimates and their precision (e.g., 95% confidence intervals). Make clear which confounders were adjusted for and why they were included | Add ‘and study design adjusted’ | (a) Give unadjusted *and study design adjusted* estimates and, if applicable, confounder adjusted estimates and their precision (e.g., 95% confidence intervals). Make clear which confounders were adjusted for and why they were included |
|  |  | (b) Report category boundaries when continuous variables were categorised |  | (b) Report category boundaries when continuous variables were categorised |
|  |  | (c) If relevant, consider translating estimates of relative risk into absolute risk for a meaningful time period | Original (c) deleted; replaced with text below |  |
|  |  |  | (c) If adjustment of primary outcome leads to marked changes, report information on factors influencing the adjustments (e.g. personal network sizes, recruitment patterns by group, key confounders) | *(c) If adjustment of primary outcome leads to marked changes, report information on factors influencing the adjustments (e.g. personal network sizes, recruitment patterns by group, key confounders)* |
| *Other analyses* | 17 | Report other analyses done—e.g., analyses of subgroups and interactions, and sensitivity analyses | Add ‘different RDS estimators and definitions of personal network size’ | Report other analyses done—e.g., analyses of subgroups and interactions, sensitivity analyses, *different RDS estimators and definitions of personal network size* |
| **Discussion** |  |  |  |  |
| *Key results* | 18 | Summarise key results with reference to study objectives |  | Summarise key results with reference to study objectives |
| *Limitations* | 19 | Discuss limitations of the study, taking into account sources of potential bias or imprecision. Discuss both direction and magnitude of any potential bias |  | Discuss limitations of the study, taking into account sources of potential bias or imprecision. Discuss both direction and magnitude of any potential bias |
| *Interpretation* | 20 | Give a cautious overall interpretation of results considering objectives, limitations, multiplicity of analyses, results from similar studies, and other relevant evidence |  | Give a cautious overall interpretation of results considering objectives, limitations, multiplicity of analyses, results from similar studies, and other relevant evidence |
| *Generalisability* | 21 | Discuss the generalisability (external validity) of the study results |  | Discuss the generalisability (external validity) of the study results |
| **Other information** |  |  |  |  |
| *Funding* | 22 | Give the source of funding and the role of the funders for the present study and, if applicable, for the original study on which the present article is based |  | Give the source of funding and the role of the funders for the present study and, if applicable, for the original study on which the present article is based |

**SECTION 2:**

**THE ‘STROBE RDS’ EXPLANATION AND ELABORATION**

White, RG, Hakim, AJ, Salganik, MJ, Spiller, MW, Johnston, LG, Kerr, L, Kendall, C, Drake, A, Wilson, D, Orroth, K, Egger, M, Hladik, W

Abbreviations

| AOR | Adjusted odds ratio |
| --- | --- |
| CI | Confidence interval |
| DE | Design effect |
| FSWs | Female sex workers |
| HBV | Hepatitis B virus |
| HCV | Hepatitis C virus |
| HIV | Human immunodeficiency virus |
| IBBS | Integrated Biologic and Behavioral Surveillance |
| IDU | Injecting drug user |
| LGBT | Lesbian, gay, bisexual, and transgender |
| MAR | Missing at random |
| MCAR | Missing completely at random |
| MNAR | Missing not at random |
| MSM | Men Who Have Sex with Men |
| NGO | Non-governmental organization |
| PEPFAR | President's Emergency Plan for AIDS Relief |
| PNG | Papua New Guinea |
| RDS | Respondent-driven sampling |
| RDSAT | RDS Analysis Tool |
| RDS-II | Respondent-driven sampling estimator number 2 |
| SES | Socio-economic status |
| SRS | Simple random sample |
| STI | Sexually transmitted infection |
| TLS | Time–location sampling |
| UAI | Unprotected anal intercourse |
| VCT | Voluntary counselling and testing |

**TITLE AND ABSTRACT (Item 1)**

**1(a) Indicate“respondent-driven sampling" in the title or abstract**

**Example**

“*HIV Infection among Men Who Have Sex with Men in Kampala, Uganda–A Respondent Driven Sampling Survey*”[8](#_ENREF_8)

**Explanation**

Readers should be able to easily identify the design that was used from the title or abstract. An explicit, commonly used term for the study design also helps ensure correct indexing of articles in electronic databases.

**1(b) Provide in the abstract an informative and balanced summary of what was done and what was found**

**Example**

*“Objectives: Undiagnosed HIV presents great potential for the spread of infection. The authors identify the prevalence and correlates of never testing and being unaware of HIV infection in Beijing men who have sex with men (MSM).*

*Methods: Cross-sectional biological and behavioural survey using respondent-driven sampling; 500 MSM were included.*

*Results: HIV prevalence was 7.2% with 86.1% unaware of their infection; 33.2% had never tested. Never testing was associated with lower educational (adjusted odds ratio (AOR) 1.6, 95% CI (CI) 1.1 to 2.5), living in Beijing for ≤3 years (AOR 1.5, 95% CI 1.0 to 2.3), unprotected anal intercourse with most recent male partner (AOR 1.6, 95% CI 1.0 to 2.4), being unaware of the most recent male partner’s HIV status (AOR 3.6, 95% CI 2.1 to 6.1) and holding stigmatised attitudes towards persons with HIV (AOR 1.1 per scale point, 95% CI 1.0 to 1.1). Predictors of having undiagnosed HIV infection were being married (AOR 2.4, 95% CI 1.0 to 5.4), living in Beijing for ≤3 years (AOR 3.6, 95% CI 1.5 to 8.4), being unaware of the most recent male partner’s HIV status (AOR 6.8, 95% CI 0.9 to 51.6) and holding negative attitudes towards safe sex (AOR 1.1 per scale point, 95% CI 1.0 to 1.1).*

*Conclusions: Recent attention has focused on HIV prevention interventions that depend upon knowing one’s serostatus, including viral load suppression, prevention with positives, pre-exposure prophylaxis and seroadaptation. Until the low level of testing and resulting high level of undiagnosed HIV infection are addressed, these tools are not likely to be effective for MSM in China.”* [11](#_ENREF_11)

**Explanation**

The abstract provides key information that enables readers to understand a study and decide whether to read the article. Typical components include a statement of the research question, a short description of methods and results, and a conclusion. Abstracts should summarize key details of studies and should only present information that is provided in the article. We advise indicating how data were analysed and presenting key results in a numerical form that includes number of participants and estimates of associations and appropriate measures of variability and uncertainty (e.g., odds ratios with confidence intervals). For risk factor studies, we regard it insufficient to state only that an exposure is or is not significantly associated with an outcome.

A series of headings pertaining to the background, methods, results, and conclusion of a study may help readers acquire the essential information rapidly.[12](#_ENREF_12) Many journals require such structured abstracts, which tend to be of higher quality and more readily informative than unstructured summaries.

**INTRODUCTION (Items 2 - 3)**

The Introduction section should describe why the study was done and what questions and hypotheses it addresses. It should allow others to understand the study’s context and judge its potential contribution to current knowledge.

**2 Background/rationale: Explain the scientific background and rationale for the investigation being reported**

**Example**

*“Men who have sex with men (MSM) have been disproportionately affected by the HIV pandemic due to a combination of structural, biological, social, and behavioral factors that increase their vulnerability to HIV infection. Even in countries with generalized HIV epidemics, the prevalence of HIV infection is considerably higher among MSM compared with men in the general population. Recent studies in several African countries confirm that HIV prevalence among MSM is high relative to the general population. In South Africa, little is known about the demographics, HIV prevalence, or risk factors for HIV among MSM in the local context, and current HIV policies and programs are largely unresponsive to the needs of MSM.”* [15](#_ENREF_15)

**Explanation**

The scientific background of the study provides important context for readers. It sets the stage for the study and describes its focus. It gives a balanced overview of what is known on a topic and what gaps in current knowledge are addressed by the study. Background material should provide a balanced account that references systematic reviews when possible over individual studies that seek to provide an unbalanced support of the narrative and that were chosen post hoc.

**3 Objectives: State specific objectives, including any pre-specified hypotheses**

**Example**

***“****We sought to study suicidal behavior prevalence and its association with social and gender disadvantage, sex work, and health factors among female sex workers in Goa, India.”*[16](#_ENREF_16)

**Explanation**

Objectives are the detailed aims of the study. Well-crafted objectives specify populations, exposures and outcomes, and parameters that will be estimated. They may be formulated as specific hypotheses or as questions that the study was designed to address. In some situations objectives may be less specific, for example, in early discovery phases. Regardless, the report should clearly reflect the investigators’ intentions. For example, if important subgroups or additional analyses were not the original aim of the study but arose during data analysis, they should be described accordingly (see also items 4, 17 and 20).

**METHODS (Items 4 - 12)**

The methods section should describe what was planned and what was done in sufficient detail to allow others to understand the essential aspects of the study, to judge whether the methods were adequate to provide reliable and valid answers, and to assess whether any deviations from the original plan were reasonable.

**4 Study Design**

**4(a) Present key elements of study design early in the paper**

**Example**

***“****This survey was conducted from April through June 2007 using strict adherence to RDS methodology* […]. *After the seeds were enrolled into the survey, they were provided three coupons to recruit the first wave of participants into the survey. Each wave of participants subsequently recruited the next wave of participants until the target sample size was reached. Individuals who presented a valid coupon at the interview location were screened for survey eligibility and asked to provide consent for a face-to-face interview and venous blood draw for HIV, syphilis, HBV and HCV testing. MSM peer educators administered the interview in Kiswahili which collected information on socio-demographic characteristics, sexual and drug use behaviors, STI symptoms, HIV knowledge, and participants’ social networks.*[…] *A coupon manager system was used to monitor recruitment, duplicity, and incentive distribution.”*[17](#_ENREF_17)

**Explanation**

We advise presenting key elements of study design early in the methods section (or at the end of the introduction) so that readers can understand the basics of the study. The population and the point in time at which the sample was taken should be mentioned. We recommend that authors describe exactly how and when data collection took place (see also items 5a and 6a). Explicit reporting on RDS elements is essential for determining if study methods constitute RDS or another modified snowball sampling method. Authors should describe number of coupons provided, whether personal network size information was collected, and if recruiter-recruit relationships were tracked and how (see also 6c and 7b).

The first part of the methods section might also be the place to mention whether the report is one of several from a study. If a new report is in line with the original aims of the study, this is usually indicated by referring to an earlier publication and by briefly restating the salient features of the study. However, the aims of a study may also evolve over time. Researchers often use data for purposes for which they were not originally intended, including, for example, official vital statistics that were collected primarily for administrative purposes, items in questionnaires that originally were only included for completeness, or blood samples that were collected for another purpose. The secondary use of existing data is a creative part of observational research and does not necessarily make results less credible or less important. However, briefly restating the original aims might help readers understand the context of the research and possible limitations in the data.

**4(b)State why RDS was chosen as the sampling method**

**Example**

*“Our formative research showed that there was a relatively large and socially well connected population of MSM in Port Moresby, the capital and largest city of PNG. Although there were a number of commercial and public spaces where MSM met and socialised, none of them catered to a primarily MSM clientele. There were no formal community–based or social organizations of gay or homosexual men. Due to social stigma and discrimination, the vast majority of MSM kept their sexual orientation hidden from their family, friends and co–workers. The lack of MSM–identified sites and the hidden nature of homosexuality in PNG meant that it would be difficult to use time–location sampling (TLS) as the recruitment methodology.”*[18](#_ENREF_18)

**Explanation**

We advise describing why RDS was chosen as the sampling method and why other methods were not, including the trade-offs between methods. Formative assessment is essential for all RDS studies because the RDS method requires active participation and recruitment by the target population and makes assumptions that should be assessed to be plausible before implementation. We advise stating formative research findings that may impact success or failure of recruitment (see also item 5b). For example, assess the existence of a sufficiently large population with a connected social network; if members of the study population can report the size of their social network; and whether they can identify each other, are willing to participate, and can recruit others to participate.

**5 Setting**

**5(a) Describe the setting, locations, and relevant dates, including periods of recruitment, and data collection**

**Example**

***“****Between February and April 2005, RDS was used to recruit IDUs in Tijuana […] and Ciudad Juarez […] Interviews were conducted in Spanish at the NGO-sponsored clinic of Programa Companeros in Ciudad Juarez and in the mobile clinic of the NGO COMUSIDA in Tijuana.”*[19](#_ENREF_19)

**Explanation**

Readers need the information necessary to assess the context and generalisability of a study’s results. Information about location may refer to the countries or towns where the investigation took place as well as a description of the study site (e.g., rented storefront or apartment, shared space with a community organization or clinic, etc). We advise indicating if multiple study sites were used and how they operated in relation to one another (e.g., same staff, different days, if recruitment between sites was permitted, how data were managed, etc).

Study methods may evolve over time. Knowing when a study took place and over what period participants were recruited and followed up places the study in historical context and is important for the interpretation of results. It further facilitates an understanding of the speed at which participants were recruited. We advise reporting starting and stopping dates rather than only describing the length of time periods. There may be different sets of dates for exposure, recruitment, beginning and end of follow-up, and data collection. Also report which days of the week and hours study sites were open as these will impact participation.

**5(b)Describe formative research findings used to inform RDS study**

**Example**

*“Interviewed FSWs reported degree sizes ranging from 1 to 20 and indicated that FSWs form strong and weak ties through their relationships as friends, acquaintances, and co-workers. FSWs comprised cliques in four major sex work areas and indicated that few FSWs formed bridges across these cliques. Lower-paid FSWs, most of whom had previous interaction with outreach staff, were more interested in participating in the survey than were higher-paid FSWs. Interviews indicated that incentives would be difficult to set since having one incentive level might be too low for higher-paid FSWs and too high for lower-paid FSWs. Interviewees suggested few available interview sites, that public health clinics were not acceptable, and that given the traffic in Bangkok multiple survey sites close to working areas would be needed […] Formative research indicated that higher-paid FSWs would be less interested in participating in the survey than lower-paid FSWs, due to the level of incentive and to their lack of exposure to outreach work or previous surveys.”*­­­­[20](#_ENREF_20)

**Explanation**

A brief description of the formative research conducted prior to the RDS study, particularly an understanding of population characteristics, can yield insight into the strengths and limitations of study data (see also item 19). Important formative assessment findings to report include: demographic and other characteristics of the study population and sub-groups, identification of socially salient variables, potential network bottlenecks (lack of social relationships) between sub-groups, opinion of target population members regarding willingness to participate and recruit others, willingness of participants to provide biological specimens, and study logistics (e.g., incentives, study site location, and hours of operation) (see also item 4b). Report if formative research, such as monitoring recruitment and community perception, was continued during the course of the RDS study, and how formative findings were used to make changes in the design or implementation of the study. If a formative assessment was not conducted, explain what background knowledge already existed to justify not conducting formative research.

**6 Participants**

**6(a) Give the eligibility criteria, and the sources and methods of selection of participants. Describe how participants were trained/instructed to recruit others, number of coupons issued per person, and any time limits for referral**

**Example**

*“Eligible participants were children aged between 10 and 17 years who worked on the streets in Tirana. For this study, working on the streets included begging, stealing, selling items, retrieving items from the street or garbage dumps, washing cars and other street based activities that involve earning money, food or other subsistence items [...] Participants were provided with three coupons, educated on how to use those coupons to recruit their peers and how to receive a secondary incentive for each successful recruit [...] [Participants] were instructed to recruit children with diverse characteristics (to ensure inclusion of different subgroups) and not to recruit strangers.”* [21](#_ENREF_21)

**Explanation**

Detailed descriptions of the study participants help readers understand the applicability of the results. Investigators usually restrict a study population by defining clinical, demographic and other characteristics of eligible participants. Typical eligibility criteria relate to age, sex, risk behaviour, language, and residency. Despite their importance, all eligibility criteria elements are often not clearly reported.

Eligibility criteria may be presented as inclusion and exclusion criteria, although this distinction is not always necessary or useful. Regardless, we advise authors to report all eligibility criteria and also to describe the group from which the study population was selected (e.g., the general population of a region or country).

Additional eligibility issues specific to RDS include efforts to identify repeat participants or imposters. We advise reporting efforts undertaken to screen candidate participants and measures taken to reduce such situations.

Recruitment instructions given to participants should be described to reveal potential biases. How participants were instructed to recruit others may inadvertently reveal full recruitment criteria to potential participants and bias their recruitment efforts [22](#_ENREF_22). Participants may be instructed to recruit the first eligible peers they meet or a specific kind of person that seems to be underrepresented in the study. The randomness of selection may differ based on the guidance participants were given. Expiration times (the amount of time allotted to recruit peers) and validation times (the waiting time before participants can distribute their coupons) may influence the number and kinds of people eligible for recruitment.

**6(b)Describe methods of seed selection and state number at start of study and number added later**

**Example**

*“Concurrent with the formative phase, eight demographically diverse initial participants (seeds) were nonrandomly selected with input from key informants to serve as the starting points for recruitment. Criteria for seed selection included leadership in the transwomen community, place of living or social interaction, age and type of work. […] A total of 450 transwomen were recruited between April and July 2009. Of these, 11 were seeds (8 initial seeds plus 3 additional seeds)”* [23](#_ENREF_23)

**Explanation**

Seeds may have the same eligibility criteria as other participants or not. Indicate criteria and efforts undertaken to purposively diversify seeds. Investigators may add additional seeds during the course of the study to speed recruitment or recruit from seemingly underrepresented subgroups. The reasons for the addition of seeds should be described, and it should be noted if new seeds were selected through a different procedure than initial seeds.

**6(c)State if there was any variation in study procedures during data collection (e.g., changing numbers of coupons per recruiter, interruptions in sampling, or stopping recruitment chains)**

**Example 1**

*“Sampling was interrupted twice; first as a result of the arrest of three local lesbian, gay, bisexual, and transgender (LGBT) activists at the PEPFAR Implementer’s Meeting in Kampala in early June 2008. Sampling remained at a low level until it rebounded in August, but was again affected in September 2008 by arrests of alleged homosexuals. A total of six additional seeds were added during the survey. However, following the second round of arrests, sampling rates remained low until we stopped sampling in March 2009, close to the survey’s scheduled end.”*[8](#_ENREF_8)

**Example 2**

*“The rate of early recruitment was high, and the number of people arriving each day for interviews became too large to be manageable. Because of this, between days 9 and 32, the probability of each recruit being offered 3 coupons was halved from 100% to 50%; other recruits received none.”* [24](#_ENREF_24)

**Explanation**

Modifications to study operating procedures during the course of an RDS study are not uncommon and may be appropriate so long as they are well documented and respect the functional and analytic assumptions of RDS. Describe why, when and how changes were made.

Describe any substantial events that materially affected data collection and operations, particularly if the event resulted in a response or corrective action by investigators. State whether sampling interruptions, such as those due to holidays or political violence, were anticipated or not. Some interruptions may result in the temporary or permanent closure of study sites and others simply a decrease in the speed of recruitment. All interruptions should be explained; be sure to indicate when in the course of the study implementation they occurred. Indicate if interruptions cause the permanent end of recruitment chains or a temporary slow-down. Whether or not recruitment recovers from interruptions should be noted. Researchers should also report whether study procedures had to be modified in response to coupon-selling or imposters.

**6(d)Report wording of personal network size question(s)**

**Example 1**

*“Participants’ personal social network sizes were measured by a cascade of questions that arrived at a number of MSM who were 18 or older, with whom they were familiar, met or talked to in the two previous months, and who they might invite to participate in this study.”* [25](#_ENREF_25)

**Example 2**

*“Recruits were first asked the core question ‘How many men do you know who (i) were head of a household in the last 12 months in any of the Medical Research Council villages, and (ii) you know them and they know you, and (iii) you have seen them in the past week.’ ”* [24](#_ENREF_24)

**Explanation**

Personal network size is currently used as a proxy measure of the number of people who might recruit that individual. At present, personal network size questions are essential for determining sample weights for most RDS estimators. The questions asked, their timing, and how they are asked can yield different results. Understanding what questions were asked can facilitate comparison across studies and contribute to standardized personal network size questions. If only one personal network size question was asked, present the exact wording of the question. If a series or cascade of questions was asked, summarize the series and indicate the exact wording of the question used to obtain sampling weights.

**6(e) Describe incentives for participation and recruitment**

**Example:**

*“As incentives for participation and recruitment, seeds and recruits were offered soap, salt, or school books to the value of approximately US $1. One incentive was offered for completing the first interview and another for each person successfully recruited.”* [24](#_ENREF_24)

**Explanation:**

Incentives are normally given in RDS studies for initial participation (participation incentive) and recruitment (recruitment incentive) of others. We advise describing whether incentives were given, for what they were given, their value and form, and whether they changed during the course the study. Such information is useful when considering potential biases and may help explain the success or failure of recruitment. Report the value in a common currency (e.g., US dollar or Euro).

**7 Variables**

**7(a) *If applicable,* clearly define all outcomes, correlates, predictors, potential confounders, effect modifiers and diagnostic criteria**

**Example**

***“****We constructed regression models to determine the association between HIV risk factors and two outcomes: HIV status and UAI [unprotected anal intercourse] with male partners in the last 12 months. […] We hypothesized a priori that age, [personal] social network size and education were the only confounders in the relationship between the risk factors and the outcomes.”*[26](#_ENREF_26)

**Explanation**

Authors should define all variables considered for and included in the analysis, including outcomes, correlates, predictors, potential confounders and potential effect modifiers. Disease outcomes require adequately detailed description of the diagnostic criteria.

For some studies, ‘determinant’ or ‘predictor’ may be appropriate terms for exposure variables and outcomes may be called ‘endpoints’. In multivariable models, authors sometimes use ‘dependent variable’ for an outcome and ‘independent variable’ or ‘explanatory variable’ for exposure and confounding variables. The latter is not precise as it does not distinguish exposures from confounders.

Finally, we advise that authors declare all ‘candidate variables’ considered for statistical analysis, rather than selectively reporting only those included in the final models (see also item 16a).

**7(b) State how recruiter-recruit relationship was tracked**

**Example 1**

*“To track the coupons and payment for each respondent, we used custom-developed software for RDS called IRIS Plus. Information such as respondent’s unique code, physical traits, coupon number, and the numbers of the coupons each respondent distributed were all recorded in IRIS Plus. This information enabled us to link coupons together, determine […] who gave coupons to whom.”* [29](#_ENREF_29)

**Explanation**

The recruiter-recruit relationship is an integral component of RDS implementation and analysis so it must be carefully tracked. This relationship can provide information about recruitment biases when recruitment patterns are examined (see also item 16c). Furthermore, it may be required for certain types of statistical analyses that attempt to better adjust for the RDS study design (see items 14a and 16a). The recruiter-recruit relationship can be tracked through coupon numbers, study IDs or other mechanisms. We advise indicating if a coupon manager program was used to facilitate tracking.

**8 Data Sources/Measurement**

**8(a) For each variable of interest, give sources of data and details of methods of measurement. Describe comparability of measurement methods if there is more than one group**

**Example**

*“The Alcohol Use Disorders Identification Test (AUDIT) was used to characterize drinking behavior. AUDIT assessments include frequency of drinking, amount of alcohol consumed when drinking, and perceptions of self and others about drinking; a score greater than 9 indicates ‘‘problem drinking.’’ ”* [30](#_ENREF_30)

**Explanation**

The way in which risk factors, confounders and outcomes were measured affects the reliability and validity of a study. Measurement error and misclassification of risk factors or outcomes may produce spurious relationships. Error in measurement of potential confounders can increase the risk of residual confounding. Authors should report the validity or reliability of assessments or measurements in study findings, including details of the reference standard that was used. Rather than simply citing validation studies, we advise that authors give the estimated validity or reliability, which can then be used for measurement error adjustment or sensitivity analyses (see items 12e and 17).

In addition, report groups being compared differed with respect to the way in which the data were collected. This may be important for laboratory examinations, in the case of multiple study sites, serial studies, and other situations.

**8(b) Describe methods to assess eligibility and reduce repeat enrollment (e.g. coupon manager software, biometrics)**

**Example 1**

*“Repeat participation was avoided by through [sic] eligibility assessment which asked if the potential participant had recently completed a survey for the implementing NGO and supported by staff recognition of duplicate participants.”* [26](#_ENREF_26)

**Example 2**

*“[The] software also helped to prevent the redemption of duplicated coupons because the database would not accept duplicate coupon numbers. When respondents came in to make an appointment, the coupon was checked in the IRIS Plus database to verify that it had not been used previously. Additionally, each person’s code was checked in IRIS Plus after being screened to determine whether a person with that code had previously been enrolled in the study. If another study subject had that code, the screener looked at the physical traits listed for that person for verification. Additionally, if the screener felt that a person looked familiar, she would search for the person’s physical traits in IRIS Plus to see if another person with similar characteristics was previously enrolled.”* [29](#_ENREF_29)

**Example 3**

*“Eligibility criteria for the study included: having injected illicit drugs within the past month, confirmed by inspection of injection stigmata (‘track marks’).”* [33](#_ENREF_33)

**Explanation**

Because RDS studies rely on participants to recruit their peers they may be more vulnerable than other study designs to imposters who misrepresent themselves in order to get into the study and earn incentives. Imposters and repeat participants skew results due to fictitious or duplicate data, and distort recruitment chains and recruiter-recruit relationships. Screening for all eligibility requirements including repeat enrollment, as well as for commercial exchange of coupons and coupons given to strangers can decrease bias and improve data validity. We advise reporting the frequency of such instances, what monitoring tools were utilized to prevent them, and describe how they were addressed (e.g., new chains created, existing chains stopped, individuals deemed ineligible, individuals allowed to participate, etc.).

**9 Bias: Describe any efforts to address potential sources of bias**

**Example**

***“****The questionnaires were administered in each of the five districts in order to minimise the travel time required for participation and eliminate any potential bias for non-participation from the more distant rural areas.”* [34](#_ENREF_34)

**Explanation**

Biased studies produce results that differ systematically from the truth. Describe what measures were taken during the conduct of a study to reduce the potential for bias (see also items 5b, 6b, 6d, 6e, 8b, 12a). Ideally, investigators carefully consider potential sources of bias when they plan their study. At the reporting stage we recommend that authors always assess the likelihood of relevant biases. Specifically, the direction and magnitude of bias should be discussed and, if possible, estimated. Unfortunately, authors often do not address important biases when reporting their results.

**10 Study Size: Explain how the study size was arrived at**

**Explanation**

A study should be large enough to obtain a point estimate with a sufficiently narrow confidence interval to meaningfully answer a research question. Large samples are needed to distinguish a small association from no association and to reliably detect changes over time. Small studies often provide valuable information, but wide confidence intervals may indicate that they contribute less to current knowledge in comparison with studies providing estimates with narrower confidence intervals. Also, small studies that show ‘interesting’ or ‘statistically significant’ associations are published more frequently than small studies that do not have ‘significant’ findings. While these studies may provide an early signal in the context of discovery, readers should be informed of their potential weaknesses.

The importance of sample size determination in observational studies depends on the context. If an analysis is performed on data that were already available for other purposes, the main question is whether the analysis of the data will produce results with sufficient statistical precision to contribute substantially to the literature, and sample size considerations will be informal. Formal, a priori calculation of sample size may be useful when planning a new study. Such calculations are associated with more uncertainty than implied by the single number that is generally produced. For example, estimates of the prevalence of the event of interest or other assumptions central to calculations are commonly imprecise, if not guesswork. [37](#_ENREF_37) The precision obtained in the final analysis often cannot be determined beforehand because it will be reduced by inclusion of confounding variables in multivariable analyses, [38](#_ENREF_38) the degree of precision with which key variables can be measured, [39](#_ENREF_39) and the exclusion of some individuals.

Few epidemiological studies explain or report the methods used to calculate sample size. We encourage investigators to report pertinent formal sample size calculations if they were done and the factors that went into the calculation, including the outcome variable of interest around which the calculation was built and the assumed design effect. In other situations authors should indicate the considerations that determined the study size (e.g., a fixed available sample, resource limitations, or experience with previous studies). It should be stated whether the study was stopped early when statistical significance was achieved. It should be realized that confidence intervals reflect statistical uncertainty only, and not other uncertainty that may be present in a study (see item 20).

**11 Quantitative Variables: Explain how quantitative variables were handled in the analyses. If applicable, describe which groupings were chosen, and why**

**Example**

*“Several demographic variables used to describe the study population are shown in Table 2. Levels of socio-economic status (SES) were categorized as: ‘Low,’ reported by participants as, ‘‘We don’t have enough money for food,’’ or ‘‘We have enough money for food but not for other basic items such as clothes;’’ ‘Medium,’ reported by participants as, ‘‘We have enough money for food and clothing but are short of many other things,’’ or ‘‘We have the most important things but few luxury goods;’’ and ‘high,’ reported by participants as, ‘‘We have money for luxury goods and extra things.’’”* [42](#_ENREF_42)

**Explanation**

Investigators make choices regarding how to collect and analyse quantitative data about exposures, effect modifiers and confounders. For example, they may group a continuous exposure variable to create a new categorical variable. Grouping choices may have important consequences for later analyses. We advise that authors explain why and how they grouped quantitative data, including the number of categories, the cut-points, and the sample mean or median values. Whenever data are reported in tabular form, the number of participants should be given for each category. Tables should not consist solely of effect-measure estimates or results of model fitting.

Investigators might model an exposure as continuous in order to retain all the information. In making this choice, one needs to consider the nature of the relationship of the exposure to the outcome. As it may be wrong to assume a linear relation automatically, possible departures from linearity should be investigated. Authors could mention alternative models they explored during analyses (e.g., using log transformation, quadratic terms or spline functions). Several methods exist for fitting a nonlinear relation between the exposure and outcome. Also, it may be informative to present both continuous and grouped analyses for a quantitative exposure of prime interest.

**12 Statistical Methods**

**12(a) Describe all statistical methods, including those to account for sampling strategy (e.g. the estimator used) and if applicable those used to control for confounding**

**Example 1**

*“In the RDS arm, estimates were obtained using both the RDS Analysis Tool (RDSAT) V6.0.1 and an RDS-II estimator. Only the RDS-II estimates are presented here. (See online supplementary material for a description of the RDS-II methods include the bootstrap estimator for the CIs and for comparison of RDS-II and RDSAT estimates.)”* [43](#_ENREF_43)

**Example 2**

*“The Table shows the comparison between the* […] *sample proportions, and RDS-1 and RDS-2 estimates . .”* [24](#_ENREF_24)

**Explanation**

Features that make RDS an effective data collection method in hard to reach populations also complicate the analysis of RDS data. Authors should describe methods they used to adjust for the sample design in their analysis, both when making estimates and when quantifying the uncertainty in those estimates.

Several different estimators exist for estimating the prevalence of a specific trait (e.g., HIV prevalence) from RDS data [44-50](#_ENREF_44). There are also a number of different methods for producing confidence intervals around these estimates . Evaluations of these methods have been equivocal and the best estimator may depend on specific features of a study [55](#_ENREF_55). At this time there is no consensus that one estimator should be universally used. As such, we recommend authors clearly describe the statistical methods used, including those to adjust for sample design, both when making estimates and when quantifying the uncertainty in those estimates. If multiple estimators were used to make estimates, each estimate should be presented.

Many approaches have also been used to perform multivariate analysis using RDS data . Authors should clearly state which approach was used and why. If multiple approaches were used, results from each approach should be presented.

Other decisions that should be reported include any ‘trimming’ of ‘outliers’, ‘data smoothing’, or imputation used, either based on an explicit choice by the researcher or an implicit choice embedded in the software used.

Investigators should pre-determine analyses at least for the primary study objectives in a study protocol. Often additional analyses are needed, either instead of, or as well as, those originally envisaged, and these may sometimes be motivated by the data. Authors should report whether particular analyses were motivated by data inspection. Even though the distinction between pre-specified and exploratory analyses may sometimes be blurred, authors should clarify reasons for particular analyses.

In general, there is no one correct statistical analysis but, rather, several possibilities that may address the same question, but make different assumptions. If groups being compared are not similar with regard to some characteristics, adjustment should be made for possible confounding variables by stratification or by multivariable regression.[63](#_ENREF_63) Analysts should fully describe specific procedures for variable selection and not only present results from the final model. If model comparisons are made to narrow down a list of potential confounders for inclusion in a final model, this process should be described. It is helpful to tell readers if one or two covariates are responsible for a great deal of the apparent confounding in a data analysis. Other statistical analyses such as imputation procedures, data transformation, and calculations of attributable risks should also be described. Nonstandard or novel approaches should be referenced. As a guiding principle, we advise statistical methods be described “with enough detail to enable a knowledgeable reader with access to the original data to verify the reported results.” [66](#_ENREF_66)

**12(b) State data analysis software, version number and specific analysis settings used**

**Example 1**

*“Estimates and 95% confidence intervals (CI) (Table 1) were calculated with RDS Analyst version 9.0 (www.hpmrg.org) using the successive sampling estimator* [*67*](#_ENREF_67) *with an assumed population size of 1500 MSM.”* [68](#_ENREF_68)

**Example 2**

*“The options used in the RDSAT software were:*

- *Personal network size estimation: dual component (default)*
- *Mean Cell Size: 12 (default)*
- *Number of re-samples required for bootstrap: 2,500 (default)*
- *Confidence interval (2-tailed alpha): 0.025 per tail*
- *Did not pull in outliers of personal network sizes (default)*
- *Algorithm type: Data smoothing (default)”* [43](#_ENREF_43)

**Explanation**

If existing software is used, provide the version number and specific analysis settings used in order to facilitate interpretation of results and replication of analysis methods. Similarly, if custom-written software is used, the code should be made available to other researchers.

**12(c) Describe any methods used to examine subgroups and interactions**

**Example**

*“To control for potential confounding, we first used stratified analyses to evaluate associations between participants’ HCV status and that of their last injection equipment sharing partner after stratification by the length of participants’ injection history, area of residence and injection frequency. Mantel–Haenszel calculations were used to derive an estimate for the p-value of a common odds ratio across strata and a Breslow–Day test was used to evaluate homogeneity of the odds ratio across strata.”*  [69](#_ENREF_69)

**Explanation**

As discussed in detail under item 17, many debate the use and value of analyses restricted to subgroups of the study population. Subgroup analyses are nevertheless often done. [40](#_ENREF_40) Readers need to know which subgroup analyses were planned in advance, and which arose while analyzing the data. Also, it is important to explain what methods were used to examine whether effects or associations differed across groups (see item 17).

**12(d) Explain how missing data were addressed**

**Example 1**

*“All variables are dichotomized and analyzed independently.[…] In the dichotomization, all non-group respondents, including those labeled as “missing,” are coded as part of the non-group. Including missing values as members of the non-group increases the number of recruitments in the 2008 sample by six for race and one for college. There are no missing data in the 2004 sample.”*[54](#_ENREF_54)

**Explanation**

Missing data are common in observational research. Questionnaires posted to study participants are not always filled in completely, and participants may not attend all follow-up visits. Despite its ubiquity and importance, few papers report in detail on the problem of missing data. Investigators may use any of several approaches to address missing data. We advise that authors report the number of missing values for each variable of interest (exposures, outcomes, confounders) and for each step in the analysis. Authors should give reasons for missing values if possible, and indicate how many individuals were excluded because of missing data when describing the flow of participants through the study (see also item 13). For analyses that account for missing data, authors should describe the nature of the analysis (e.g., multiple imputation) and the assumptions that were made (e.g., missing at random).

In RDS analysis, missing data on personal network size should receive particular attention given that this information is critical for most current estimators. If the software being used for analysis automatically makes certain assumptions to handle missing data, these assumptions should be stated and justified.

**12(e) Describe any sensitivity analyses**

**Example 1**

*“To determine the sensitivity of point estimates of the prevalence of syphilis antibody to modeling assumptions, we also obtained estimates using unadjusted rather than adjusted network sizes and using a ‘smoothed’ transition matrix based on the best fitting log-linear model, rather than the raw counts.”*[33](#_ENREF_33)

**Example 2**

*“Estimates were calculated for nontruncated, 1%, 5%, and 10% truncation for [measures of personal network size] in 2004 and 2008. Pulling in degree outliers had no effect, positive or negative, on estimates in 2004 or 2008.”* [54](#_ENREF_54)

**Explanation**

Sensitivity analyses are useful to investigate whether or not the main results are consistent with those obtained with alternative analysis strategies or assumptions. Issues that may be examined include the criteria for inclusion in analyses, the definitions of exposures or outcomes, [72](#_ENREF_72) which confounding variables merit adjustment, the handling of missing data, [73-75](#_ENREF_73) possible selection bias or bias from inaccurate or inconsistent measurement of exposure, disease and other variables, and specific analysis choices, such as the treatment of quantitative variables (see item 11). Sophisticated methods are increasingly used to simultaneously model the influence of several biases or assumptions.

In addition to the standard sensitivity analyses described above, some analyses particularly suited to RDS data are: 1) comparing estimates from several different RDS estimators that make different assumptions ; 2) comparing estimates using different measures of personal network size ; and 3) comparing estimates from different seeds .

**12(f)Report any criteria used to support statements on whether estimator conditions or assumptions were appropriate**

**Example 1**

***“****We used RDSAT to create a matrix of cross-recruitments. To determine whether the IDU networks within each city were linked, cross-recruitment was assessed for field site, as networks often are defined by geography. An example of cross-recruitment is when a participant interviewed at Field Site B had received his/her coupon from a recruiter interviewed at Field Site A. We also assessed cross-recruitment for the 5 variables; we report data only for race/ethnicity as it had the most impact on sampling. To be considered linked at least one recruitment between any two field sites or any two racial/ethnic groups, respectively, was required. The presence of at least one cross-recruitment in the sample suggests the presence of a large number of connections across groups in the population; the higher the proportion of cross-recruitments, the greater the number of network connections among IDUs.”*[80](#_ENREF_80)

**Example 2**

*“Comparing actual recruitment proportions with expected recruitment proportions calculated from individual-level network data, there was evidence of nonrandom recruitment by age, tribe, socioeconomic status, village, and sexual activity.”* [24](#_ENREF_24)

**Explanation**

Because investigators do not fully control the recruitment process with RDS, a number of assumptions are needed about the target population and the recruitment process in order to make estimates from RDS data. If the assumptions of a specific estimator are not met, then the estimates can suffer . Three main approaches have appeared in the literature for dealing with the assumptions underlying RDS inference: 1) simulation studies studying the robustness of estimators to violations of assumptions ; 2) development of new estimators that rely on different assumptions [47-49](#_ENREF_47); 3) development of diagnostics to check whether specific assumptions have been met . Investigators should indicate which diagnostic approaches were used.

Rather than thinking of assumptions as being met or not, we recommend that investigators think of the assumptions as necessary approximations and focus on how reasonable the assumptions are for their study. Before data collection begins investigators should consider whether specific assumptions are in doubt in their context and collect the data needed to assess the assumption (see also items 4b and 5b).

**12 (g) Explain how seeds were handled in analysis**

**Explanation**

Because seeds are selected through a different sampling process than peer-recruited participants, they are usually handled differently in data analysis . It is therefore important to specify how the seeds were handled in the analysis.

**RESULTS (Items 13 - 17)**

**13 Participants**

**13(a) Report the number of individuals at each stage of the study e.g., numbers potentially eligible, examined for eligibility, confirmed eligible, included in the study and analysed.**

**Example**

***“****The initial seven seeds ultimately reached 537 individuals through their social networks. Of these, a reported 118 declined to accept the recruitment coupons and 88 presenting to the study site were ineligible. Only three eligible respondents refused to participate in the study. The final sample included 321 FSW who completed both behavioral and serological steps of the study.”*[84](#_ENREF_84)

**Explanation**

Detailed information on the process of recruiting participants is important for several reasons. Those included in a study often differ in relevant ways from the target population to which results are then generalised. This may result in estimates of prevalence or incidence that do not reflect the experience of the target population. Such response bias can distort exposure-disease associations if associations differ between those eligible for the study and those included in the study. Ideally, investigators should give an account of the numbers of individuals considered at each stage of recruiting study participants, from the choice of a target population to the inclusion of participants’ data in the analysis. Depending on the type of study, this may include the number of individuals considered to be potentially eligible, the number assessed for eligibility (candidate participants), the number found to be eligible, the number consenting, the number interviewed (participants), the number given coupons for recruitment, and the number included in the analysis.

The follow-up interview is useful for learning about the recruitment process. Describe the number of participants returning for a second visit and findings from any follow-up interview to learn about recruitment (see also items 4a and 13f).

**13(b) Give reasons for non-participation at each stage (e.g., not eligible, does not consent, decline to recruit others)**

**Example**

*“A total of 1141 people (including the 10 seeds) were assessed for eligibility during a period of 54 days (8 March–30 April 2010). […] One hundred ninety-six men attended but were ineligible; 16 were eligible but had already been recruited; 2 were eligible but did not give consent; and 927 were eligible, consented, and were recruited. […] Overall, 75% of recruits (including seeds) (n = 684) were offered coupons to recruit others, and of these, 90% (n = 612) accepted (called “recruiters”). Sixty-six percent of recruiters (n = 401) returned to take part in a second interview and to collect their secondary incentives.“* [24](#_ENREF_24)

**Explanation**

Explaining the reasons why people did not participate or why they were excluded from statistical analyses helps readers judge how representative the study population is of the target population. For example, in a cross-sectional health study, non-participation due to reasons unlikely to be related to health status will affect the precision of estimates but will probably not introduce bias. Conversely, if many individuals opt out of the study because, for example, they fear they are HIV positive, or alternatively because of low perceived risk, results may underestimate or overestimate the prevalence of HIV in the population. Report numbers of people returning for second visit interview (see also item 13a).

Although low participation does not necessarily compromise the validity of a study, transparent information on participation and reasons for nonparticipation is essential. Also, as there are no universally agreed definitions for participation, response or follow-up rates, readers need to understand how authors calculated such proportions. [85](#_ENREF_85)

**13(c) Consider use of a flow diagram**

**Explanation**

An informative and well-structured flow diagram can readily and transparently convey information on number of individuals at each stage of the study that might otherwise require a lengthy description. [86](#_ENREF_86) Because of the complex recruitment process in RDS studies, we recommend that a flow diagram be used, focussing on the recruitment process. It should clearly state the number of individuals number screened, eligible and consented as seeds, the number screened, eligible and consented as peer-recruited participants, the number of peer-recruited participants offered coupons for recruitment, and the number of peer-recruited participant attending follow up interviews. An example of a well-structured flow diagram was not found in the RDS literature.

**13(d) Report number of coupons issued and returned**

**Example***“In total we distributed 1,053 coupons, with 614 (58.3%) returned. Of the returned coupons, 88 (14.3%) were screened as non-eligible, 3 (0.5%) refused to participate and 523 (85.2%) participated in and completed the survey; 439 (41.7%) never redeemed their coupons.”* [87](#_ENREF_87)

**Explanation**

More coupons will be distributed than returned. If multiple study sites were used, we advise reporting the extent of cross-recruitment between sites.

**13(e) Report number of recruits by seed and number of RDS recruitment waves for each seed. Consider showing graph of entire recruitment network.**

**Example 1**

**Figure:** Summary of the dynamics of respondent-driven sampling survey recruitment. […] (B), The total number of recruits per seed (excluding seeds). [24](#_ENREF_24)

**Example 2**


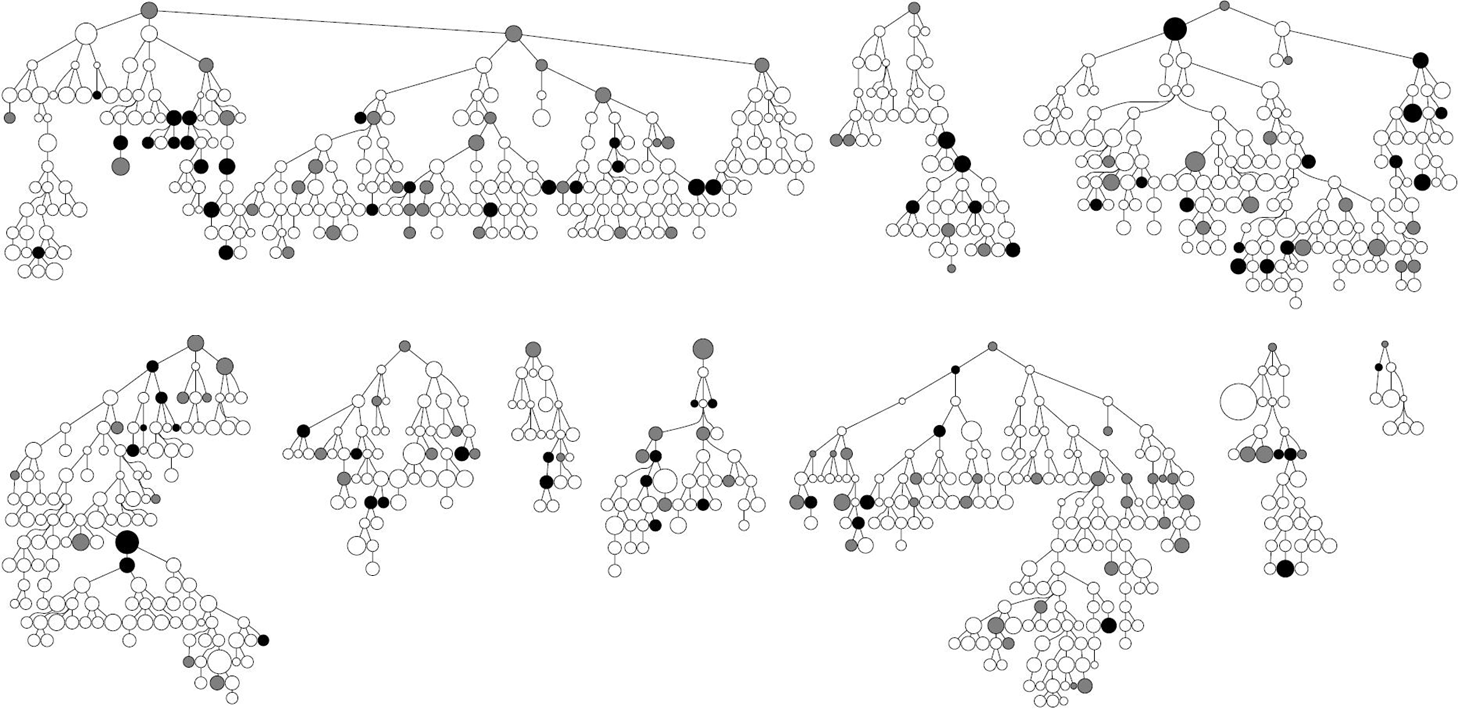


**Figure:** Recruitment networks showing HIV infection status, by seed. Seeds are shown at the top of each recruitment network. Symbol area is proportional to network size. HIV serostatus is shown by shading: black indicates HIV-positive; white, HIV-negative; gray, HIV status unknown. HIV status omitted for seeds for confidentiality. [24](#_ENREF_24)

**Explanation**

A graphic depiction of recruitment facilitates examination of clustering of behaviours or disease. A large number of seeds with short chains may result in unrepresentative data even if the sample size is large. In the event that long chains are not established, the characteristics of the seeds and how they were identified and selected becomes increasingly important (see also item 6a).

**13(f) Report recruitment challenges (e.g. commercial exchange of coupons, imposters, duplicate recruits) and how addressed**

**Explanation**

Issues warranting investigation and changes to coupons or incentives should be reported. These issues may include: the use of fake coupons, commercial exchange of coupons, or multiple recruits reporting having received a coupon from a stranger.

**13(g) Consider reporting estimated design effect for outcomes of interest**

**Example***“DE’s [Design effects] were calculated as the ratio of RDS variance to variance expected under SRS, as defined above. DE’s were calculated independently for each variable within each city.”* [88](#_ENREF_88)

**Explanation**

Currently most RDS studies assume a design effect of two when calculating the sample size needed to achieve study objectives. However, this is probably not sufficient . The estimated design effect based on study data may differ from the design effect that was assumed during study planning. Although all information needed to calculate the estimated design effect is included in the point estimate and confidence interval, explicitly presenting the estimated design effect calls attention to the uncertainty in the point estimate in a way that confidence intervals do not. Report the estimated design effect for the primary outcome of interest in order to provide a better understanding of whether the actual sample size was large enough to meet the study’s stated objectives along with estimates of homophily.

**14 Descriptive data**

**14(a) Give characteristics of study participants (e.g., demographic, clinical, social) and, if applicable, information on correlates and potential confounders. Report unweighted sample size and percentages, estimated population proportions or means with estimated precision (e.g., 95% confidence interval)**

**Example**


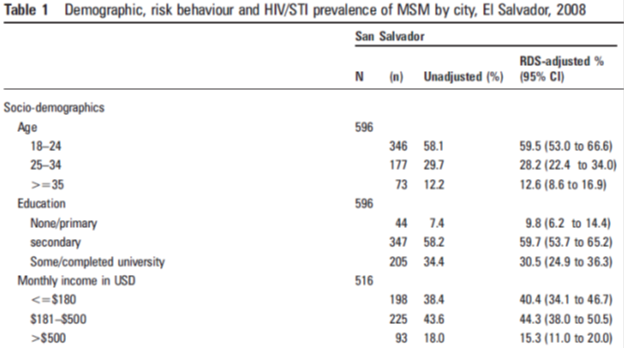


Source: [91](#_ENREF_91)

**Explanation**

Readers need descriptions of study participants to judge the generalisability of the findings. Information about potential confounders, including whether and how they were measured, influences judgments about study validity. We advise authors to summarize continuous variables for each study group by giving the mean and standard deviation, or when the data have an asymmetrical distribution, as is often the case, the median and percentile range (e.g., 25th and 75th percentiles). Variables that make up a small number of ordered categories (such as stages of disease I to IV) should not be presented as continuous variables; it is preferable to give numbers and proportions for each category. Descriptive characteristics and numbers should be given by group, as in the example above. Study sample results as well as study design-adjusted population results should be provided to facilitate comparison between study participants and population estimates. When quoting results, clearly specify if they are unadjusted or adjusted (see also item 16a). Consider reporting the distribution of personal network size for participants.

Inferential measures such as standard errors and confidence intervals should not be used to describe the variability of characteristics, and significance tests should be avoided in descriptive tables. Also, p-values are not an appropriate criterion for selecting which confounders to adjust for in analysis; even small differences in a confounder that has a strong effect on the outcome can be important.

**14(b) Indicate the number of participants with missing data for each variable of interest**

**Example 1**

***“****Seventy-one participants declined VCT and were thus missing the main outcome of interest.”*[30](#_ENREF_30)

**Example 2**

*“Of the 583 RDS participants, 47 (8.1%) refused syphilis testing, mostly (45/47, 95.7%) because they were recently tested.”* [43](#_ENREF_43)

**Explanation**

As missing data may bias or affect generalisability of results, authors should report any missing data on correlates, potential confounders, and other important characteristics of respondents (see also item 12d). We advise authors to use their tables and figures to enumerate amounts of missing data. For personal network size data be sure to report the number missing or with zero values, and how these were addressed.

**15 Outcome Data: If applicable, report number of outcome events or summary measures**

**Example**

**­**

**[…]**

**[…]**

*Source:*  [94](#_ENREF_94)

**Explanation**

Before addressing the possible association between exposures (risk factors) and outcomes, authors should report relevant descriptive data. It may be possible and meaningful to present measures of association in the same table that presents the descriptive data (see item 14a). Report the numbers of events for each outcome of interest. It may be helpful also to tabulate continuous outcomes or exposures in categories, even if the data are not analyzed as such.

**16 Main Results**

**16(a) Give unadjusted and study design adjusted estimates and, if applicable, confounder adjusted estimates and their precision (e.g., 95% confidence intervals). Make clear which confounders were adjusted for and why they were included**

**Example of effect of adjusting for study design**

*“Of the 624 MSM interviewed in the IBBS, 36 (5.8% crude; 3.2% adjusted RDS) had received the key chain”* [*95*](#_ENREF_95)

**Example of effect of adjusting for study design and confounding**

[…]

Source: [26](#_ENREF_26)

We could not find an example of an RDS study that showed clearly the sample description, *and* the study design adjusted (population) estimate *and* the study design and confounder adjusted estimate.

**Explanation**

Because of ongoing uncertainties regarding the ability of RDS estimators to reduce bias, we recommend reporting both unadjusted (sample description) and study design adjusted estimates (population estimates).

Further, if regression analysis is being carried out, we recommend reporting unadjusted (sample description), study design adjusted estimates (population estimates), *and* the study design and confounder adjustedanalyses. We advise giving the study design adjusted analyses together with the main data. This allows the reader to understand the data behind the measures of association (see also item 15). For study design and confounder -adjusted analyses, report the number of persons in the analysis, as this number may differ because of missing values in covariates (see also item 12d). Estimates should be given with confidence intervals. Readers can compare study design adjusted measures of association with those adjusted for study design and potential confounders and judge by how much, and in what direction, they changed. Readers may think that ‘fully adjusted’ results imply causality for the measure of association, but adjusted results are not necessarily free of random sampling error, selection bias, information bias, or residual confounding. Thus, great care should be exercised when interpreting adjusted results, as the validity of results often depends crucially on complete knowledge of important confounders, their precise measurement, and appropriate specification in the statistical model (see also item 20).

Authors should explain all potential confounders considered, and the criteria for excluding or including variables in statistical models. Decisions about excluding or including variables should be guided by knowledge, or explicit assumptions, on causal relations. Inappropriate decisions may introduce bias, for example by including variables that are in the causal pathway between exposure and disease unless the aim is to assess how much of the effect is carried by the intermediary variable. If the decision to include a variable in the model was based on the change in the estimate, it is important to report what change was considered sufficiently important to justify its inclusion. If a ‘backward deletion’ or ‘forward inclusion’ strategy was used to select confounders, explain that process and give the significance level for rejecting the null hypothesis of no confounding. Of note, we and others do not advise selecting confounders based solely on statistical significance testing.

**16(b) Report category boundaries when continuous variables were categorised**

**Example**

*“Household socioeconomic status was calculated using principal components analysis from household ownership of 22 items recorded during an annual census (December 2008-October 2009) and categorised into quartiles based on the status of all households in the general population cohort villages.“* [100](#_ENREF_100)

**Explanation**

Categorizing continuous data has several important implications for analysis and also affects the presentation of results. In tables, outcomes should be given for each exposure category, for example as counts of persons at risk. Details of the categories used may aid comparison of studies and meta-analysis. If data were grouped using conventional cut-points group boundaries (i.e., range of values) can be derived easily, except for the highest and lowest categories. If quantile-derived categories are used, the category boundaries cannot be inferred from the data. As a minimum, authors should report the category boundaries; it is helpful also to report the range of the data and the mean or median values within categories.

**16(c) If adjustment of primary outcome leads to marked changes, report information on factors influencing the adjustments (e.g. personal network sizes, recruitment patterns by group, key confounders)**

**Example**

*“[T]here was a positive association between the sex of the recruiter and the sex of the recruit, and, independently of this association, syphilis antibody positive women were disproportionately less likely to recruit syphilis positive men than syphilis negative men.”*  [33](#_ENREF_33)

**Explanation**

If the unadjusted (sample description), study-design adjusted (population estimate) or study-design adjusted *and* confounder adjusted estimates are markedly different, researchers should strive to explain the cause of these differences. At a minimum, researchers should report information that might suggest the sources of these differences. For example, if researchers use the RDS I estimator, they should report the cross-group recruitment matrix and the estimated average degree for each group. If adjusting for confounding caused the marked differences, key confounders should be highlighted.

**17 Other Analyses: Report other analyses done—e.g., analyses of subgroups and interactions, sensitivity analyses, different RDS estimators, and definitions of personal network size**

**Example**

*“Sensitivity analyses were used to assess the robustness of our results to various network size definitions, potential network-size bias, and respondent-driven sampling sample size. […] However, sensitivity analysis found no evidence that collecting detailed network data reduced the performance of the respondent-driven sampling estimators”* [24](#_ENREF_24)

**Explanation**

As RDS analytic methods are still being refined it is useful to report which RDS estimators were used and why. Presenting results using different estimators can help advance the method and facilitate a more comprehensive analysis of the data.

Different recall periods, and the timing (i.e., first or second visit) and how personal network size questions are asked will result in different self-reported personal network sizes and may lead to different estimates (see also item 6c and 6d). Investigation of novel approaches to collecting personal network size information and the effect of the recall period on test-retest reliability (e.g. [79](#_ENREF_79)) is ongoing.

In addition to the main analysis done for RDS studies, other analyses are often done in observational studies. They may address specific subgroups, the potential interaction between risk factors, the calculation of attributable risks, or use alternative definitions of study variables in sensitivity analyses. There is debate about the dangers associated with subgroup analyses, and multiplicity of analyses in general. In our opinion, there is too great a tendency to look for evidence of subgroup-specific associations, or effect-measure modification, when overall results appear to suggest little or no effect. On the other hand, there is value in exploring whether an overall association appears consistent across several, preferably pre-specified subgroups especially when a study is large enough to have sufficient data in each subgroup. A second area of debate is about interesting subgroups that arose during the data analysis. They might be important findings, but might also arise by chance. Some argue that it is neither possible nor necessary to inform the reader about all subgroup analyses done as future analyses of other data will tell to what extent the early exciting findings stand the test of time. [101](#_ENREF_101) We advise authors to report which analyses were planned, and which were not (see also items 4, 12c and 20). This will allow readers to judge the implications of multiplicity, taking into account the study’s position on the continuum from discovery to verification or refutation.

A third area of debate is how joint effects and interactions between risk factors should be evaluated: on additive or multiplicative scales, or should the scale be determined by the statistical model that fits best (see also item 12c)? A sensible approach is to report the separate effect of each exposure as well as the joint effect – if possible in a table. Such a table gives the reader sufficient information to evaluate additive as well as multiplicative interaction. Confidence intervals for separate and joint effects may help the reader to judge the strength of the data. In addition, confidence intervals around measures of interaction, such as the Relative Excess Risk from Interaction (RERI) relate to tests of interaction or homogeneity tests. One recurrent problem is that authors use comparisons of p-values across subgroups, which lead to erroneous claims about an effect modifier. For instance, a statistically significant association in one category (e.g., men), but not in the other (e.g., women) does not in itself provide evidence of effect modification. Similarly, the confidence intervals for each point estimate are sometimes inappropriately used to infer that there is no interaction when intervals overlap. A more valid inference is achieved by directly evaluating whether the magnitude of an association differs across subgroups.

Sensitivity analyses are helpful to investigate the influence of choices made in the statistical analysis, or to investigate the robustness of the findings to missing data or possible biases (see also item 12c). Judgment is needed regarding the level of reporting of such analyses. If many sensitivity analyses were performed, it may be impractical to present detailed findings for them all. It may sometimes be sufficient to report that sensitivity analyses were carried out and that they were consistent with the main results presented. Detailed presentation is more appropriate if the issue investigated is of major concern, or if effect estimates vary considerably.

**DISCUSSION (Items 18 - 21)**

The discussion section addresses the central issues of validity and meaning of the study. Studies have found that discussion sections are often dominated by incomplete or biased assessments of the study’s results and their implications, and rhetoric supporting the authors’ findings. Structuring the discussion may help authors avoid unwarranted speculation and over-interpretation of results while guiding readers through the text. For example, Annals of Internal Medicine recommends that authors structure the discussion section by presenting the following: 1) a brief synopsis of the key findings; 2) consideration of possible mechanisms and explanations; 3) comparison with relevant findings from other published studies; 4) limitations of the study; and 5) a brief section that summarizes the implications of the work for practice and research. Others have made similar suggestions. The section on research recommendations and the section on limitations of the study should be closely linked to each other. Investigators should suggest ways in which subsequent research can improve on their studies rather than blandly stating ‘more research is needed’. We recommend that authors structure their discussion sections, perhaps also using suitable subheadings.

**18 Key Results: Summarise key results with reference to study objectives**

**Example**

*“These are the first data from a large-scale quantitative study using RDS methodology to investigate risk factors including injection drug use associated with HIV among MSM in Africa. Eleven percent of non-injecting MSM in Zanzibar were HIV-infected; a quarter of MSM-IDU were infected with either HIV or HCV and among those, 30% were infected with both HIV and HCV. Men in Zanzibar who practice male-to-male sex are known to be at increased risk for acquiring and transmitting HIV (Dahoma et al., 2009). These data suggest that MSM who inject drugs are not only at increased risk for HIV injection through their injection drug use but also from several sexual risk behaviours including sex with multiple partners”.*[110](#_ENREF_110)

**Explanation**

It is good practice to begin the discussion with a short summary of the main findings of the study. The short summary reminds readers of the main findings and may help them assess whether the subsequent interpretation and implications offered by the authors are supported by the findings.

**19 Limitations: Discuss limitations of the study, taking into account sources of potential bias or imprecision. Discuss both direction and magnitude of any potential bias**

**Example 1**

*“A further limitation is that a significant proportion of MSM declined HIV testing (18 of 101). Analysis of risk behaviors among those who opted out indicates a trend toward higher level of risk behaviors among those who opted out, but the difference was not statistically significant, possibly due to the small total sample size (data not shown). The very high reported condom use by FSWs raises the question of potential reporting biases among this group, given that it is higher than that in an earlier study that found approximately only a third out of a sample of 539 sex workers reported always using condoms with nonregular and regular clients.”* [111](#_ENREF_111)

**Example 2**

*“Furthermore, the findings may be limited by the fact that only a small absolute number of MSM in this sample reported injecting drugs in the previous 3 months; therefore, we lack power to identify some factors that may be associated with injection drug use and final estimates should be interpreted with caution. Some questions about injection drug use referred only to the previous 3 months and did not assess lifetime history of injection drug use among MSM limiting any associations with injection drug use in this analysis to activity in the past 3 months.”* [110](#_ENREF_110)

**Explanation**

The identification and discussion of the limitations of a study are an essential part of scientific reporting. It is important not only to identify the sources of bias and confounding that could have affected results, but also to discuss the relative importance of different biases, including the likely direction and magnitude of any potential bias (see also item 9).

To understand the extent to which recruitment may have penetrated all sub-groups and study participants reflect the target population, authors can compare the sample socio-demographic data to that collected during the formative assessment or previous research. Formative assessment data may also explain if and why the demographic characteristics of the study sample do not adequately reflect the population. Comparing the RDS sample and population estimates to the population description obtained in the formative assessment may also facilitate identification of any sampling biases, although the usefulness of this comparison for this purpose is not unanimously accepted by RDS methodologists. We recommend investigators report on any high homophily or heterophily (and how these were measured), inability to reach equilibrium on key variables, and recruitment bottlenecks and clustering of key variables in recruitment networks.

Authors should also discuss any imprecision of the results. Imprecision may arise in connection with several aspects of a study, including the study size (item 10) and the measurement of exposures, confounders and outcomes (item 8). The inability to precisely measure true values of an exposure tends to result in bias towards unity: the less precisely a risk factor is measured, the greater the bias. This effect has been described as ‘attenuation’, or more recently as ‘regression dilution bias’.[114](#_ENREF_114) However, when correlated risk factors are measured with different degrees of imprecision, the adjusted relative risk associated with them can be biased towards or away from unity. [115-117](#_ENREF_115)

When discussing limitations, authors may compare the study being presented with other studies in the literature in terms of validity, generalisability and precision. In this approach, each study can be viewed as contribution to the literature, not as a stand-alone basis for inference and action. [118](#_ENREF_118) Surprisingly, the discussion of important limitations of a study is sometimes omitted from published reports.

**20 Interpretation: Give a cautious overall interpretation of results considering objectives, limitations, multiplicity of analyses, results from similar studies, and other relevant evidence**

**Example**

*“In comparison with the Multicentric Study conducted in 2001, we found a similar HIV prevalence among FSWs in Tegucigalpa (5.4% in 2006 versus 6.8% in 2001), but a lower prevalence in San Pedro Sula (3.7% versus 13.0%). Surveys of FSWs in Tegucigalpa in 1989, 1996 and 1998 reported HIV prevalence of 5.5%, 8.0% […]. However, substantial differences in methodology between the surveys limit comparability and it is impossible to establish actual trends.”* [119](#_ENREF_119)

**Explanation**

The heart of the discussion section is the interpretation of a study’s results. Over-interpretation is common and human: even when we try hard to give an objective assessment, reviewers often rightly point out that we went too far in some respects. When interpreting results, authors should consider the nature of the study on the discovery to verification continuum and potential sources of bias, including loss to follow-up and non-participation (see also items 9, 12 and 19). Due consideration should be given to confounding (item 16a), the results of relevant sensitivity analyses, and to the issue of multiplicity and subgroup analyses (item 17). Authors should also consider residual confounding due to unmeasured variables or imprecise measurement of confounders. For example, socioeconomic status (SES) is associated with many health outcomes and often differs between groups being compared. Variables used to measure SES (income, education, or occupation) are surrogates for other undefined and unmeasured exposures, and the true confounder will by definition be measured with error. [120](#_ENREF_120) Authors should address the real range of uncertainty in estimates, which is larger than the statistical uncertainty reflected in confidence intervals. The latter do not take into account other uncertainties that arise from a study’s design, implementation, and methods of measurement. [121](#_ENREF_121)

A discussion of the existing external evidence, from different types of studies, should always be included, but may be particularly important for studies reporting small increases in risk. Further, authors should put their results in context with similar studies and explain how the new study affects the existing body of evidence, ideally by referring to a systematic review.

**21 Generalisability: Discuss the generalisability (external validity) of the study results**

**Example**

*“[I]t is possible that men who are unemployed and who have lower socioeconomic status (SES) and educational achievement are not well networked with better educated and resourced MSM. Thus unemployed men of lower SES may be overrepresented in our sample. However, our results are likely representative of the most socially vulnerable MSM whose limited income, mobility, and economic opportunity may also limit their access to HIV prevention information, condoms and latex-compatible lubricant, and high-quality health services. It is notable that although black race and Soweto residence were not explicit inclusion criteria, the chains did not leave Soweto except in the case of one Coloured participant, suggesting that the legacy of apartheid continues to limit Soweto MSM’s geographic and social mobility.”* [30](#_ENREF_30)

**Explanation**

Generalisability, also called external validity or applicability, is the extent to which the results of a study can be applied to other circumstances.[122](#_ENREF_122) There is no external validity per se; the term is meaningful only with regard to clearly specified conditions.[123](#_ENREF_123) Can results be applied to an individual, groups or populations that differ from those enrolled in the study with regard to age, sex, ethnicity, severity of disease, and co-morbid conditions? Are the nature and level of exposures comparable, and the definitions of outcomes relevant to another setting or population?

The question of whether the results of a study have external validity is often a matter of judgment that depends on the study setting, the characteristics of the participants, the exposures examined, and the outcomes assessed. Thus, it is crucial that authors provide readers with adequate information about the setting and locations, eligibility criteria, the exposures and how they were measured, the definition of outcomes, and the period of recruitment and follow-up. The degree of nonparticipation and the proportion of unexposed participants in whom the outcome develops are also relevant. Knowledge of the absolute risk and prevalence of the exposure, which will often vary across populations, are helpful when applying results to other settings and populations.

**OTHER INFORMATION (Item 22)**

**22 Funding: Give the source of funding and the role of the funders for the present study and, if applicable, for the original study on which the present article is based**

**Example**

***“****This research was supported by the National Institute on Drug Abuse (R01 DA019964-01, T32 DA007282). The funder had no role in study design, data collection and analysis, decision to publish, or preparation of the manuscript.”* [124](#_ENREF_124)

**Explanation**

Some journals require authors to disclose the presence or absence of financial and other conflicts of interest. Several investigations show strong associations between the source of funding and the conclusions of research articles. [126-129](#_ENREF_126)

Authors or funders may have conflicts of interest that influence any of the following: the design of the study [130](#_ENREF_130); choice of exposures, outcomes, [132](#_ENREF_132) statistical methods, [133](#_ENREF_133) and selective publication of outcomes[132](#_ENREF_132) and studies. [134](#_ENREF_134) Consequently, the role of the funders should be described in detail: in what part of the study they took direct responsibility (e.g., design, data collection, analysis, drafting of the manuscript, decision to publish). [66](#_ENREF_66) Other sources of undue influence include employers (e.g., university administrators for academic researchers and government supervisors, especially political appointees, for government researchers), advisory committees, litigants, and special interest groups.

**BOXES 1-8**

- For topics that pertain to more than one checklist item
- See main manuscript text for a box on ’*Respondent-Driven Sampling Key Terms And Definitions’*

| **Box 1- Biases in RDS** |
| --- |
| Bias is a systematic deviation of a study's result from a true value. Typically, it is introduced during the design or implementation of a study and cannot be remedied later. Bias and confounding are not synonymous. Bias arises from flawed information or subject selection so that a wrong association is found. Confounding produces relations that are factually right, but that cannot be interpreted causally because some underlying, unaccounted for factor is associated with both exposure and outcome (see Box 3). Also, bias needs to be distinguished from random error, a deviation from a true value caused by statistical fluctuations (in either direction) in the measured data. Many possible sources of bias have been described and a variety of terms are used [135](#_ENREF_135). We find two simple categories helpful: information bias and selection bias.  Information bias occurs when there are systematic differences in the completeness or the accuracy of information about respondents. For instance, if pregnant women are more likely to be tested for HIV than other women, the ascertainment of HIV status by self-report may be more complete in pregnant women. Information bias may occur in RDS studies; for example if HIV positive individuals recall past risk behaviour more or less accurately than those who do not know if they are HIV positive (also called recall bias). Interviewer bias can be problematic in RDS studies; for example if interviewers use different techniques when asking questions, particularly those related to personal network size. Recommendations for reducing this bias during study implementation include using only one person to ask network size questions and/or training interviewers to use uniform techniques for administering such questions.  Selection bias may occur if the estimated selection probability is different from the actual selection probability. One form of selection bias may be caused by recruitment bias: unaccounted for patterns in peer recruitment. For example, if recruiters recruit peers who have higher levels of HIV risk behaviour and this pattern is not accounted for by the RDS estimator, then the estimated probabilities of selection will be biased and the resulting estimates of HIV risk behaviour will be too high. An additional form of selection bias might be caused by differential non-response (also called ‘response bias’): if there are systematic differences between those who agree and decline recruitment. For example, in RDS studies if those who live near the study site are more likely to accept a coupon [100](#_ENREF_100) and are systematically different from those living far away from the study site, then the estimates could be biased unless this pattern was accounted for in the RDS estimator. |

| **Box 2- Grouping** |
| --- |
| There are several reasons why continuous data may be grouped. When collecting data it may be better to use an ordinal variable than to seek an artificially precise continuous measure for an exposure based on recall over several years. Categories may also be helpful for presentation, for example to present all variables in a similar style.  Grouping may also be done to simplify the analysis, for example to avoid an assumption of linearity. However, grouping loses information and may reduce statistical power especially when dichotomization is used . If a continuous confounder is grouped, residual confounding may occur, whereby some of the variable’s confounding effect remains unadjusted for (see Box 3) . Increasing the number of categories can diminish power loss and residual confounding, and is especially appropriate in large studies. Small studies may use few groups because of limited numbers. For some RDS estimators, data need to be grouped to derive estimates using transition probabilities. Having too many groups, especially if those groups contain empty cells in a recruitment matrix, will likely result in unstable estimators. Some software smooths data to facilitate calculating the estimates when recruitment matrices have small or empty cells.  Investigators may choose cut-points for groupings based on commonly used values that are relevant for the type of data being analyzed or on statistical grounds. They may choose equal numbers of individuals in each group using quantiles [139](#_ENREF_139). On the other hand, one may gain more insight into the association with the outcome by choosing more extreme outer groups and having the middle group(s) larger than the outer groups [140](#_ENREF_140). Readers should be informed if cut-points are selected post hoc from several alternatives. In particular, if the cut-points were chosen to minimize a P value the true strength of an association will be exaggerated [141](#_ENREF_141).  When analyzing grouped variables, it is important to recognize their underlying continuous nature. For instance, a possible trend in risk across ordered groups can be investigated. A common approach is to model the rank of the groups as a continuous variable. Such linearity across group scores will approximate an actual linear relation if groups are equally spaced (e.g., 10 year age groups) but not otherwise. Il’yasova et al [142](#_ENREF_142) recommend publication of both the categorical and the continuous estimates of effect, with their standard errors, in order to facilitate meta-analysis. One analysis may inform the other and neither is assumption-free. Authors often ignore the ordering and consider the estimates (and p-values) separately for each category compared to the reference category. This may be useful for description, but may fail to detect a real trend in risk across groups. If a trend is observed, a confidence interval for a slope might indicate the strength of the observation. |

| **Box 3- Confounding** |
| --- |
| Confounding literally means confusion of effects. A study might seem to show either an association or no association between an exposure and the risk of a disease. In reality, the seeming association or lack of association is due to another factor that determines the occurrence of the disease but that is also associated with the exposure. The other factor is called the confounding factor or confounder. Confounding thus gives a wrong assessment of the potential ‘causal' association of an exposure. For example, if women who approach middle age and develop elevated blood pressure are less often prescribed oral contraceptives, a simple comparison of the frequency of cardiovascular disease between those who use contraceptives and those who do not, might give the wrong impression that contraceptives protect against heart disease.  Any sampling procedure where respondents are sampled with unequal probabilities can confound analyses of association if the analysis does not explicitly adjust for the sampling design. If the probability of inclusion in the sample is associated with both variables being analyzed, it can serve as a confounder of the relationship between the two [143](#_ENREF_143). For example, if HIV-positive women have a higher probability of being sampled than other types of respondents, an unadjusted analysis of the association between gender and HIV might erroneously conclude that women are more likely to have HIV than men even though the observed difference is solely due to the sampling design.  The RDS sampling process introduces additional complications into assessing the association between a risk factor and disease risk because the sample inclusion probabilities are a complex function of the recruitment behaviour of respondents and the unobserved social network connecting respondents [44](#_ENREF_44). In particular, homophily (the tendency for people to have ties to those similar to themselves) may act as a confounder in some situations . If, for example, HIV-positive women have high homophily but HIV-negative women and HIV-positive men do not, HIV-positive women will be over-represented in the sample. An analysis of the association between gender and HIV that does not adjust for these differences in homophily might incorrectly conclude that women are more likely to have HIV than men even though the observed difference is solely due to differential homophily and its impact on HIV-positive women’s sample inclusion probabilities.  Investigators should think beforehand about potential confounding factors and the impact of RDS on their analyses. This will inform the study design and allow proper data collection by identifying the confounders for which detailed information should be sought. Restriction or matching may be used. In the blood pressure example above, the study might be restricted to women who do not have the confounder, elevated blood pressure. Matching on blood pressure might also be possible, though not necessarily desirable. In the analysis phase, investigators may use stratification or multivariable analysis to reduce the effect of confounders. Stratification consists of dividing the data in strata for the confounder (e.g., strata of blood pressure), assessing estimates of association within each stratum, and calculating the combined estimate of association as a weighted average over all strata. Multivariable analysis achieves the same result but permits one to take more variables into account simultaneously. It is more flexible but may involve additional assumptions about the mathematical form of the relationship between exposure and disease. As data collected using RDS has less statistical power than data collected using simple random sampling, model standard errors may be too narrow and overstate the model’s precision if the standard errors are not adjusted to account for the sampling design .  Taking confounders into account is crucial in observational studies, but readers should not assume that analyses adjusted for confounders establish the ‘causal part' of an association. Results may still be distorted by residual confounding (the confounding that remains after unsuccessful attempts to control for it [145](#_ENREF_145)), sampling error, selection bias and information bias (see Box 1). |

| **Box 4- Missing Data** |
| --- |
| A common approach to dealing with missing data is to restrict analyses to individuals with complete data on all variables required for a particular analysis. Although such ‘complete-case’ analyses are unbiased in many circumstances, they can be biased and are always inefficient [146](#_ENREF_146). Bias arises if individuals with missing data are not typical of the whole sample. Furthermore, in RDS analysis, which relies on network connections between recruiters and their recruits, eliminating those with missing data would interrupt the recruitment chains, negatively impacting estimates; another inefficiency arises because of the reduced sample size for analysis.  An important concern in RDS is missing personal network size data. This is particularly important because of the role that personal network size plays in most existing estimators. Researchers should describe if there are missing values for personal network size and how this was handled in the analysis.  Using the last observation carried forward for repeated measures can distort trends over time if persons who experience a foreshadowing of the outcome selectively drop out [147](#_ENREF_147). Inserting a missing category indicator for a confounder may increase residual confounding [71](#_ENREF_71). Imputation, in which each missing value is replaced with an assumed or estimated value, may lead to attenuation or exaggeration of the association of interest, and without the use of sophisticated methods described below may produce standard errors that are too small.  Rubin developed a typology of missing data problems, based on a model for the probability of an observation being missing . Data are described as missing completely at random (MCAR) if the probability that a particular observation is missing does not depend on the value of any observable variable(s). Data are missing at random (MAR) if, given the observed data, the probability that observations are missing is independent of the actual values of the missing data. For example, suppose younger children are more prone to missing spirometry measurements, but that the probability of missing is unrelated to the true unobserved lung function, after accounting for age. Then the missing lung function measurement would be MAR in models including age. Data are missing not at random (MNAR) if the probability of missing still depends on the missing value even after taking the available data into account. When data are MNAR valid inferences require explicit assumptions about the mechanisms that led to missing data.  Methods to deal with data missing at random (MAR) fall into three broad classes : likelihood-based approaches [150](#_ENREF_150), weighted estimation [151](#_ENREF_151) and multiple imputation . Of these three approaches, multiple imputation is the most commonly used and flexible, particularly when multiple variables have missing values . Results using any of these approaches should be compared with those from complete case analyses, and important differences discussed. The plausibility of assumptions made in missing data analyses is generally unverifiable. In particular it is impossible to prove that data are MAR, rather than MNAR. Such analyses are therefore best viewed in the spirit of sensitivity analysis (see items 12e and 17). |

| **Box 5- Measures of association, effect and impact** |
| --- |
| Observational studies may be solely done to describe the magnitude and distribution of a health problem in the population. They may examine the number of people who have a disease at a particular time (prevalence), or that develop a disease over a defined period (incidence). The incidence may be expressed as the proportion of people developing the disease (cumulative incidence) or as a rate per person-time of follow-up (incidence rate). Specific terms are used to describe different incidences; amongst others, mortality rate, birth rate, attack rate, or case fatality rate. Similarly, terms like point prevalence and period, annual or lifetime prevalence are used to describe different types of prevalence [155](#_ENREF_155).  Most cross-sectional studies, including RDS studies, do not estimate incidence rates, although it is sometimes possible to do if certain (potentially untestable) assumptions are made [156](#_ENREF_156). Associations based on prevalence may be estimated as “prevalence ratios” (ratios of prevalence) or “prevalence odds ratios" (ratio of the odds of disease between groups being compared). Logistic regression generalizes the odds ratio to the multivariate case, so estimates from logistic regression models are reported in terms of odds ratios [157](#_ENREF_157).  Producing estimates of attributable fraction or absolute risk generally requires information about the population external to the study, such as the size of the population and its characteristics. Most RDS studies examine populations about which little is known due to their hidden status [158](#_ENREF_158). This lack of external information makes calculation of absolute measures even more challenging for RDS studies than it would be for studies conducted using sampling methods not designed for hidden populations. |

| **Box 6- Interaction (effect modification)** |
| --- |
| RDS complicates estimations of association (see Boxes 3 and 5). Interaction exists when the association of an exposure with the risk of disease differs in the presence of another exposure. As described in Box 3, the RDS sampling design can interfere with estimation of association, so estimates may be biased if the analysis does not adjust for the sampling design [148](#_ENREF_148). Because detecting interaction is more complex (and demands more of the data) than estimating either prevalence or bivariate association, adjusting estimates of interaction for the RDS sampling design requires additional effort and care [159](#_ENREF_159). Authors should report all steps they took to adjust estimates of interaction for the RDS sampling design and consider carefully the possible effects of RDS on their estimates.  As discussed in Box 5, most RDS studies produce only relative measures of risk. One problem in evaluating and reporting interactions is that the effect of an exposure can be measured in two ways: as a relative risk (or rate ratio) or as a risk difference (or rate difference). The use of the relative risk leads to a multiplicative model, while the use of the risk difference corresponds to an additive model . A distinction is sometimes made between ‘statistical interaction' which can be a departure from either a multiplicative or additive model, and ‘biologic interaction' which is measured by departure from an additive model [162](#_ENREF_162). However, neither additive nor multiplicative models point to a particular biologic mechanism.  Regardless of the model choice, the main objective is to understand how the joint effect of two exposures differs from their separate effects (in the absence of the other exposure).  The Human Genomic Epidemiology Network (HuGENet) proposed a lay-out for transparent presentation of separate and joint effects that permits evaluation of different types of interaction [163](#_ENREF_163). |

| **Box 7- WebRDS and e-coupons** | |
| --- | --- |
| RDS studies whose recruitment occurs electronically operate in a very different environment that warrants the reporting of additional study characteristics. Geographic distance becomes less relevant and coupon transfer from recruiter to recruit may occur instantaneously with minimal effort. Literacy, internet penetration, and frequency of internet use may alter sampling and recruitment patterns. Verification of membership in the target population is extremely difficult in webRDS studies. Without proper design measures, electronic coupons may be easily copied, forwarded to several candidate recruits simultaneously, or forwarded serially through several persons. The risk of repeat enrolment may be increased in webRDS studies and its detection more difficult. IP addresses may constitute computer identifiers and communication between the study server and the respondent’s computer may warrant encryption to ensure confidentiality.  If reporting on a webRDS we suggest reporting additional information for the following checklist items: | |
| **Existing checklist item** | **Additional information to report** |
| 5a | Describe efforts to maintain respondent anonymity, as warranted |
| 5b | Indicate extent of internet or mobile phone access for target population and literacy rate |
| 8b | Describe efforts to prevent multiple use or repeat forwarding of electronic coupons |
| 14a | Describe frequency of internet use among study respondents and how this might have affected participants' inclusion probabilities. |
|  | Describe respondents’ distribution across space, e.g., across counties, districts, provinces etc. |
| **5a Examples**  Methods: *“IP addresses were converted to a unique anonymous code using a one-way encryption algorithm, and the original IP numbers were deleted. Login passwords were only valid for a single session, and communication between the users and the server was encrypted.”* [164](#_ENREF_164)  **5b Examples:**  Methods: *“Internet use as a proportion of the population in Vietnam was 27% in 2010 (24 million persons) and 60% and 50%, respectively, in the large urban areas of Hanoi and Ho Chi Minh City. Internet use among MSM in general may be considerably higher than in the general population. Ninety four percent of MSM in an offline RDS in Hanoi stated that they used the Internet.”* [164](#_ENREF_164)  **8b Examples:**  Methods:*“We checked all surveys for other signs of duplication or invalidity by flagging surveys containing a repeated IP number.”*  Results: *“9.6% of completed surveys (65 surveys) included a stated age below 18 years, or a telephone number, e-mail or Yahoo! Chat address that had previously been registered in the system. We defined these as “invalid”.”* [164](#_ENREF_164)  **14a Examples:**  Results: *“An estimated 67% used the Internet every day during the past month and an estimated 82% came from the two large metropolitan areas of Ho Chi Minh City and Hanoi (81% of the sample). The recruitment trees also penetrated outside the large metropolitan areas with 32 provinces represented out of 63 (Fig. 3a-c).”* [164](#_ENREF_164) | |

| **Box 8- Serial studies and combining data sets** |
| --- |
| When multiple RDS studies are conducted in the same population over time (e.g., repeated panel study [166](#_ENREF_166)) or over multiple populations at the same time (e.g., concurrent behavioural surveillance studies of MSM in 10 Brazilian cities [25](#_ENREF_25)) additional complexities arise.    The most common reason for conducting repeated cross-sectional studies in the same population is to monitor trends in risk behaviour and disease prevalence in order to evaluate public health interventions. When conducting these studies, we recommend that researchers be especially sensitive to two potential problems. First, researchers should be cautious about features of these studies that will make the estimates non-independent. For example, using participants from one year as seeds in future studies may result in the under-estimation of changes in the target population. Using the same study site across time may also lead researchers to under-estimate changes in the target population. Second, researchers should be cautious about changes in study procedures over time. For example, altering the recruitment incentive between studies may lead researchers to over-estimate changes in the target population. It is important to recognize that the above two concerns may conflict. For example, in order to reduce dependence between studies, researchers may wish to use different study sites each year. However, such changes would also render it more complex to interpret any differences over time. These concerns must be balanced on a case-by-case basis.  Investigators may also simultaneously conduct multiple studies of a single group in different settings. After conducting these studies, researchers often want to combine estimates into an aggregate estimate. For example, researchers estimated HIV prevalence of female sex workers (FSW) in 10 Brazilian cities and wished to combine these estimates to produce an aggregate estimate. [52](#_ENREF_52) [56](#_ENREF_56) Researchers should be sensitive to two potential problems with such an analysis. First, combining estimates from different cities requires researchers to know the sizes of the target population in each city (e.g., the number of FSW). The uncertainty that often exists in the population sizes represents an additional source of error in the aggregated estimate. Second, the aggregated estimates will only reflect members of the target population in the cities that were studied. Therefore, care should be taken in calling these “national” estimates. For example, if FSW in cities not included in the study have a lower HIV prevalence, the aggregated estimate from the 10 cities will not represent a national estimate. |

1. Department of Infectious Disease Epidemiology, London School of Hygiene and Tropical Medicine, UK ([Richard.White@lshtm.ac.uk](mailto:Richard.White@lshtm.ac.uk); [kate.orroth@gmail.com](mailto:kate.orroth@gmail.com) ) [↑](#footnote-ref-2)
2. US Centers for Disease Control and Prevention. Atlanta, GA, USA ([hxv8@cdc.gov](mailto:hxv8@cdc.gov); [ajd9@cdc.gov](mailto:ajd9@cdc.gov); [wfh3@cdc.gov](mailto:wfh3@cdc.gov), [wfu3@cdc.gov](mailto:wfu3@cdc.gov) ) [↑](#footnote-ref-3)
3. Department of Sociology and Office of Population Research, Princeton University ([mjs3@princeton.edu](mailto:mjs3@princeton.edu)) [↑](#footnote-ref-4)
4. Department of Global Community Health and Behavioral Sciences, Tulane University School of Public Health and Tropical Medicine; Global Health Science, University of California San Francisco, USA ( [lsjohnston.global@gmail.com](mailto:lsjohnston.global@gmail.com)) [↑](#footnote-ref-5)
5. Departamento de Saude Comunitaria, Universidade Federal do Ceara, Fortaleza, Ceara, Brazil ([ligiakerr@gmail.com](mailto:ligiakerr@gmail.com)) [↑](#footnote-ref-6)
6. Department of Global Community Health and Behavioral Sciences, Tulane University School of Public Health and Tropical Medicine ([ckendall@tulane.edu](mailto:ckendall@tulane.edu)) [↑](#footnote-ref-7)
7. World Bank, USA ([dwilson@worldbank.org](mailto:dwilson@worldbank.org) ) [↑](#footnote-ref-8)
8. Institute of Social & Preventive Medicine (ISPM), University of Bern, Switzerland ([Matthias.egger@ispm.unibe.ch](mailto:Matthias.egger@ispm.unibe.ch)) [↑](#footnote-ref-9)
